# Supplementary material for: Precise recognition of benzonitrile derivatives with supramolecular macrocycle of phosphorylated cavitand by co-crystallization method
Source: Nat Commun. 2024 Jun 22;15:5315. doi: 10.1038/s41467-024-49540-2 (PMC11193764; doi:10.1038/s41467-024-49540-2)
Supplement: Supplementary file 1 — Supplementary Information [file 41467_2024_49540_MOESM1_ESM.pdf]

Supplementary Information for

Precise recognition of benzonitrile derivatives with supramolecular macrocycle of phosphorylated cavitand by co-crystallization method

Heng Li,[1] Zhijin Li, [1] Chen Lin, [1]* Juli Jiang, [1]* Leyong Wang

[1] State Key Laboratory of Analytical Chemistry for Life Science, Jiangsu Key Laboratory of Advanced Organic Materials, School of Chemistry and Chemical Engineering, Nanjing University, Nanjing, 210023, China

Contents

1. Supplementary Methods 3

2. Supplementary Discussion.. 5

2.1 Synthesis and Characterization 5

2.2 NMR and HRMS Spectra 9

2.3 Electrostatic potential surface of F[3]A1-[P(O)Ph]$_3$ 15

2.4. The single crystal structure of dichloromethane @ F[3]A1-[P(O)Ph]$_3$ 16

2.5. The single crystal structure of co-crystal 18

2.6. IGMH analysis 40

2.7. ^{1}H NMR spectra of titration experiment details between benzonitrile and F[3]A1-[P(O)Ph]$_3$. 42

2.8. UV-vis titration experiments details between guest (G1-G15) and F[3]A1-[P(O)Ph]$_3$. 44

2.9. 2D Hirschfeld fingerprinting figure 60

2.10. Electron cloud density map 72

2.11. Details of DFT calculations 75

3. Supplementary References...84

1. Supplementary Methods

All reactions were performed in an air atmosphere unless otherwise stated. Deuterium solvents were purchased from Aldrich. All other reagents were obtained from commercial sources and were used without further purification unless indicated otherwise. All yields were given as isolated yields. ^{1}H **NMR** and ^{13}C **NMR** spectra were recorded on a **BRUKER AVANCE III** 400 **MHz**, and the chemical shifts (δ) for **^{1}H NMR** spectra, given in ppm, are referenced to the residual proton signal of the deuterated solvent. UV-vis titration experiments were performed using a UV-vis near infrared spectrophotometer (Shimadzu UV-1780). Diffraction data were collected on an **X-ray** single crystal diffractometer at **Bruker AXS GmbH**, Germany, at 193 K using **Cu Kα** (λ = 1.54178 Å). Cell refinement and data reduction were performed with the **SAIN**T program package. Absorption correction was performed with **SADBAS**. Structures were solved by direct methods using **OLEX 2** (version 1.3) [1]. After obtaining the initial structure for refinement using the standard **SHELXT** program (version 6.1) by using the **OLEX 2** software, the refinement of the main frame should be performed first. After anisotropic refinement of all non-H atoms in the framework, the positions of the **H** atoms were calculated geometrically with riding models. If there is a disorder in the molecule, the **PART** command is used to change the part number/occupancy for the given/selected atom; The **DELU** command restrains the **ADPs** of the atoms in the direction of the bond between them to be equal within the given standard uncertainty; The **SIMU** constraint command was used to make the atomic displacement parameters of selected bonds or atom pairs within the given atoms similar with the given estimated standard deviation. The crystal structure of **F[3]A1-[P(O)Ph]$_3$ @ G15** have completely disordered dichloromethane solvent molecules and the **SQUEEZE** method was used to further refine the data. The crystallographic data reported have been deposited with the **Cambridge Crystallographic Data Centre** (**CCDC**). The graphics of

crystal structures were generated with the programs **Mercury 2020.1 and VMD 1.9.3**. The **B3LYP** [3] density functional method with the **D3**(**BJ**) [4] dispersion correction was employed in this work to carry out all the computations. The **6-31G(d**) [3] basis set was used for the atoms in geometry optimizations using the **PCM** model with dichloroethane as the solvent. Vibrational frequency analyses at the same level of theory were performed to characterize stationary points as local minima without any imaginary frequencies. All **DFT** theoretical calculations have been carried out using the **Gaussian 09** program package.

2. Supplementary Discussion

2.1 Synthesis and Characterization

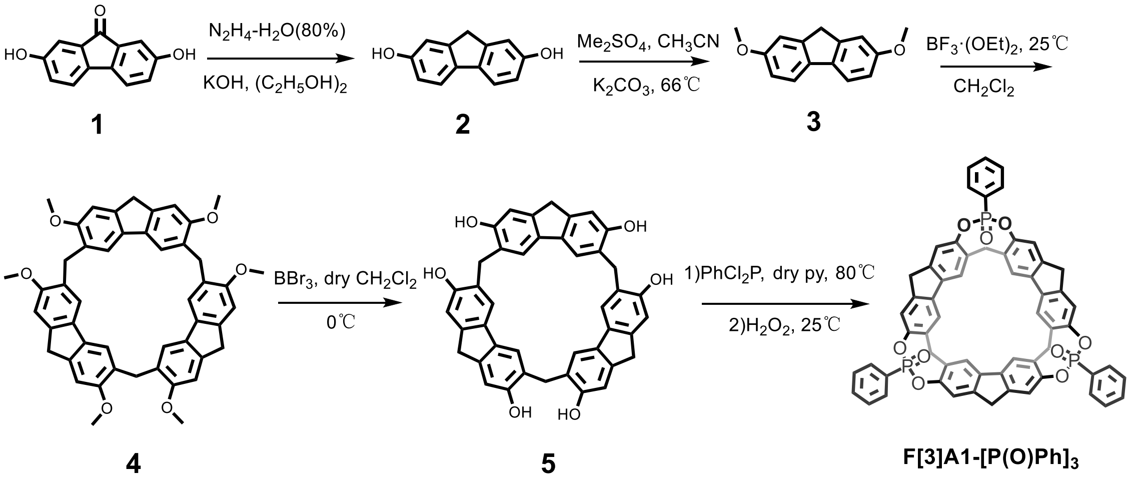

Supplementary Figure 1. Synthesis of **F[3]A1-[P(O)Ph]₃**

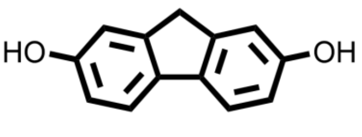

The synthesis of compound **2**:5.4 g (1060 mmol) of 2,7-dihydroxy-9-fluorenone (compound **1**) was added to a 500 mL three-necked flask, followed by the addition of 120 mL (530 mmol) of diethylene glycol as a solvent. After ultrasonication to dissolve the solution, magnets were added for stirring, and 30 mL (250 mmol) of hydrazine hydrate (80%) was slowly introduced. A reflux device was then installed, and the stirring reaction was conducted at 130 ℃ for 8 hours. Following the reaction, a splitter and a water separator were installed. Subsequently, 10.5 g (280 mmol) of solid potassium hydroxide was slowly added, and the mixture was heated to 210 ℃ and stirred for 4 hours. Upon completion of the reaction, the mixture was cooled to room temperature. To neutralize the solution, dilute hydrochloric acid was slowly added under an ice water bath when a large quantity of white solids precipitated. The resulting mixture was filtered, and the filter cake was washed with a saturated sodium chloride solution. The solid obtained after drying weighed 4.72 g, yielding

compound **2** with a 71% yield [2]. [1]**H NMR** (400 **MHz**, **DMSO-***d*6) δ 9.23 (s, 2H), 7.47 (d, J = 8.2 Hz, 2H), 6.89 (d, J = 1.9 Hz, 2H), 6.70 (dd, J = 8.2, 2.3 Hz, 2H), 3.70 (s, 2H). [13]**C NMR** (100 **MHz**, **DMSO-***d*6) δ 156.2 (s), 144.4 (s), 133.4 (s), 119.8 (s), 114.2 (s), 112.6 (s), 36.8 (s).

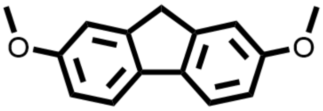

The synthesis of compound **3**:8.7 g (43 mmol) of compound **2** and 45 g (320 mmol) of potassium carbonate were placed in a 1000 mL three-necked flask equipped with a condenser tube and a nitrogen bulb. The three-necked flask had been evacuated and vented. Using a syringe, 360 mL of acetonitrile solvent was added to the flask. Subsequently, 18 g (140 mmol) of dimethyl sulfate was added to the flask using a syringe. The mixture was stirred and refluxed for 24 hours. At the end of the reaction, the mixture was cooled to room temperature. 200 mL of water was added to dissolve the potassium carbonate. Extraction was performed with dichloromethane solvent, and the organic layer was washed several times with saturated sodium chloride solution. The organic layer was then dried with anhydrous sodium sulfate. The solvent was removed by a rotary evaporator, and the product was purified by column chromatography using a mixture of dichloromethane and petroleum ether (1:3 v/v). This process yielded 8.8 g of white solid, representing a 90% yield of compound **3** [2]. [1]**H NMR** (400 **MHz**, **$CDCl_3$**) δ 7.57 (d, J = 8.3 Hz, 2H), 7.07 (s, 2H), 6.90 (d, J = 8.3 Hz, 2H), 3.84 (d, J = 8.7 Hz, 8H). [13]**C NMR** (100 **MHz**, **$CDCl_3$**) δ 157.4 (s), 143.3 (s), 133.5 (s), 118.6 (s), 111.7 (s), 109.6 (s), 54.5 (s), 36.1 (s), 30.4 (s), 29.2 (s).

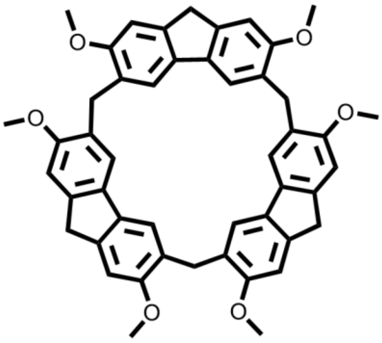

Synthesis of compound **4**: 0.68 g (3 mmol) of compound **3** and 0.5 g (15 mmol) of paraformaldehyde were weighed and added to a 1000 mL reaction flask. 480 mL of dichloromethane solvent was then added to the flask. Then 0.6 mL of boron trifluoride ethyl ether

was added dropwise and the mixture was stirred at room temperature until the color of the solution changed from colorless to dark green. Then 200 mL of saturated sodium bicarbonate solution was added to quench the reaction and the mixture was stirred for another 2 hours. The reaction was then stopped and the organic layer was separated using a liquid separation funnel. The organic layer was collected and washed several times with a saturated sodium chloride solution. After washing, the organic layer was dried with anhydrous sodium sulfate. The solvent was removed using a rotary evaporator and the product was purified by column chromatography using a mixture of dichloromethane and petroleum ether (v/v, 100:1). This process yielded 1.3 g of solid, giving a 60% yield of compound **4** [2]. ^{1}**H NMR** (400 **MHz**, **$CDCl_3$**) δ 7.37 (s, 6H), 7.04 (s, 6H), 4.06 (s, 6H), 3.86 (s, 18H), 3.80 (s, 6H). ^{13}**C NMR** (100 **MHz**, **$CDCl_3$**, 298 K): δ 156.2(s), 142.0(s), 134.7(s), 127.8(s), 119.7(s), 108.1(s), 55.5(s), 36.1(s), 27.5(s).

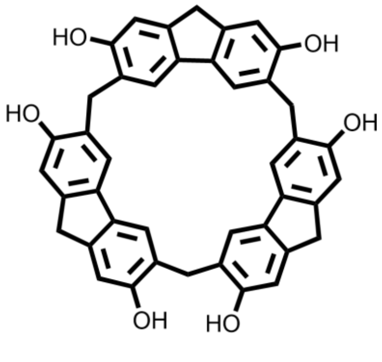

Synthesis of compound **5**: 1.5 g (2.1 mmol) of compound **4** was weighed and placed in a 1000 mL three-necked flask equipped with magnets, a constant pressure-dropping funnel, and a nitrogen bulb. The flask was then evacuated and vented. Using a syringe, 640 mL of dry dichloromethane was added to the three-necked flask and the mixture was stirred in an ice bath. Then, 2.5 mL (33.6 mmol) of boron tribromide was mixed with 40 mL of dry dichloromethane, and the resulting solution was added to the constant-pressure dropping funnel with a syringe. The solution was then slowly dropped into the reaction flask at a controlled rate. The reaction was stopped when the color of the solution changed from light yellow to yellow. Water was added dropwise to quench the boron tribromide and the organic layer was collected. The organic layer was then washed several times with saturated sodium chloride solution and the organic layer was collected again. After washing, the

organic layer was dried with anhydrous sodium sulfate. The solvent was removed by rotary evaporation to give 1.1 g of white solid, which is a 72% yield of compound 5 [2]. ^{1}**H NMR** (400 **MHz**, **DMSO**-*d*6) δ 9.29 (s, 6H), 7.28 (s, 6H), 6.95 (s, 6H), 3.88 (s, 6H), 3.67 (s, 6H). ^{13}**C NMR** (100 **MHz**, **DMSO**-*d*6, 298 K): δ 155.9 (s), 141.9 (s), 133.9 (s), 127.1 (s), 120.2 (s), 109.4 (s), 56.4(s), 36.9 (s), 28.2 (s).

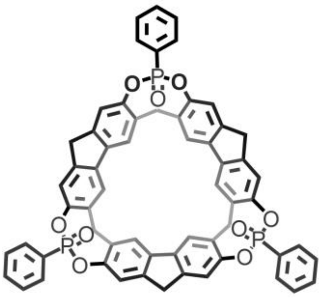

Synthesis of compound **F[3]A1-[P(O)Ph]₃**: 2 g (3.2 mmol) of compound **5** was weighed into a 100 mL three-necked flask. Add 50 mL of pyridine solvent to the flask and dissolve the contents by ultrasonication. While stirring, add 3.1 mL (20.4 mmol) of dichlorophenylphosphine dropwise to the solution. Stir the mixture for 24 hours at 80 ℃. At the end of the reaction, stop stirring, cool the reaction mixture to room temperature, and then add 15 mL of hydrogen peroxide solution dropwise. When the reaction is complete, pour the reaction mixture into water to precipitate the solid product. Filter the solid product and dry it. Purify the product by column chromatography using a solvent mixture of dichloromethane and methanol (v/v, 100:1). This procedure yields 1.6 g of solid product, which is a 51% yield of the compound **F[3]A1-[P(O)Ph]$_3$**. ^{1}**H NMR** (400 **MHz**, **CD_2Cl_2**) δ 8.30 – 8.17 (m, 6H), 7.97 (s, 6H), 7.83 – 7.60 (m, 9H), 7.29 (s, 6H), 4.89 (dd, J = 12.8, 2.7 Hz, 3H), 3.96 (d, J = 12.9 Hz, 3H), 3.82 (d, J = 22.7 Hz, 6H). ^{13}**C NMR** (100 **MHz**, **CD_2Cl_2**) δ 146.8 (d), 144.2 (s), 139.4 (s), 133.6 (d), 131.9 (d), 129.1 (d), 128.0 (s), 125.9 (s), 121.0 (s), 120.1 (s), 36.9 (s), 33.9 (s). ^{31}**P NMR** (162 **MHz**, **CD_2Cl_2**) δ 10.7 (s). **HRMS** (EI): Calcd for $C_{60}H_{39}O_9P_3$ $[M+H]^+$ 997.1880, found 997.1887.

2.2 NMR and HRMS Spectra

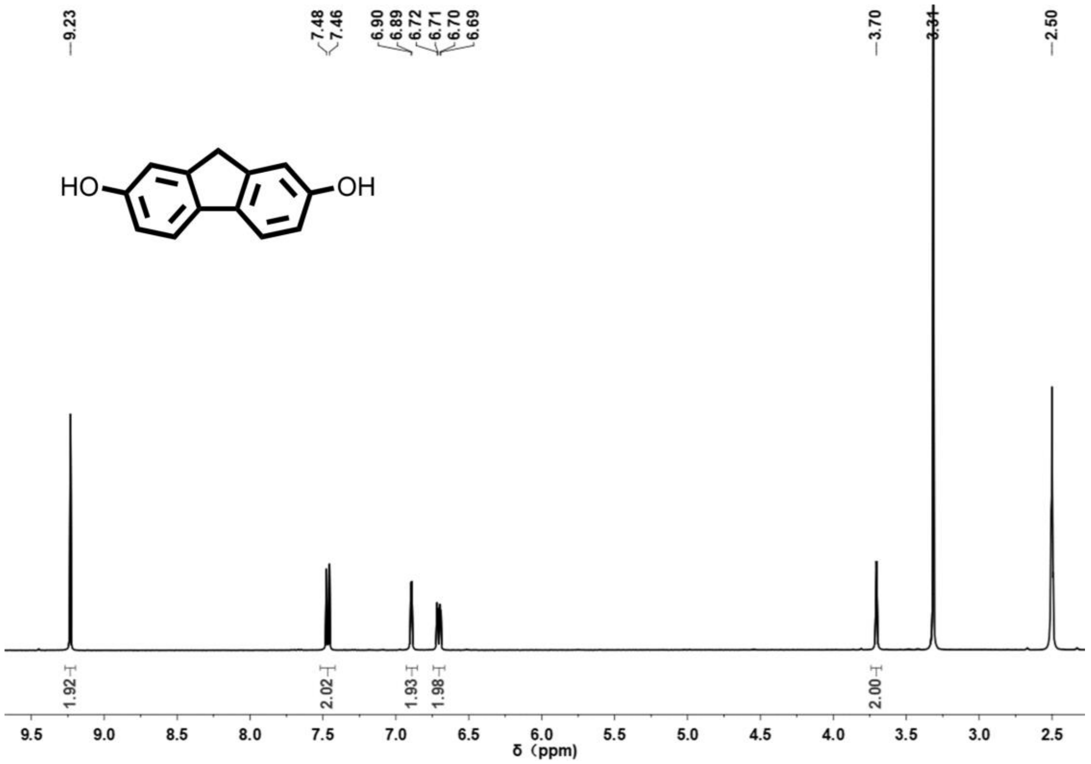

Supplementary Figure 2. **^{1}H-NMR** spectrum of compound **2** (400 **MHz**, **DMSO**-*d*6, 298K).

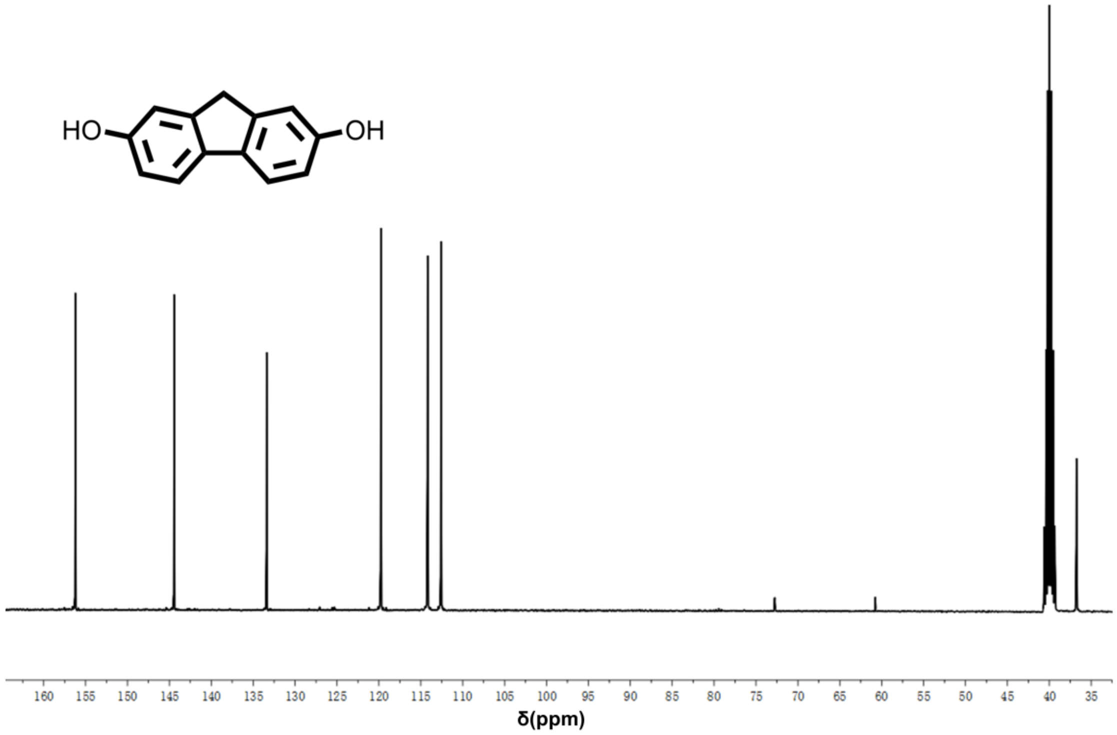

Supplementary Figure 3. ^{13}C-NMR spectrum of compound **2** (100 **MHz**, **DMSO**-*d*6, 298K).

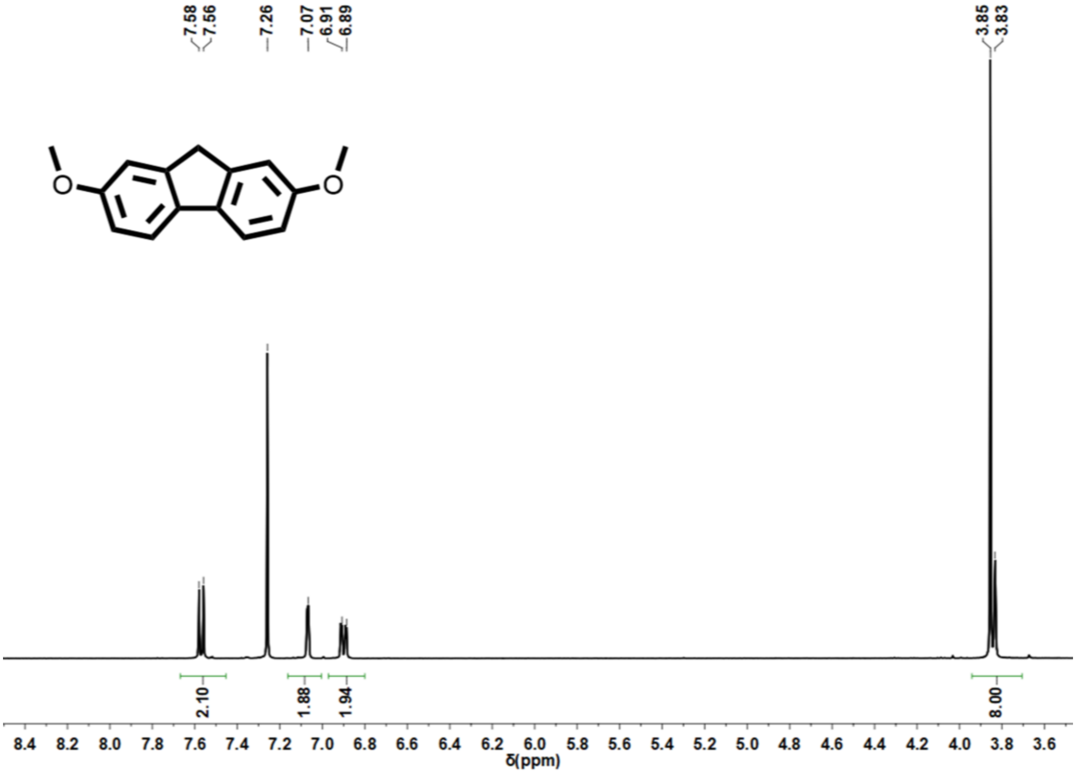

Supplementary Figure 4. [1]**H-NMR** spectrum of compound **3** (400 **MHz**, **$CDCl_3$**, 298K).

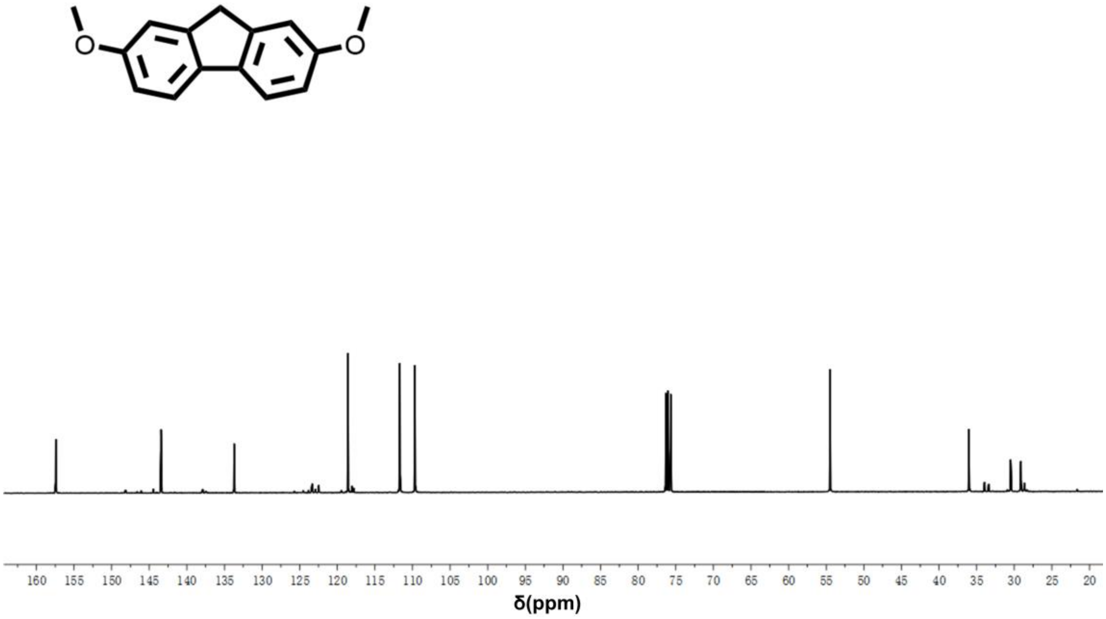

Supplementary Figure 5. ^{13}C-NMR spectrum of compound **3** (100 **MHz**, **$CDCl_3$**, 298K).

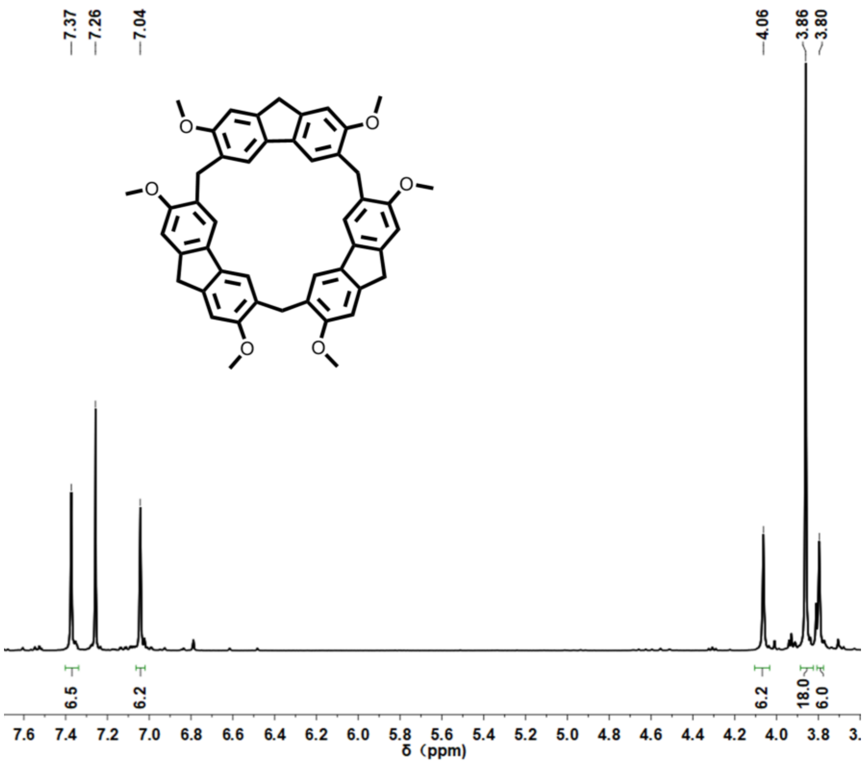

Supplementary Figure 6. ^{1}**H-NMR** spectrum of compound **4** (400 **MHz**, **CDCl₃**, 298K).

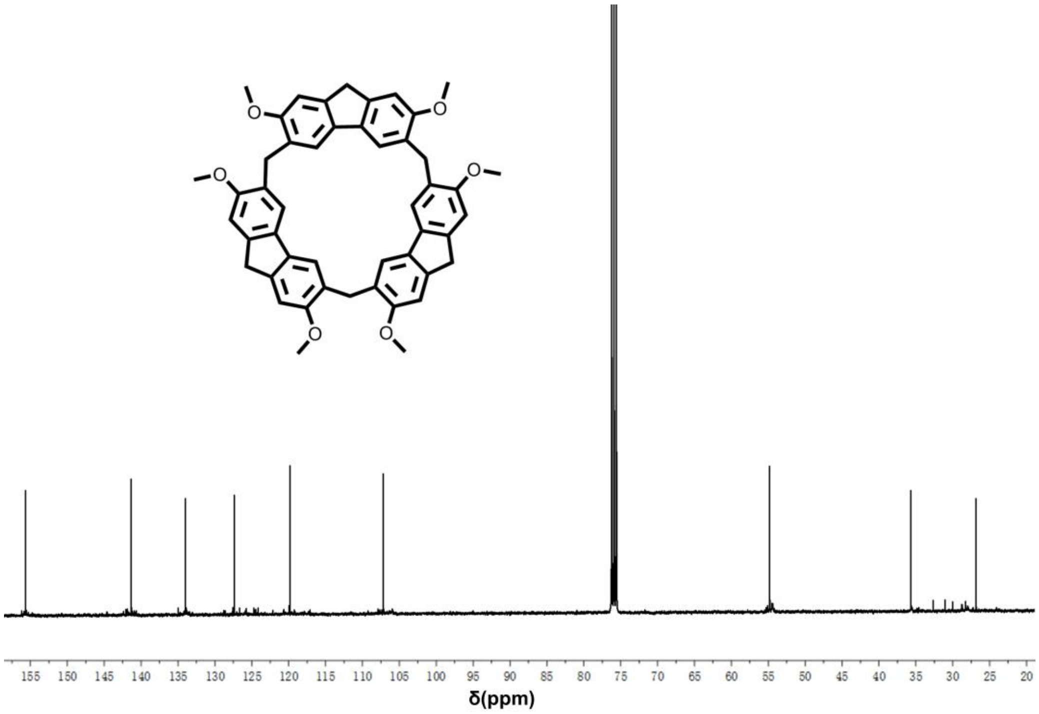

Supplementary Figure 7. ^{13}**C-NMR** spectrum of compound **4** (100 **MHz**, **CDCl₃**, 298K).

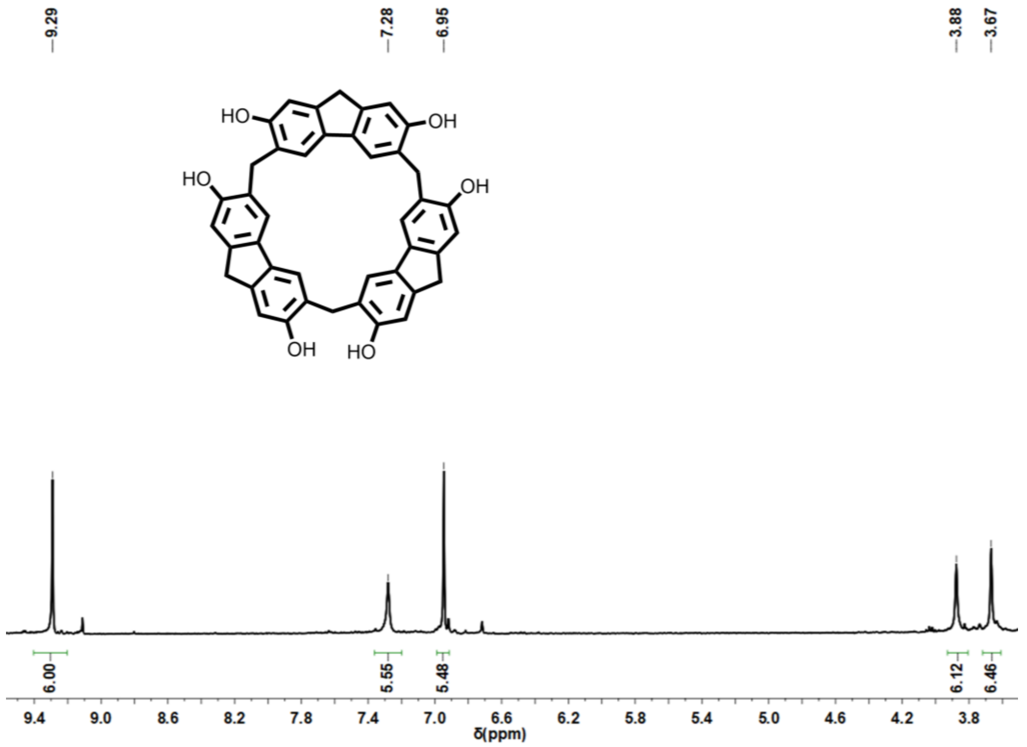

Supplementary Figure 8. ^{1}**H-NMR** spectrum of compound **5** (400 **MHz**, **DMSO**-*d*6, 298K).

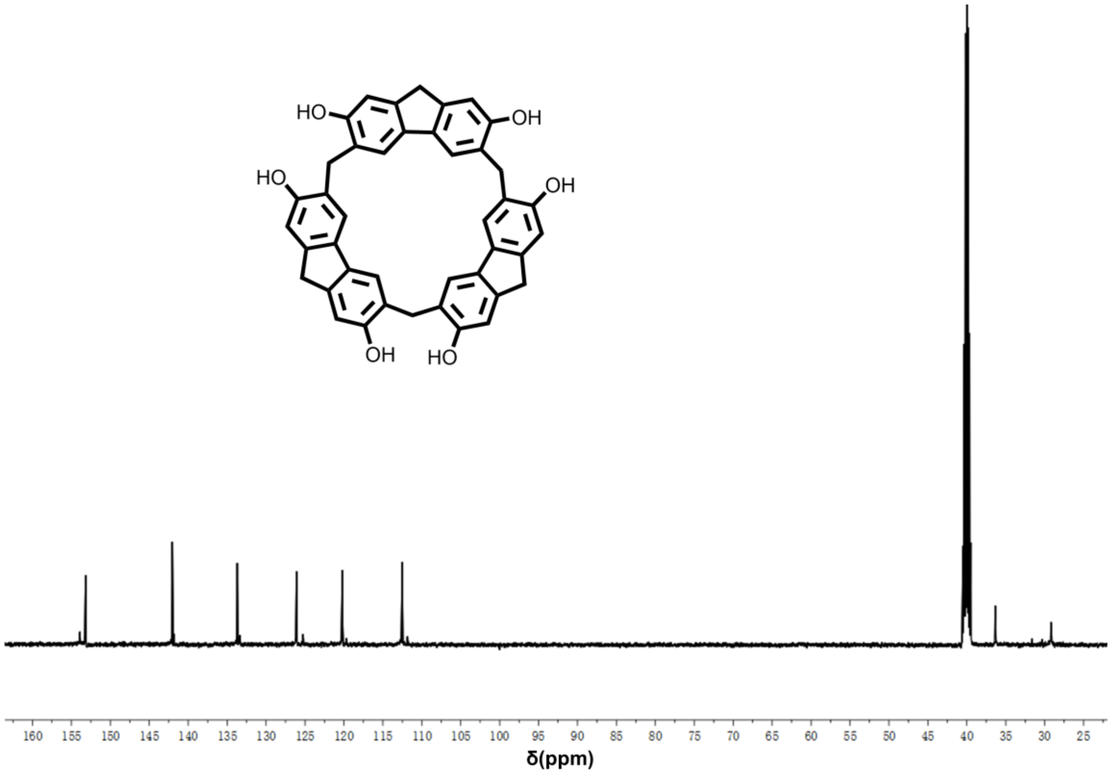

Supplementary Figure 9. ^{13}**C-NMR** spectrum of compound **5** (100 **MHz**, **DMSO**-*d*6, 298K).

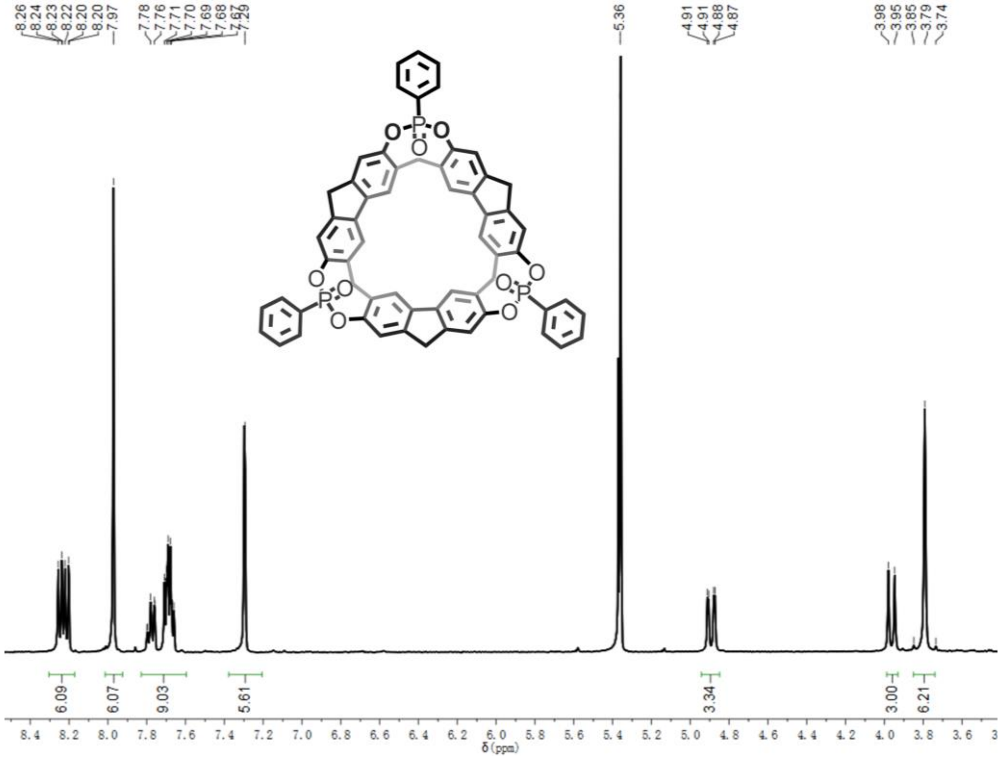

Supplementary Figure 10. ^{1}H-NMR spectrum of **F[3]A1-[P(O)Ph]$_3$** (400 **MHz**, **CD$_2$Cl$_2$**, 298K).

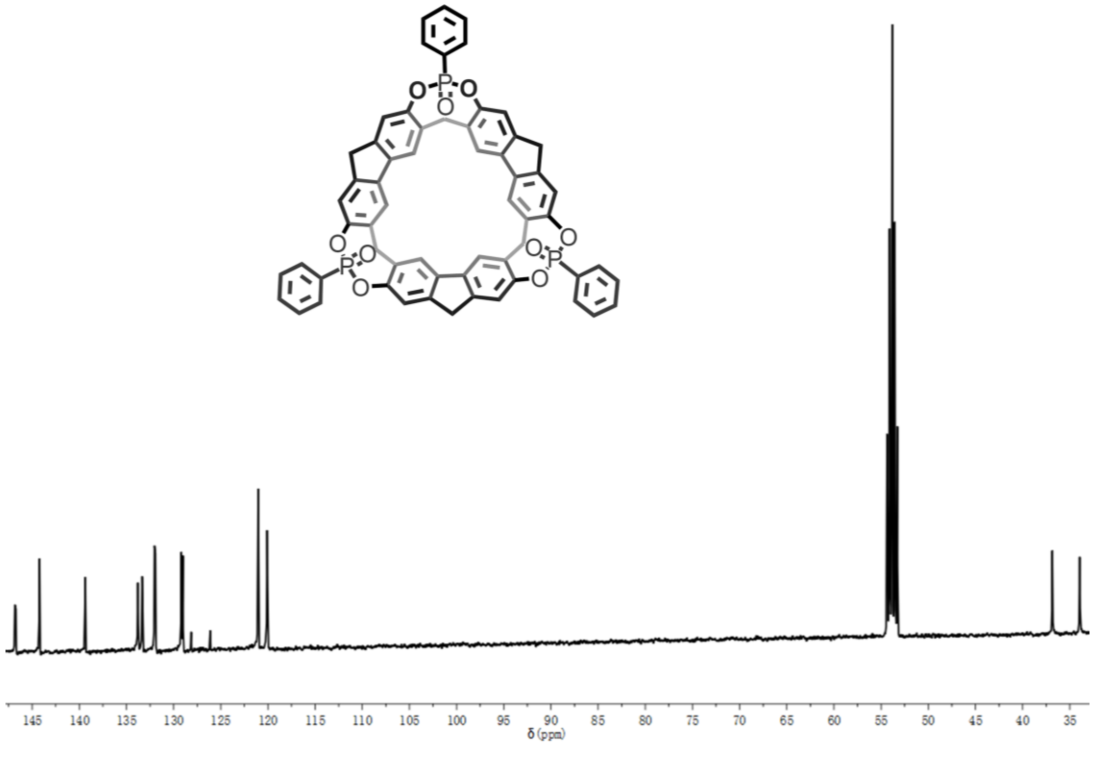

Supplementary Figure 11. ^{13}C-NMR spectrum of **F[3]A1-[P(O)Ph]$_3$** (100 **MHz**, **CDCl$_3$**, 298K).

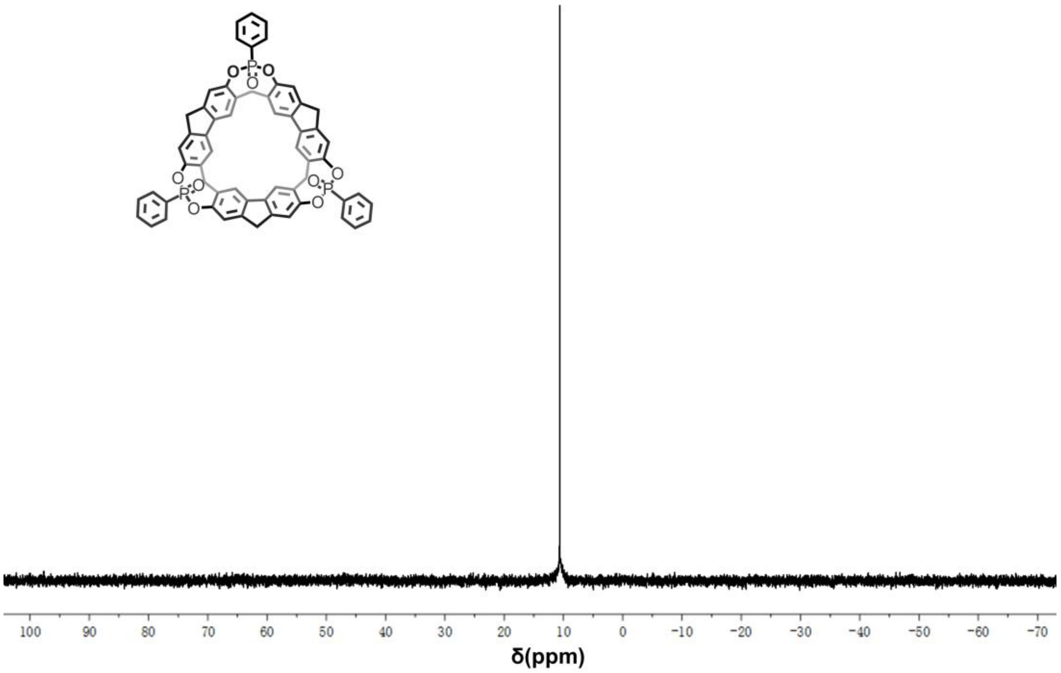

Supplementary Figure 12. ^{31}P-NMR spectrum of **F[3]A1-[P(O)Ph]$_3$** (162 **MHz**, **CD_2Cl_2**, 298K).

2.3 Electrostatic potential surface of F[3]A1-[P(O)Ph]$_3$

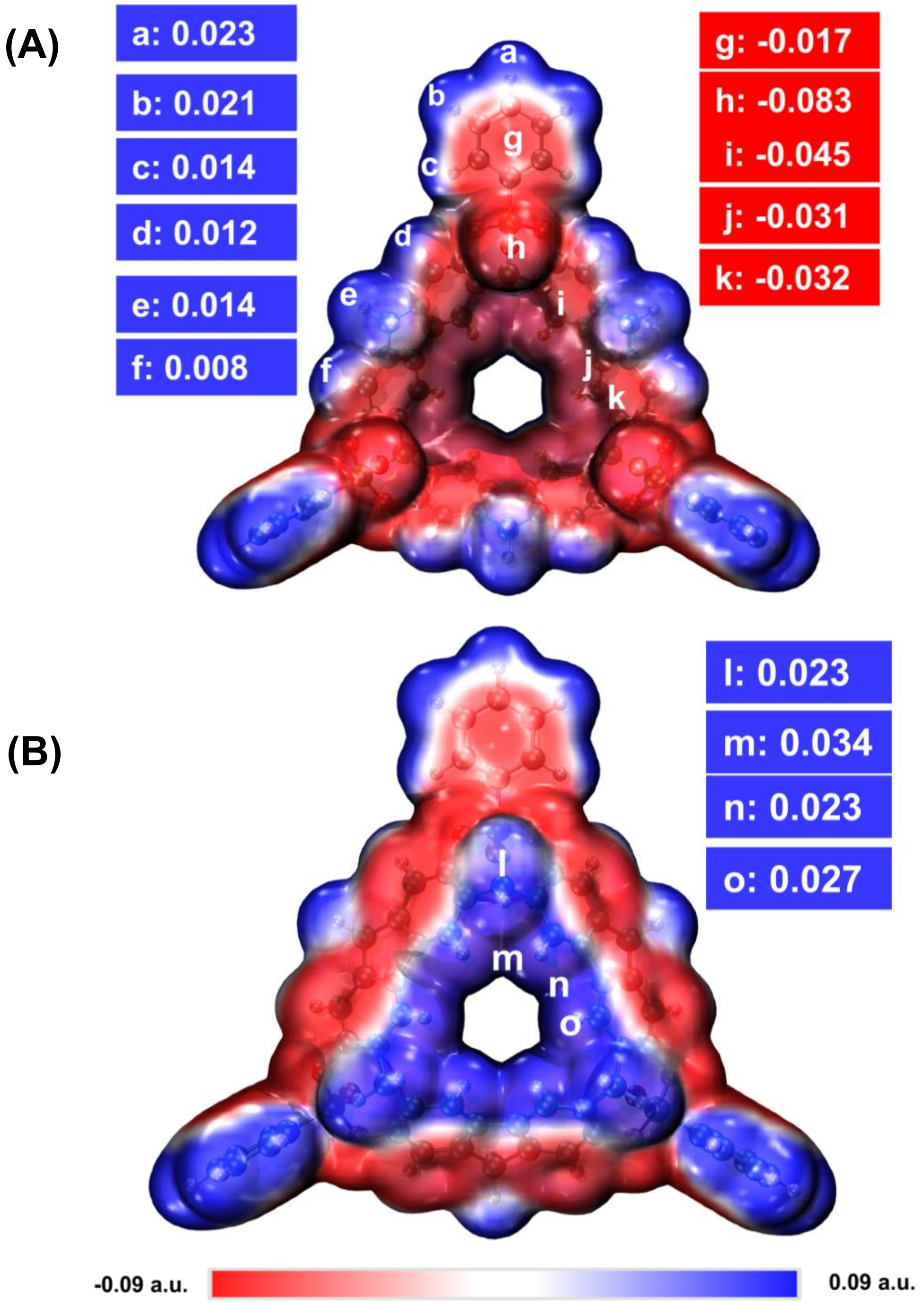

Supplementary Figure 13. The electrostatic potential (ESP) surface of F[3]A1-[P(O)Ph]$_3$. (**A**) View from the front. (**B**) View from the back. Surface **ESP** values (e.V.) are shown. The **RGB** color mode was chosen to clearly show the **ESP** surface. Red represents negatively charged areas blue represents positively charged areas.

2.4. The single crystal structure of dichloromethane @ F[3]A1-[P(O)Ph]$_3$

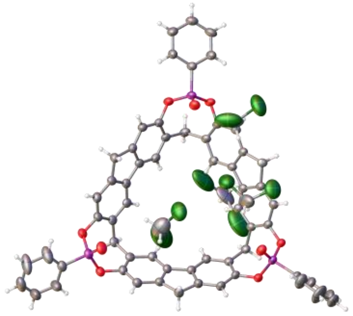

Supplementary Figure 14. X-ray structure of dichloromethane @ **F[3]A1-[P(O)Ph]$_3$**. Thermal ellipsoids were shown at 50% probability level.

CCDC number	2349284
Empirical formula	$C_{62}H_{44}Cl_5O_9P_3$
Formula weight	1204.88
Temperature [K]	193.0
Crystal system	monoclinic
Space group	$P2_1/n$
a [Å]	15.5072(6)
b [Å]	20.8232(8)
c [Å]	18.8755(7)
α [°]	90
β [°]	102.476(2)
γ [°]	90
Volume [Å^3]	5951.2(4)
Z	4
ρ_{calc} [gcm^{-3}]	1.302
μ [mm^{-1}]	3.020
F(000)	2476
Radiation	CuK_α (λ=1.54178 Å)
2θ range [°]	6.40 to 136.62 (0.83 Å)
Index ranges	−18 ≤ h ≤ 18, −21 ≤ k ≤ 24, −18 ≤ l ≤ 22
Reflections collected	62842
Independent reflections	10815, R_{int} = 0.0800, R_{sigma} = 0.0504
Completeness to, θ = 67.679°	99.5 %
Data / Restraints / Parameters	10804/47/759
Absorption correction	0.4099/0.7531
Goodness-of-fit on F^2	1.101
Final *R* indexes, [I≥2σ(I)]	R_1 = 0.0849, wR_2 = 0.2703
Final *R* indexes, [all data]	R_1 = 0.1023, wR_2 = 0.2913
Largest peak/hole [eÅ^{-3}]	1.65/−0.62

The cavity size and depth structure of F[3]A1-[P(O)Ph]$_3$

(A)

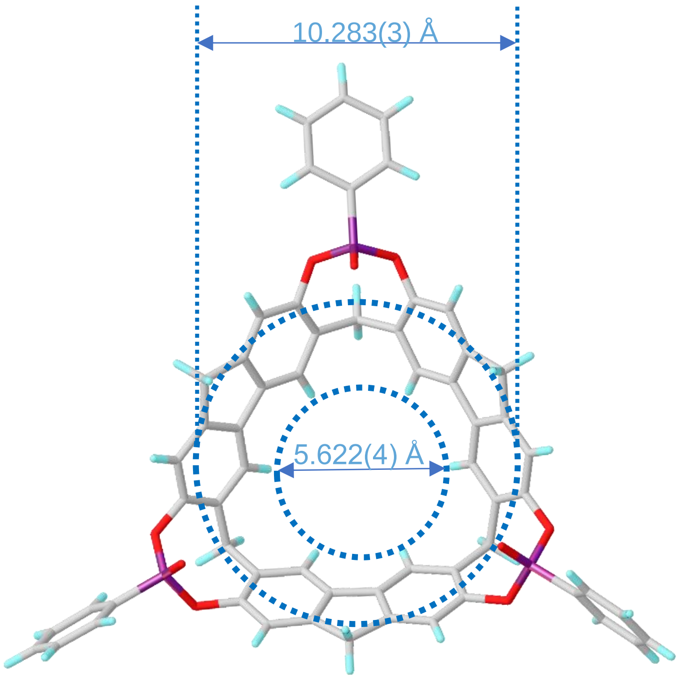

Top view

(B)

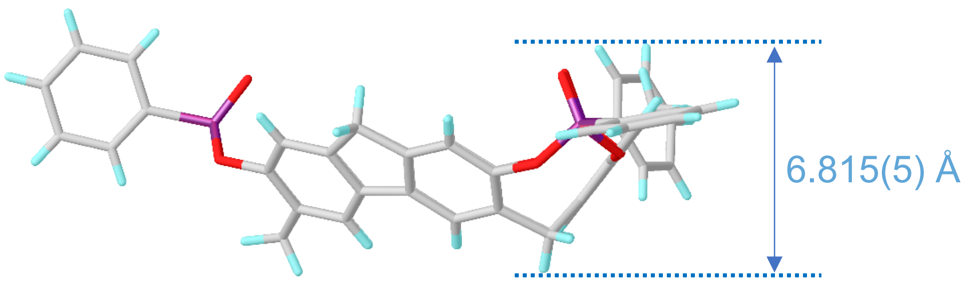

Side view

Supplementary Figure 15. The cavity size and depth structure of F[3]A1-[P(O)Ph]$_3$. Top views (**A**) and side views (**B**) of the single crystal structure of **F[3]A1-[P(O)Ph]$_3$**.

2.5. The single crystal structure of co-crystal

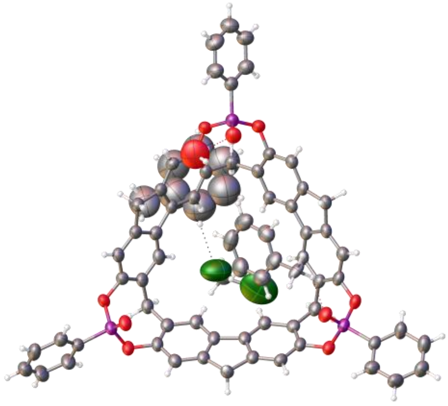

Supplementary Figure 16. X-ray structure of toluene @ **F[3]A1-[P(O)Ph]₃**. Thermal ellipsoids were shown at 50% probability level.

CCDC number	2349293
Empirical formula	$C_{74}H_{58}Cl_2O_{10}P_3$
Formula weight	1223.86
Temperature [K]	193.00
Crystal system	triclinic
Space group	$P\bar{1}$
a [Å]	13.1991(13)
b [Å]	15.5688(16)
c [Å]	15.5888(15)
α [°]	78.707(3)
β [°]	81.110(3)
γ [°]	85.793(3)
Volume [Å3]	3100.6(5)
Z	2
ρcalc [gcm−3]	1.311
μ [mm−1]	0.184
F(000)	1277
Radiation	MoKα (λ=0.71073 Å)
2θ range [°]	4.02 to 55.20 (0.77 Å)
Index ranges	−17 ≤ h ≤ 17, −20 ≤ k ≤ 20, −18 ≤ l ≤ 20
Reflections collected	52924
Independent reflections	14201, Rint = 0.1560, Rsigma = 0.1351
Completeness to,θ = 25.242°	99.9 %
Data / Restraints / Parameters	14201/73/871
Absorption correction	0.6881/0.7446
Goodness-of-fit on F2	1.172
Final R indexes, [I≥2σ(I)]	R1 = 0.1005, wR2 = 0.2111
Final R indexes, [all data]	R1 = 0.2111, wR2 = 0.3372
Largest peak/hole [eÅ−3]	0.86/−0.51

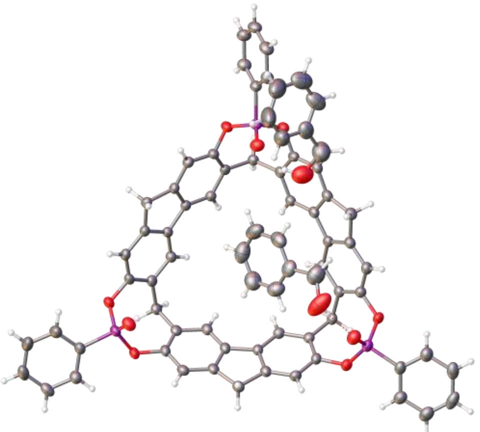

Supplementary Figure 17. X-ray structure of benzyl alcohol @ **F[3]A1-[P(O)Ph]₃**. Thermal ellipsoids were shown at 50% probability level.

CCDC number	2313236
Empirical formula	$C_{74}H_{53}O_{11}P_3$
Formula weight	1211.07
Temperature [K]	193.00
Crystal system	orthorhombic
Space group	$Pbca$
a [Å]	27.0043(8)
b [Å]	15.3204(5)
c [Å]	28.4371(8)
α [°]	90
β [°]	90
γ [°]	90
Volume [Å^3]	11764.9(6)
Z	1
ρ_{calc} [gcm^{-3}]	1.367
μ [mm^{-1}]	1.474
F(000)	5040
Radiation	CuK_α (λ=1.54178 Å)
2θ range [°]	6.22 to 136.65 (0.83 Å)
Index ranges	−32 ≤ h ≤ 32, −14 ≤ k ≤ 17, −34 ≤ l ≤ 34
Reflections collected	123401
Independent reflections	10424, R_{int} = 0.1139, R_{sigma} = 0.0499
Completeness to,θ = 67.679°	97.4 %
Data / Restraints / Parameters	10424/0/795
Absorption correction	0.6720/0.7531
Goodness-of-fit on F^2	1.029
Final *R* indexes, [I≥2σ(I)]	R_1 = 0.0545, wR_2 = 0.1583
Final *R* indexes, [all data]	R_1 = 0.0820, wR_2 = 0.1924
Largest peak/hole [eÅ^{-3}]	0.61/−0.62

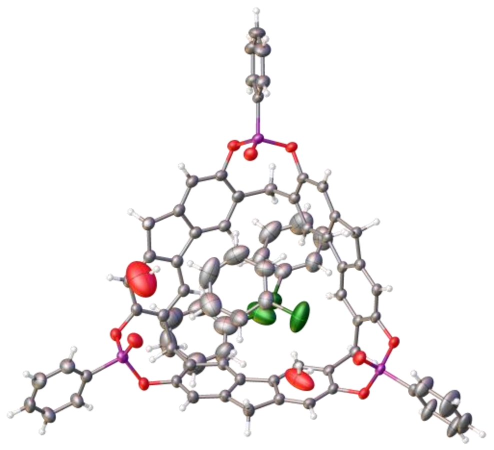

Supplementary Figure 18. X-ray structure of chlorobenzene @ **F[3]A1-[P(O)Ph]$_3$**. Thermal ellipsoids were shown at 50% probability level.

CCDC number	2313220
Empirical formula	$C_{78}H_{57}Cl_3O_{11}P_3$
Formula weight	1361.49
Temperature [K]	193.00
Crystal system	triclinic
Space group	$P\bar{1}$
a [Å]	13.2084(4)
b [Å]	15.9107(5)
c [Å]	16.6898(5)
α [°]	73.207(2)
β [°]	78.313(2)
γ [°]	80.422(2)
Volume [Å^3]	3266.42(18)
Z	2
ρ_{calc} [gcm^{-3}]	1.384
μ [mm^{-1}]	2.486
F(000)	1410
Radiation	CuK_α (λ=1.54178 Å)
2θ range [°]	5.61 to 136.66 (0.83 Å)
Index ranges	−15 ≤ h ≤ 15, −19 ≤ k ≤ 19, −20 ≤ l ≤ 20
Reflections collected	47866
Independent reflections	11903, R_{int} = 0.0419, R_{sigma} = 0.0340
Completeness to,θ = 67.679°	99.6 %
Data / Restraints / Parameters	11903/67/899
Absorption correction	0.6557/0.7531
Goodness-of-fit on F^2	1.075
Final *R* indexes, [I≥2σ(I)]	R_1 = 0.0612, wR_2 = 0.1752
Final *R* indexes, [all data]	R_1 = 0.0716, wR_2 = 0.1813
Largest peak/hole [eÅ^{-3}]	1.30/−1.24

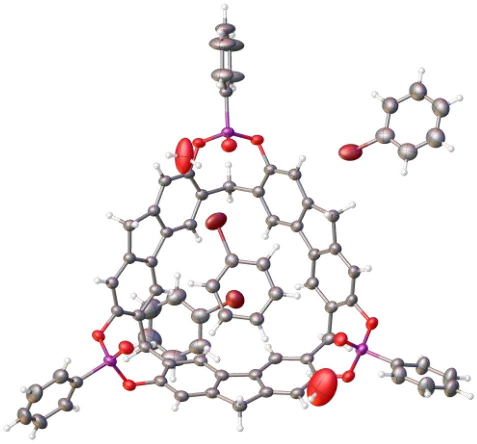

Supplementary Figure 19. X-ray structure of bromobenzene @ **F[3]A1-[P(O)Ph]$_3$**. Thermal ellipsoids were shown at 50% probability level.

CCDC number	2313216
Empirical formula	$C_{76.59}H_{56.83}Br_{2.84}O_{11}P_3$
Formula weight	1472.98
Temperature [K]	193.0
Crystal system	triclinic
Space group	$P\overline{1}$
a [Å]	13.3891(6)
b [Å]	15.8761(7)
c [Å]	16.6069(7)
α [°]	73.859(2)
β [°]	78.400(2)
γ [°]	79.991(2)
Volume [Å^3]	3295.5(3)
Z	2
ρ_{calc} [gcm^{-3}]	1.484
μ [mm^{-1}]	2.272
F(000)	1498
Radiation	GaK_α (λ=1.34139 Å)
2θ range [°]	5.08 to 108.00 (0.83 Å)
Index ranges	−16 ≤ h ≤ 16, −19 ≤ k ≤ 19, −19 ≤ l ≤ 20
Reflections collected	32812
Independent reflections	11872, R_{int} = 0.0422, R_{sigma} = 0.0416
Completeness to,θ = 53.594°	98.4 %
Data / Restraints / Parameters	11879/89/1144
Goodness-of-fit on F^2	1.061
Final *R* indexes, [I≥2σ(I)]	R_1 = 0.0615, wR_2 = 0.1747
Final *R* indexes, [all data]	R_1 = 0.0668, wR_2 = 0.1797
Largest peak/hole [eÅ^{-3}]	1.36/−1.10

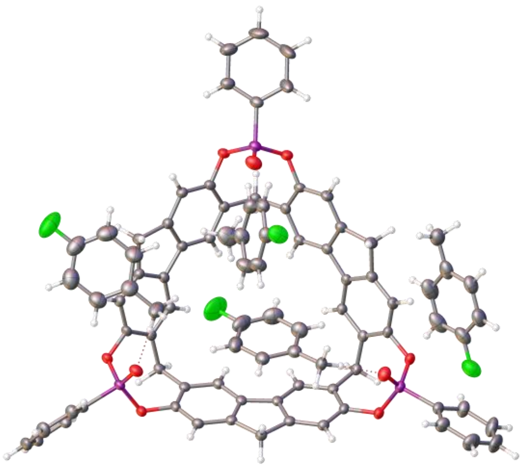

Supplementary Figure 20. X-ray structure of p-fluorotoluene @ **F[3]A1-[P(O)Ph]$_3$**. Thermal ellipsoids were shown at 50% probability level.

CCDC number	2313215
Empirical formula	$C_{88}H_{67}F_4O_9P_3$
Formula weight	1437.32
Temperature [K]	193.00
Crystal system	triclinic
Space group	$P\overline{1}$
a [Å]	11.149(5)
b [Å]	17.007(8)
c [Å]	19.920(6)
α [°]	103.755(17)
β [°]	97.84(2)
γ [°]	107.00(3)
Volume [$Å^3$]	3421(3)
Z	2
ρ_{calc} [gcm^{-3}]	1.395
μ [mm^{-1}]	1.422
F(000)	1496
Radiation	CuK_α (λ=1.54178 Å)
2θ range [°]	4.68 to 138.80 (0.82 Å)
Index ranges	−13 ≤ h ≤ 13, −20 ≤ k ≤ 20, −24 ≤ l ≤ 23
Reflections collected	44496
Independent reflections	12633, R_{int} = 0.0545, R_{sigma} = 0.0498
Completeness to,θ = 67.679°	99.4 %
Data / Restraints / Parameters	12633/0/941
Absorption correction	0.5854/0.7531
Goodness-of-fit on F^2	1.026
Final *R* indexes, [$I≥2\sigma(I)$]	R_1 = 0.0466, wR_2 = 0.1287
Final *R* indexes, [all data]	R_1 = 0.0530, wR_2 = 0.1345
Largest peak/hole [$eÅ^{-3}$]	0.54/−0.68

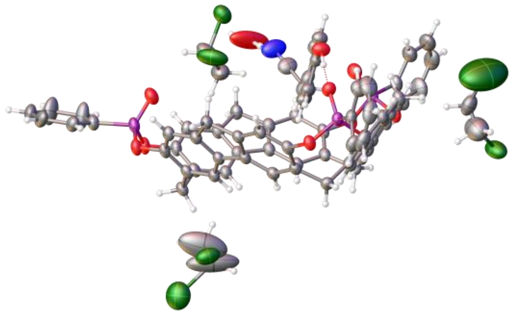

Supplementary Figure 21. X-ray structure of 4-hydroxybenzyl cyanide @ **F[3]A1-[P(O)Ph]$_3$**. Thermal ellipsoids were shown at 50% probability level.

CCDC number	2349281
Empirical formula	$C_{74}H_{58}Cl_6NO_{11}P_3$
Formula weight	1389.57
Temperature [K]	193.00
Crystal system	triclinic
Space group	$P\overline{1}$
a [Å]	9.0654(14)
b [Å]	19.392(3)
c [Å]	19.7896(19)
α [°]	104.980(5)
β [°]	96.223(6)
γ [°]	101.165(6)
Volume [Å^3]	3250.7(7)
Z	2
ρ_{calc} [gcm^{-3}]	1.420
μ [mm^{-1}]	3.274
F(000)	1435
Radiation	CuK_α (λ=1.54178 Å)
2θ range [°]	4.69 to 136.89 (0.83 Å)
Index ranges	−10 ≤ h ≤ 10, −23 ≤ k ≤ 23, −23 ≤ l ≤ 23
Reflections collected	47174
Independent reflections	11848, R_{int} = 0.0413, R_{sigma} = 0.0342
Completeness to,θ = 67.679°	99.4 %
Data / Restraints / Parameters	11848/77/867
Absorption correction	0.5906/0.7531
Goodness-of-fit on F^2	1.070
Final *R* indexes, [I≥2σ(I)]	R_1 = 0.0671, wR_2 = 0.1858
Final *R* indexes, [all data]	R_1 = 0.0745, wR_2 = 0.1926
Largest peak/hole [eÅ^{-3}]	1.31/−1.22

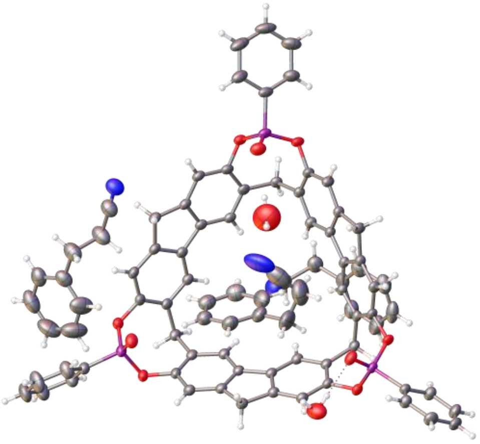

Supplementary Figure 22. X-ray structure of 3-phenylpropanonitrile @ **F[3]A1-[P(O)Ph]₃**. Thermal ellipsoids were shown at 50% probability level.

CCDC number	2313219
Empirical formula	$C_{87}H_{69}N_3O_{11}P_3$
Formula weight	1415.56
Temperature [K]	193.00
Crystal system	triclinic
Space group	$P\overline{1}$
a [Å]	11.6492(3)
b [Å]	18.4686(5)
c [Å]	19.1499(5)
α [°]	62.4920(10)
β [°]	78.5620(10)
γ [°]	89.9150(10)
Volume [Å^3]	3562.35(17)
Z	2
ρ_{calc} [gcm^{-3}]	1.320
μ [mm^{-1}]	1.302
F(000)	1480
Radiation	CuK_α (λ=1.54178 Å)
2θ range [°]	5.34 to 136.54 (0.83 Å)
Index ranges	−14 ≤ h ≤ 14, −22 ≤ k ≤ 22, −23 ≤ l ≤ 22
Reflections collected	47780
Independent reflections	12920, R_{int} = 0.0403, R_{sigma} = 0.0360
Completeness to,θ = 67.679°	99.3 %
Data / Restraints / Parameters	12920/77/940
Absorption correction	0.6062/0.7531
Goodness-of-fit on F^2	1.033
Final *R* indexes, [I≥2σ(I)]	R_1 = 0.0421, wR_2 = 0.1165
Final *R* indexes, [all data]	R_1 = 0.0523, wR_2 = 0.1206
Largest peak/hole [eÅ^{-3}]	0.45/−0.41

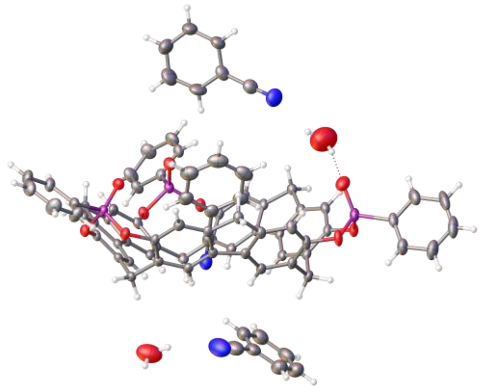

Supplementary Figure 23. X-ray structure of benzonitrile (**G1**) @ **F[3]A1-[P(O)Ph]3**. Thermal ellipsoids were shown at 50% probability level.

CCDC number	2313229
Empirical formula	$C_{77.50}H_{54.50}N_{2.50}O_{10.50}P_3$
Formula weight	1281.64
Temperature [K]	193.00
Crystal system	triclinic
Space group	$P\overline{1}$
a [Å]	13.4085(6)
b [Å]	14.8674(7)
c [Å]	15.4974(7)
α [°]	91.513(2)
β [°]	94.412(2)
γ [°]	91.082(2)
Volume [Å^3]	3078.5(2)
Z	2
ρ_{calc} [gcm^{-3}]	1.383
μ [mm^{-1}]	0.165
F(000)	1332
Radiation	MoK_α (λ=0.71073 Å)
2θ range [°]	4.14 to 54.94 (0.77 Å)
Index ranges	−17 ≤ h ≤ 17, −19 ≤ k ≤ 19, −20 ≤ l ≤ 20
Reflections collected	65469
Independent reflections	13827, R_{int} = 0.0705, R_{sigma} = 0.0514
Completeness to,θ = 25.242°	97.7 %
Data / Restraints / Parameters	13827/53/885
Goodness-of-fit on F^2	1.052
Final *R* indexes, [I≥2σ(I)]	R_1 = 0.0503, wR_2 = 0.1288
Final *R* indexes, [all data]	R_1 = 0.0673, wR_2 = 0.1406
Largest peak/hole [eÅ^{-3}]	0.50/−0.48

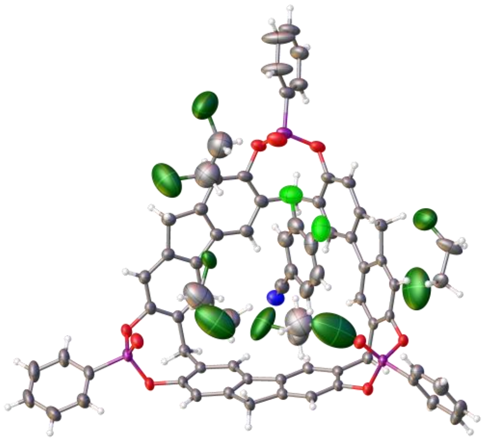

Supplementary Figure 24. X-ray structure of 3,4-difluorobenzonitrile (**G2**) @ **F[3]A1-[P(O)Ph]$_3$**. Thermal ellipsoids were shown at 50% probability level.

CCDC number	2349291
Empirical formula	$C_{77}H_{62}Cl_{10}F_2NO_9P_3$
Formula weight	1630.68
Temperature [K]	193.00
Crystal system	triclinic
Space group	$P\overline{1}$
a [Å]	11.5275(6)
b [Å]	17.4548(8)
c [Å]	18.7651(9)
α [°]	93.069(2)
β [°]	90.362(3)
γ [°]	98.266(3)
Volume [Å^3]	3730.8(3)
Z	2
ρ_{calc} [gcm^{-3}]	1.452
μ [mm^{-1}]	4.552
F(000)	1672
Radiation	CuK_α (λ=1.54178 Å)
2θ range [°]	4.72 to 136.98 (0.83 Å)
Index ranges	−13 ≤ h ≤ 13, −20 ≤ k ≤ 21, −21 ≤ l ≤ 22
Reflections collected	70310
Independent reflections	13608, R_{int} = 0.0557, R_{sigma} = 0.0418
Completeness to,θ = 67.679°	99.5 %
Data / Restraints / Parameters	13614/87/1136
Absorption correction	0.3432/0.7531
Goodness-of-fit on F^2	1.033
Final *R* indexes, [*I*≥2σ(*I*)]	R_1 = 0.0758, wR_2 = 0.2158
Final *R* indexes, [all data]	R_1 = 0.0873, wR_2 = 0.2229
Largest peak/hole [eÅ^{-3}]	0.90/−0.77

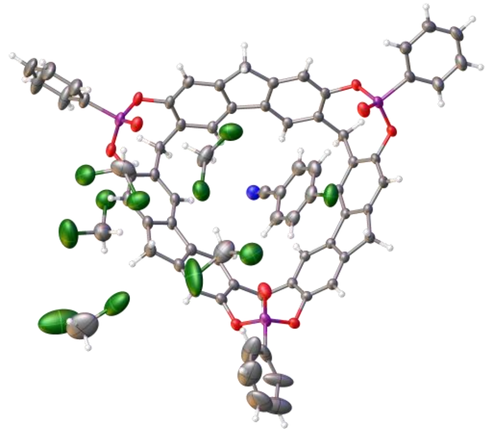

Supplementary Figure 25. X-ray structure of 4-chlorobenzonitrile (**G3**) @ **F[3]A1-[P(O)Ph]$_3$**. Thermal ellipsoids were shown at 50% probability level.

CCDC number	2349282
Empirical formula	$C_{72}H_{53}Cl_{11}NO_9P_3$
Formula weight	1559.01
Temperature [K]	193.00
Crystal system	triclinic
Space group	$P\overline{1}$
a [Å]	13.5435(6)
b [Å]	16.6225(7)
c [Å]	17.1321(7)
α [°]	86.347(2)
β [°]	77.116(2)
γ [°]	68.759(2)
Volume [Å^3]	3503.8(3)
Z	2
ρ_{calc} [gcm^{-3}]	1.478
μ [mm^{-1}]	5.120
F(000)	1592
Radiation	CuK_α (λ=1.54178 Å)
2θ range [°]	5.29 to 137.01 (0.83 Å)
Index ranges	−16 ≤ h ≤ 16, −19 ≤ k ≤ 20, −20 ≤ l ≤ 20
Reflections collected	52112
Independent reflections	12809, R_{int} = 0.0552, R_{sigma} = 0.0465
Completeness to,θ = 67.679°	99.7 %
Data / Restraints / Parameters	12812/131/865
Absorption correction	0.3477/0.7531
Goodness-of-fit on F^2	1.069
Final *R* indexes, [I≥2σ(I)]	R_1 = 0.0689, wR_2 = 0.1923
Final *R* indexes, [all data]	R_1 = 0.0838, wR_2 = 0.2015
Largest peak/hole [eÅ^{-3}]	1.57/−0.99

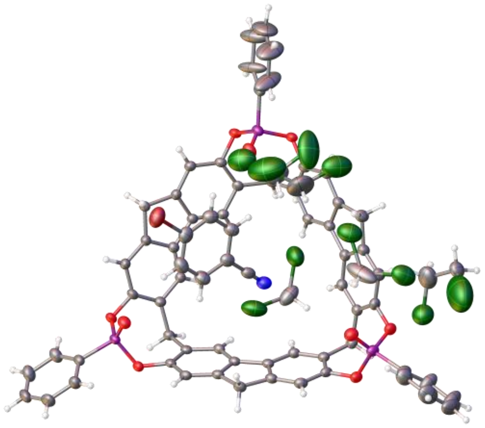

Supplementary Figure 26. X-ray structure of 4-bromobenzonitrile (**G4**) @ **F[3]A1-[P(O)Ph]$_3$**. Thermal ellipsoids were shown at 50% probability level.

CCDC number	2349283
Empirical formula	$C_{73}H_{55}BrCl_{10}NO_9P_3$
Formula weight	1617.50
Temperature [K]	193.00
Crystal system	triclinic
Space group	$P\overline{1}$
a [Å]	13.5592(5)
b [Å]	16.6761(6)
c [Å]	17.1540(7)
α [°]	85.766(2)
β [°]	77.294(2)
γ [°]	69.018(2)
Volume [Å^3]	3532.8(2)
Z	2
ρ_{calc} [gcm^{-3}]	1.521
μ [mm^{-1}]	5.398
F(000)	1644
Radiation	CuK_α (λ=1.54178 Å)
2θ range [°]	5.28 to 136.74 (0.83 Å)
Index ranges	−16 ≤ h ≤ 16, −20 ≤ k ≤ 20, −20 ≤ l ≤ 20
Reflections collected	54022
Independent reflections	12905, R_{int} = 0.0771, R_{sigma} = 0.0731
Completeness to,θ = 67.679°	99.8 %
Data / Restraints / Parameters	12905/24/874
Absorption correction	0.4152/0.7531
Goodness-of-fit on F^2	1.082
Final *R* indexes, [I≥2σ(I)]	R_1 = 0.0724, wR_2 = 0.1967
Final *R* indexes, [all data]	R_1 = 0.1033, wR_2 = 0.2103
Largest peak/hole [eÅ^{-3}]	1.31/−1.59

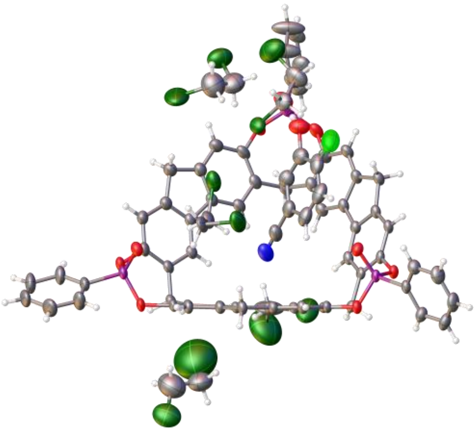

Supplementary Figure 27. X-ray structure of 4-fluoro-3-hydroxybenzonitrile (**G5**) @ **F[3]A1-[P(O)Ph]$_3$**. Thermal ellipsoids were shown at 50% probability level.

CCDC number	2349290
Empirical formula	$C_{153}H_{124}Cl_{20}F_2N_2O_{20}P_6$
Formula weight	3243.35
Temperature [K]	193.00
Crystal system	triclinic
Space group	$P\overline{1}$
a [Å]	11.5275(6)
b [Å]	17.4548(8)
c [Å]	18.7651(9)
α [°]	93.069(2)
β [°]	90.362(3)
γ [°]	98.266(3)
Volume [Å^3]	3730.8(3)
Z	2
ρ_{calc} [gcm^{-3}]	1.444
μ [mm^{-1}]	4.539
F(000)	1664
Radiation	CuK_α (λ=1.54178 Å)
2θ range [°]	4.72 to 136.98 (0.83 Å)
Index ranges	−13 ≤ h ≤ 13, −21 ≤ k ≤ 21, −21 ≤ l ≤ 22
Reflections collected	63240
Independent reflections	13535, R_{int} = 0.0433, R_{sigma} = 0.0361
Completeness to,θ = 67.679°	98.9 %
Data / Restraints / Parameters	13535/115/1118
Absorption correction	0.3465/0.7531
Goodness-of-fit on F^2	1.019
Final *R* indexes, [I≥2σ(I)]	R_1 = 0.0807, wR_2 = 0.2302
Final *R* indexes, [all data]	R_1 = 0.0861, wR_2 = 0.2357
Largest peak/hole [eÅ^{-3}]	0.93/−0.60

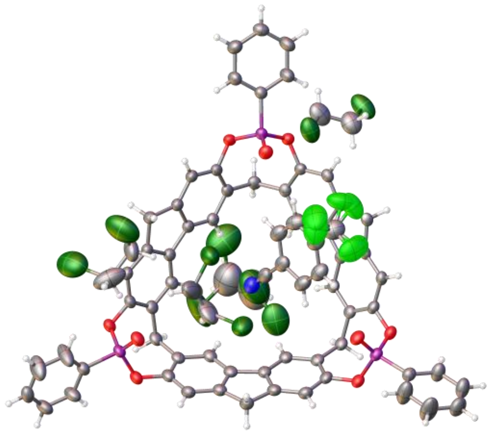

Supplementary Figure 28. X-ray structure of 4-trifluoromethylbenzonitrile (**G6**) @ **F[3]A1-[P(O)Ph]₃**. Thermal ellipsoids were shown at 50% probability level.

CCDC number	2349280
Empirical formula	$C_{76}H_{55}Cl_8F_3NO_9P_3$
Formula weight	1559.72
Temperature [K]	193.00
Crystal system	triclinic
Space group	$P\overline{1}$
a [Å]	12.0737(6)
b [Å]	13.8392(7)
c [Å]	22.7708(11)
α [°]	77.510(2)
β [°]	83.098(2)
γ [°]	74.387(3)
Volume [Å^3]	3569.9(3)
Z	2
ρ_{calc} [gcm^{-3}]	1.451
μ [mm^{-1}]	4.080
F(000)	1596
Radiation	CuK_α (λ=1.54178 Å)
2θ range [°]	6.76 to 136.77 (0.83 Å)
Index ranges	−14 ≤ h ≤ 14, −16 ≤ k ≤ 16, −27 ≤ l ≤ 27
Reflections collected	49110
Independent reflections	12944, R_{int} = 0.0561, R_{sigma} = 0.0504
Completeness to,θ = 67.679°	99.1 %
Data / Restraints / Parameters	12944/80/947
Absorption correction	0.3875/0.7531
Goodness-of-fit on F^2	1.070
Final *R* indexes, [*I*≥2σ(*I*)]	R_1 = 0.0835, wR_2 = 0.2490
Final *R* indexes, [all data]	R_1 = 0.1010, wR_2 = 0.2630
Largest peak/hole [eÅ^{-3}]	1.49/−1.36

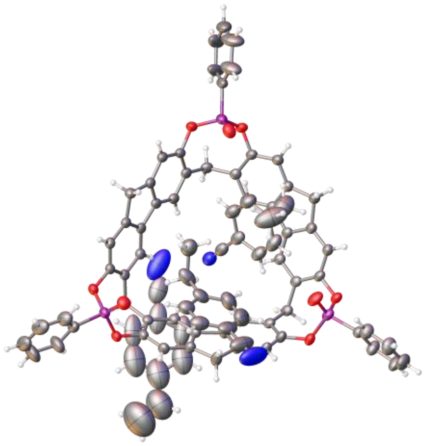

Supplementary Figure 29. X-ray structure of 4-ethylbenzonitrile (**G7**) @ **F[3]A1-[P(O)Ph]$_3$**. Thermal ellipsoids were shown at 50% probability level.

CCDC number	2349292
Empirical formula	$C_{87}H_{66}N_3O_9P_3$
Formula weight	1390.33
Temperature [K]	193.00
Crystal system	monoclinic
Space group	$P2_1/c$
a [Å]	21.0493(4)
b [Å]	17.7850(4)
c [Å]	20.8514(5)
α [°]	90
β [°]	115.9060(10)
γ [°]	90
Volume [Å^3]	7021.6(3)
Z	4
ρ_{calc} [gcm^{-3}]	1.315
μ [mm^{-1}]	1.297
F(000)	2904
Radiation	CuK_α (λ=1.54178 Å)
2θ range [°]	4.67 to 136.61 (0.83 Å)
Index ranges	−25 ≤ h ≤ 23, −21 ≤ k ≤ 21, −25 ≤ l ≤ 25
Reflections collected	115813
Independent reflections	12853, R_{int} = 0.0598, R_{sigma} = 0.0255
Completeness to,θ = 67.679°	100.0 %
Data / Restraints / Parameters	12853/123/921
Absorption correction	0.6283/0.7531
Goodness-of-fit on F^2	1.076
Final *R* indexes, [*I*≥2σ(*I*)]	R_1 = 0.0567, wR_2 = 0.1618
Final *R* indexes, [all data]	R_1 = 0.0643, wR_2 = 0.1681
Largest peak/hole [eÅ^{-3}]	0.82/−0.72

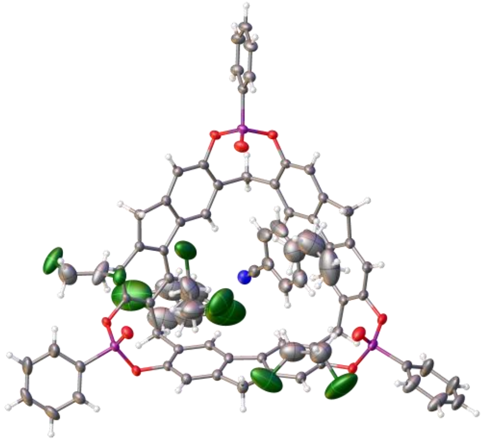

Supplementary Figure 30. X-ray structure of 4-butylbenzonitrile (**G8**) @ **F[3]A1-[P(O)Ph]₃**. Thermal ellipsoids were shown at 50% probability level.

CCDC number	2313234
Empirical formula	$C_{78}H_{66}Cl_7NO_9P_3$
Formula weight	1501.83
Temperature [K]	193.00
Crystal system	triclinic
Space group	$P\overline{1}$
a [Å]	11.2207(5)
b [Å]	17.5286(8)
c [Å]	18.6273(9)
α [°]	90.740(2)
β [°]	93.467(2)
γ [°]	97.961(2)
Volume [Å^3]	3620.9(3)
Z	2
ρ_{calc} [gcm^{-3}]	1.377
μ [mm^{-1}]	3.606
F(000)	1553
Radiation	CuK_α (λ=1.54178 Å)
2θ range [°]	4.75 to 136.69 (0.83 Å)
Index ranges	−13 ≤ h ≤ 13, −21 ≤ k ≤ 21, −22 ≤ l ≤ 22
Reflections collected	61059
Independent reflections	13180, R_{int} = 0.0424, R_{sigma} = 0.0342
Completeness to,θ = 67.679°	99.5 %
Data / Restraints / Parameters	13180/50/921
Absorption correction	0.5348/0.7531
Goodness-of-fit on F^2	1.027
Final *R* indexes, [I≥2σ(I)]	R_1 = 0.0631, wR_2 = 0.1672
Final *R* indexes, [all data]	R_1 = 0.0687, wR_2 = 0.1703
Largest peak/hole [eÅ^{-3}]	0.81/−1.03

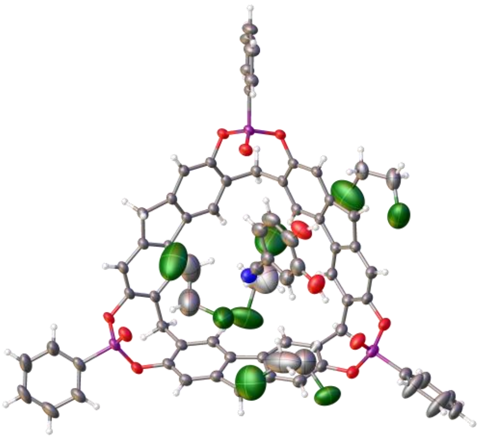

Supplementary Figure 31. X-ray structure of 3,4-dihydroxybenzonitrile (**G9**) @ **F[3]A1-[P(O)Ph]₃**. Thermal ellipsoids were shown at 50% probability level.

CCDC number	2349288
Empirical formula	$C_{77}H_{63}Cl_{10}NO_{11}P_3$
Formula weight	1625.69
Temperature [K]	193.00
Crystal system	triclinic
Space group	$P\overline{1}$
a [Å]	11.5281(6)
b [Å]	17.4546(9)
c [Å]	18.7652(9)
α [°]	93.073(2)
β [°]	90.363(3)
γ [°]	98.274(2)
Volume [Å^3]	3730.8(3)
Z	2
ρ_{calc} [gcm^{-3}]	1.447
μ [mm^{-1}]	4.531
F(000)	1670
Radiation	CuK_α (λ=1.54184 Å)
2θ range [°]	4.72 to 136.91 (0.83 Å)
Index ranges	−13 ≤ h ≤ 13, −20 ≤ k ≤ 21, −21 ≤ l ≤ 22
Reflections collected	68032
Independent reflections	13532, R_{int} = 0.0432, R_{sigma} = 0.0354
Completeness to,θ = 67.684°	99.1 %
Data / Restraints / Parameters	13532/63/1126
Absorption correction	0.3411/0.7531
Goodness-of-fit on F^2	1.033
Final *R* indexes, [I≥2σ(I)]	R_1 = 0.0894, wR_2 = 0.2553
Final *R* indexes, [all data]	R_1 = 0.0945, wR_2 = 0.2606
Largest peak/hole [eÅ^{-3}]	1.10/−1.31

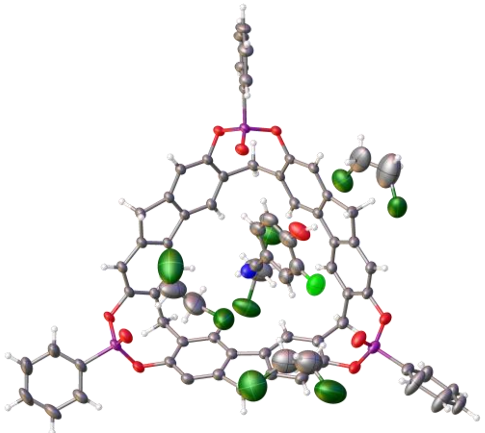

Supplementary Figure 32. X-ray structure of 3-fluoro-4-hydroxybenzonitrile (**G10**) @ **F[3]A1-[P(O)Ph]$_3$**. Thermal ellipsoids were shown at 50% probability level.

CCDC number	2313225
Empirical formula	$C_{76}H_{60}Cl_9FNO_{10}P_3$
Formula weight	1578.21
Temperature [K]	193.00
Crystal system	triclinic
Space group	$P\bar{1}$
a [Å]	11.5285(6)
b [Å]	17.4555(8)
c [Å]	18.7642(9)
α [°]	93.070(2)
β [°]	90.350(3)
γ [°]	98.285(3)
Volume [Å^3]	3730.9(3)
Z	2
ρ_{calc} [gcm^{-3}]	1.405
μ [mm^{-1}]	4.202
F(000)	1620
Radiation	CuK_α (λ=1.54184 Å)
2θ range [°]	4.72 to 137.91 (0.83 Å)
Index ranges	−13 ≤ h ≤ 13, −21 ≤ k ≤ 21, −21 ≤ l ≤ 22
Reflections collected	58501
Independent reflections	13686, R_{int} = 0.0436, R_{sigma} = 0.0384
Completeness to,θ = 67.684°	98.8 %
Data / Restraints / Parameters	13686/65/1164
Absorption correction	0.3366/0.7533
Goodness-of-fit on F^2	1.037
Final *R* indexes, [*I*≥2σ(*I*)]	R_1 = 0.0892, wR_2 = 0.2679
Final *R* indexes, [all data]	R_1 = 0.0948, wR_2 = 0.2737
Largest peak/hole [eÅ^{-3}]	1.25/−1.27

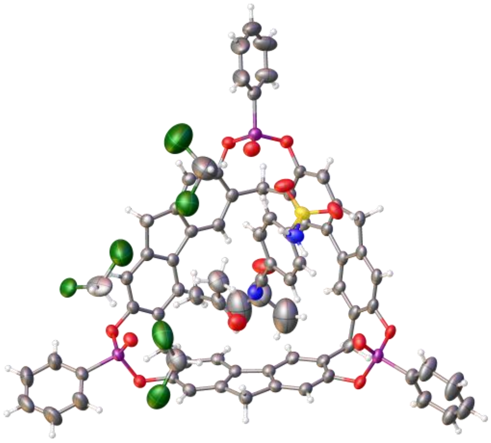

Supplementary Figure 33. X-ray structure of 4-cyanobenzenesulfonamide (**G11**) @ **F[3]A1-[P(O)Ph]$_3$**. Thermal ellipsoids were shown at 50% probability level.

CCDC number	2349285
Empirical formula	$C_{150}H_{122}Cl_{14}N_4O_{25}P_6S_2$
Formula weight	3126.75
Temperature [K]	193.0
Crystal system	triclinic
Space group	$P\overline{1}$
a [Å]	13.8952(8)
b [Å]	15.9908(8)
c [Å]	18.9799(10)
α [°]	110.369(4)
β [°]	99.958(4)
γ [°]	107.429(3)
Volume [Å^3]	3586.3(4)
Z	2
ρ_{calc} [gcm^{-3}]	1.448
μ [mm^{-1}]	3.972
F(000)	1610
Radiation	CuK_α (λ=1.54178 Å)
2θ range [°]	5.22 to 137.04 (0.83 Å)
Index ranges	−16 ≤ h ≤ 16, −19 ≤ k ≤ 19, −22 ≤ l ≤ 22
Reflections collected	61166
Independent reflections	12835, R_{int} = 0.1023, R_{sigma} = 0.0879
Completeness to,θ = 67.679°	97.6 %
Data / Restraints / Parameters	12835/87/1172
Absorption correction	0.6538/0.7531
Goodness-of-fit on F^2	1.017
Final *R* indexes, [*I*≥2σ(*I*)]	R_1 = 0.0664, wR_2 = 0.1827
Final *R* indexes, [all data]	R_1 = 0.1081, wR_2 = 0.2071
Largest peak/hole [eÅ^{-3}]	0.50/−0.68

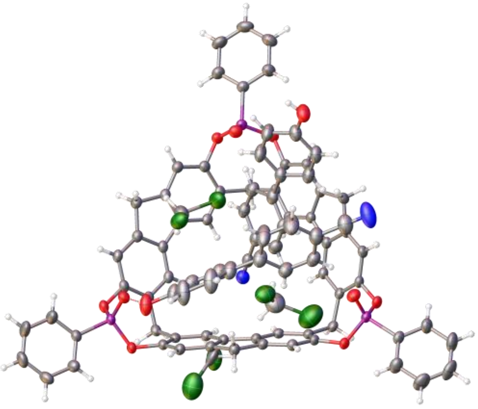

Supplementary Figure 34. X-ray structure of 4'-Hydroxy-4-biphenylcarbonitrile (**G12**) @ **F[3]A1-[P(O)Ph]₃**. Thermal ellipsoids were shown at 50% probability level.

CCDC number	2313221
Empirical formula	$C_{88}H_{61}Cl_4N_2O_{11}P_3$
Formula weight	1557.09
Temperature [K]	193.00
Crystal system	triclinic
Space group	$P\overline{1}$
a [Å]	13.8358(7)
b [Å]	15.8220(8)
c [Å]	19.3804(11)
α [°]	79.497(2)
β [°]	70.001(2)
γ [°]	71.916(2)
Volume [Å^3]	3776.0(4)
Z	2
ρ_{calc} [gcm^{-3}]	1.369
μ [mm^{-1}]	2.555
F(000)	1608
Radiation	CuK_α (λ=1.54178 Å)
2θ range [°]	4.87 to 136.43 (0.83 Å)
Index ranges	−16 ≤ h ≤ 16, −18 ≤ k ≤ 19, −23 ≤ l ≤ 23
Reflections collected	49018
Independent reflections	13708, R_{int} = 0.0443, R_{sigma} = 0.0389
Completeness to,θ = 67.679°	99.4 %
Data / Restraints / Parameters	13708/79/1059
Absorption correction	0.5643/0.7531
Goodness-of-fit on F^2	1.062
Final *R* indexes, [I≥2σ(I)]	R_1 = 0.0458, wR_2 = 0.1286
Final *R* indexes, [all data]	R_1 = 0.0577, wR_2 = 0.1335
Largest peak/hole [eÅ^{-3}]	1.08/−0.55

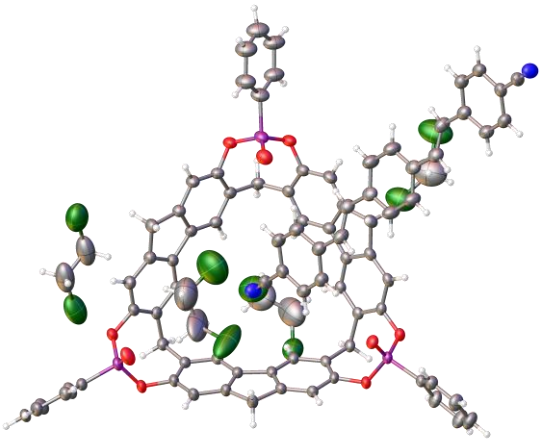

Supplementary Figure 35. X-ray structure of 1,4-bis(4-cyanostyryl)benzene (**G13**) @ **F[3]A1-[P(O)Ph]$_3$**. Thermal ellipsoids were shown at 50% probability level.

CCDC number	2349287
Empirical formula	$C_{157}H_{126}Cl_{14}N_2O_{21}P_6$
Formula weight	3058.71
Temperature [K]	193.00
Crystal system	triclinic
Space group	$P\overline{1}$
a [Å]	13.7107(9)
b [Å]	17.3371(10)
c [Å]	17.3676(10)
α [°]	111.866(3)
β [°]	97.685(4)
γ [°]	99.091(3)
Volume [Å^3]	3698.7(4)
Z	2
ρ_{calc} [gcm^{-3}]	1.373
μ [mm^{-1}]	3.557
F(000)	1578
Radiation	CuK_α (λ=1.54178 Å)
2θ range [°]	5.61 to 137.20 (0.83 Å)
Index ranges	−16 ≤ h ≤ 16, −20 ≤ k ≤ 20, −20 ≤ l ≤ 20
Reflections collected	61162
Independent reflections	13456, R_{int} = 0.0777, R_{sigma} = 0.0642
Completeness to,θ = 67.679°	99.3 %
Data / Restraints / Parameters	13456/86/1040
Absorption correction	0.5227/0.7531
Goodness-of-fit on F^2	1.052
Final *R* indexes, [I≥2σ(I)]	R_1 = 0.0834, wR_2 = 0.2493
Final *R* indexes, [all data]	R_1 = 0.1112, wR_2 = 0.2679
Largest peak/hole [eÅ^{-3}]	1.25/−0.88

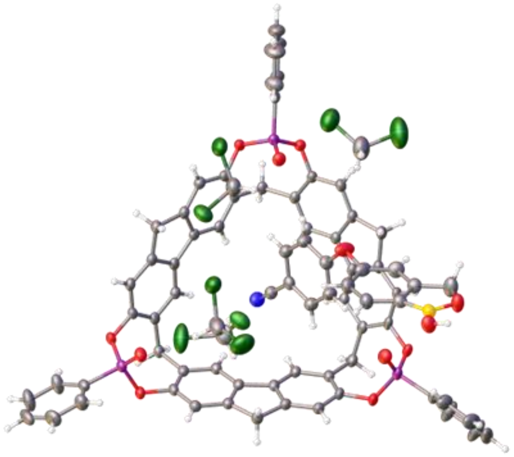

Supplementary Figure 36. X-ray structure of crisaborole (**G14**) @ **F[3]A1-[P(O)Ph]$_3$**. Thermal ellipsoids were shown at 50% probability level.

CCDC number	2313217
Empirical formula	$C_{78}H_{56}BCl_8NO_{12}P_3$
Formula weight	1586.55
Temperature [K]	193.00
Crystal system	triclinic
Space group	$P\overline{1}$
a [Å]	10.7435(6)
b [Å]	18.2383(9)
c [Å]	20.5659(10)
α [°]	66.310(3)
β [°]	87.368(3)
γ [°]	83.009(3)
Volume [Å^3]	3662.7(3)
Z	2
ρ_{calc} [gcm^{-3}]	1.439
μ [mm^{-1}]	3.957
F(000)	1626
Radiation	CuK_α (λ=1.54178 Å)
2θ range [°]	4.69 to 136.87 (0.83 Å)
Index ranges	−12 ≤ h ≤ 12, −21 ≤ k ≤ 17, −24 ≤ l ≤ 21
Reflections collected	59458
Independent reflections	13393, R_{int} = 0.0868, R_{sigma} = 0.0765
Completeness to,θ = 67.679°	99.8 %
Data / Restraints / Parameters	13393/0/957
Absorption correction	0.6193/0.7531
Goodness-of-fit on F^2	1.065
Final *R* indexes, [I≥2σ(I)]	R_1 = 0.0479, wR_2 = 0.1197
Final *R* indexes, [all data]	R_1 = 0.0843, wR_2 = 0.1316
Largest peak/hole [eÅ^{-3}]	0.92/−0.94

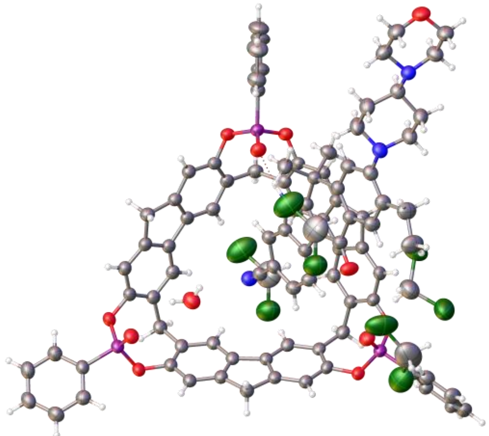

Supplementary Figure 37. X-ray structure of alectinib (**G15**) @ **F[3]A1-[P(O)Ph]$_3$**. Thermal ellipsoids were shown at 50% probability level.

CCDC number	2313233
Empirical formula	$C_{93.25}H_{81.50}Cl_{6.50}N_4O_{11.50}P_3$
Formula weight	1765.45
Temperature [K]	193.00
Crystal system	triclinic
Space group	$P\overline{1}$
a [Å]	14.8265(16)
b [Å]	18.5465(16)
c [Å]	19.8129(16)
α [°]	103.172(5)
β [°]	110.112(4)
γ [°]	90.559(5)
Volume [Å^3]	4958.1(8)
Z	2
ρ_{calc} [gcm^{-3}]	1.183
μ [mm^{-1}]	2.615
F(000)	1833
Radiation	CuK_α (λ=1.54178 Å)
2θ range [°]	4.90 to 137.41 (0.83 Å)
Index ranges	−17 ≤ h ≤ 16, −18 ≤ k ≤ 22, −23 ≤ l ≤ 23
Reflections collected	61543
Independent reflections	18005, R_{int} = 0.0687, R_{sigma} = 0.0697
Completeness to,θ = 67.679°	99.0 %
Data / Restraints / Parameters	18005/81/1129
Absorption correction	0.5896/0.7531
Goodness-of-fit on F^2	1.048
Final *R* indexes, [I≥2σ(I)]	R_1 = 0.0832, wR_2 = 0.2579
Final *R* indexes, [all data]	R_1 = 0.0994, wR_2 = 0.2745
Largest peak/hole [eÅ^{-3}]	1.60/−0.77

2.6. IGMH analysis

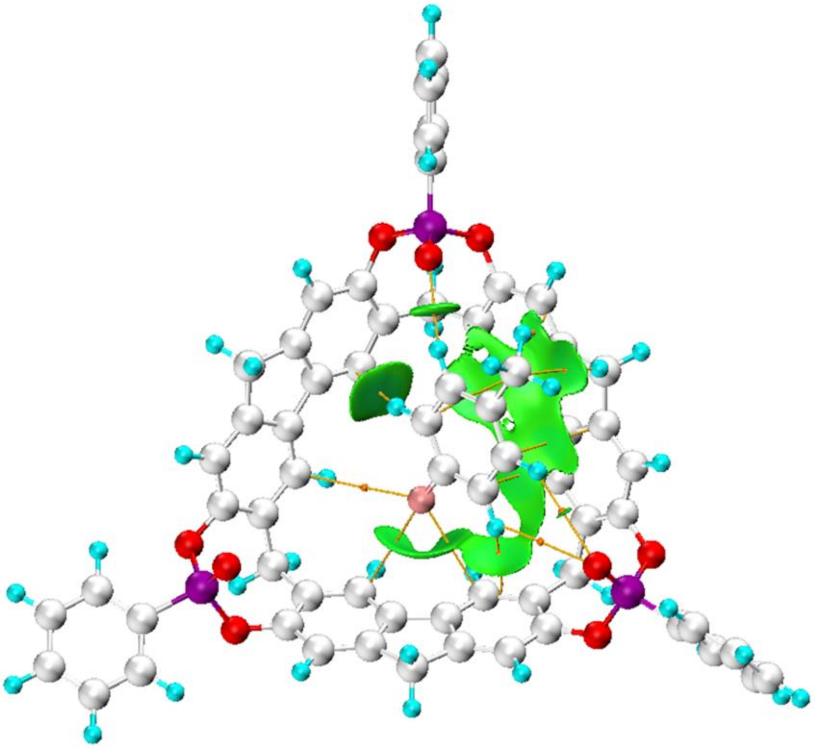

Supplementary Figure 38. The **IGMH** analysis for the structure of *p*-fluorotoluene @ **F[3]A1-[P(O)Ph]$_3$**

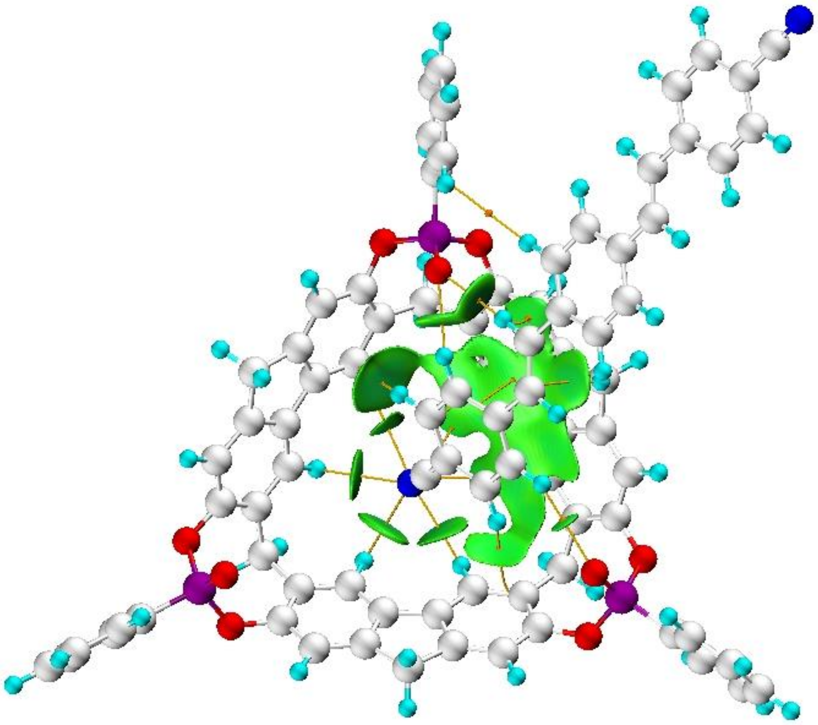

Supplementary Figure 39. The **IGMH** analysis for the structure of 1,4-bis(4-cyanostyryl)-benzene (**G13**) @ **F[3]A1-[P(O)Ph]$_3$**

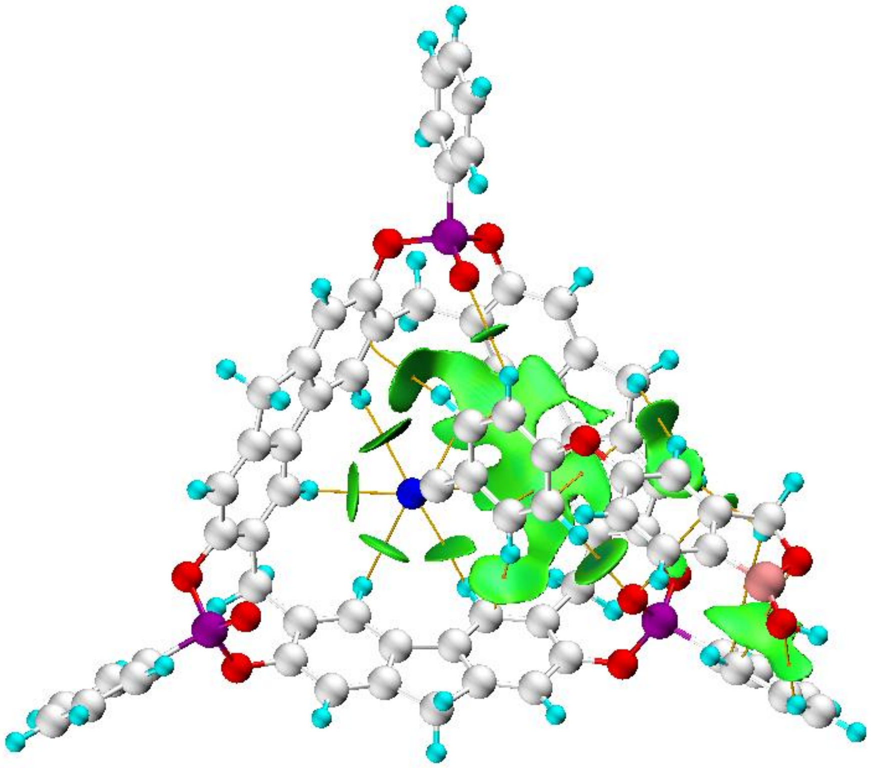

Supplementary Figure 40. The **IGMH** analysis for the structure of crisaborole (**G14**) @ **F[3]A1-[P(O)Ph]$_3$**

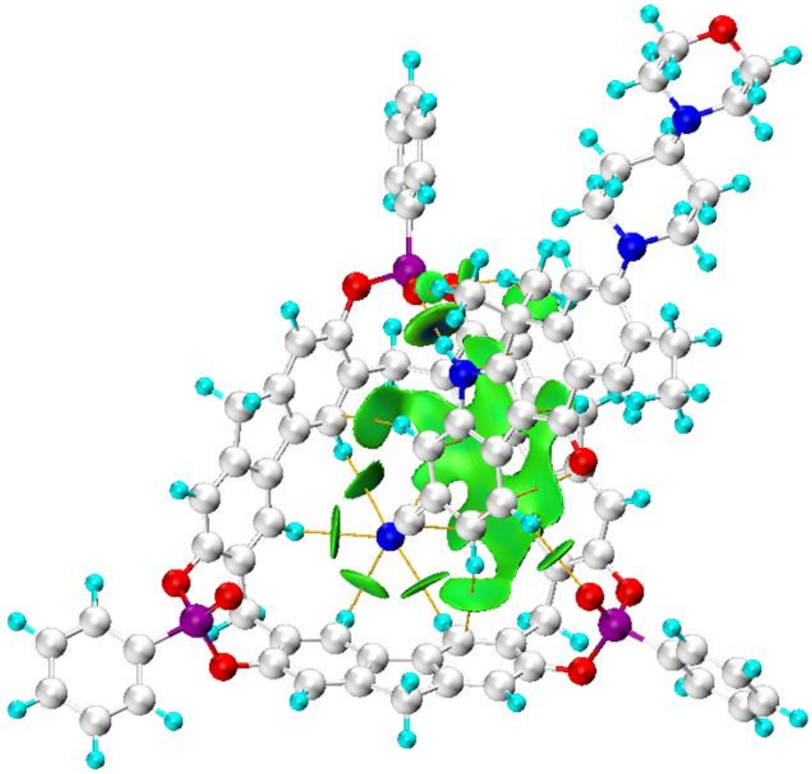

Supplementary Figure 41. The **IGMH** analysis for the structure of alectinib (**G15**) @ **F[3]A1-[P(O)Ph]$_3$**

2.7. ^{1}H NMR spectra of titration experiment details between benzonitrile and F[3]A1-[P(O)Ph]$_3$.

To determine the association constant between **F[3]A1-[P(O)Ph]$_3$** and **benzonitrile**, **^{1}H NMR** titrations were carried out in aqueous solution, which had a constant concentration of **F[3]A1-[P(O)Ph]$_3$** (3.0 mM) and varying concentrations of **benzonitrile**. By a non-linear curve-fitting method, the association constant between **benzonitrile** and **F[3]A1-[P(O)Ph]$_3$** was calculated. The non-linear curve-fitting was based on the following equation: $\mathbf{\Delta\delta = (\Delta\delta_{\infty}/[G]_0)(0.5[H]_0+0.5([G]_0+1/K_a)-(0.5([H]_0^2+(2[H]_0(1/K_a-[G]_0))+(1/K_a+[G]_0)^2)^{0.5}))}$. Where $\Delta\delta$ is the chemical shift change of H_a on **G** at $[H]_0$, $\Delta\delta_{\infty}$ is the chemical shift change of H_a when the guest is completely complexed, $[G]_0$ is the fixed initial concentration of the guest (**G**), and $[H]_0$ is the varying concentrations of **F[3]A1-[P(O)Ph]$_3$**.

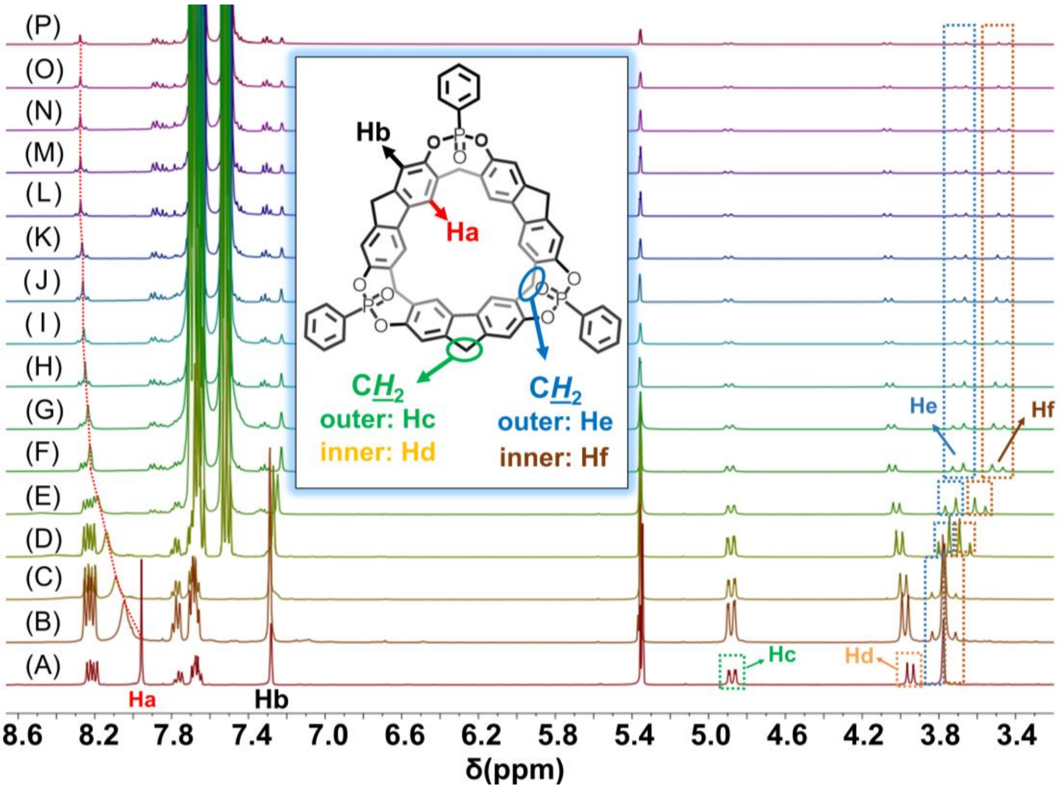

Supplementary Figure 42. ^{1}H NMR spectra of the titration experiment of F[3]A1-[P(O)Ph]$_3$ with benzonitrile carried out in dichloromethane-*d*. ^{1}H **NMR** spectra (400 **MHz**, CCl_2D_2, 298 K) of **F[3]A1-[P(O)Ph]$_3$** at a constant concentration of 3.0 **mM** with different concentrations of benzonitrile (**mM**): (A) 0.0, (B) 0.4, (C) 0.8, (D) 1.6, (E) 2.4, (F) 3.2, (G) 4.0, (H) 4.8, (I) 5.6, (J) 6.4, (K) 7.2, (L) 8.0, (M) 8.8, (N) 9.6, (O) 10.4, and (P) 11.2.

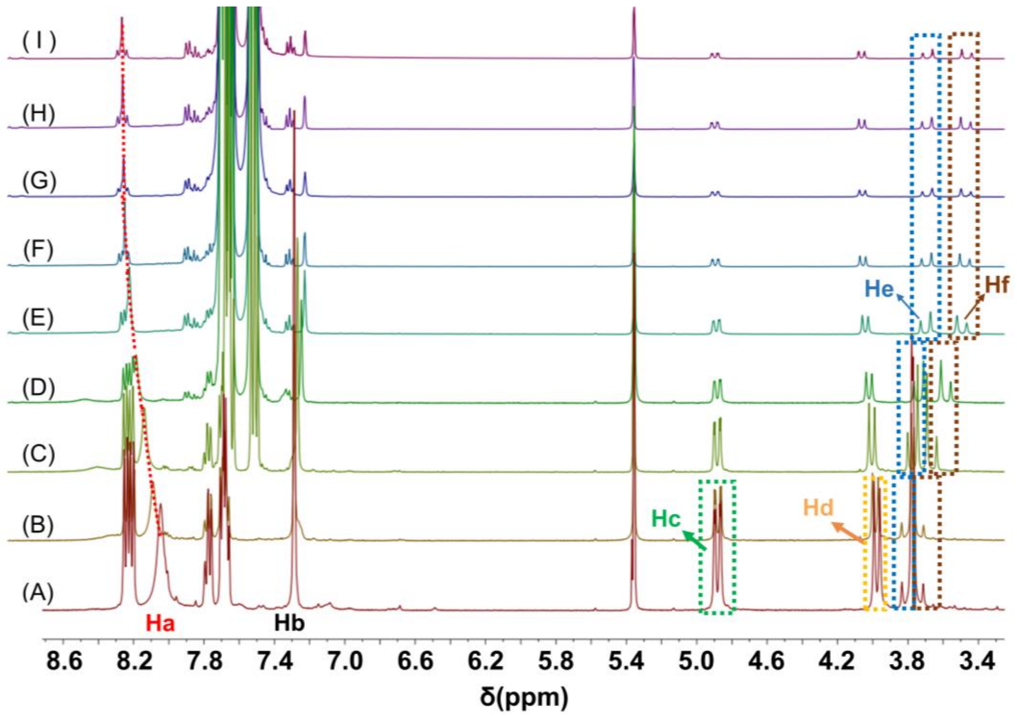

Supplementary Figure 43. Job plot of complex F[3]A1-[P(O)Ph]$_3$ with benzonitrile carried out in dichloromethane-*d.* ^{1}H **NMR** spectra (400 **MHz**, CCl_2D_2, 298 K) of the concentration ratios of **F[3]A1-[P(O)Ph]$_3$** to benzonitrile were as follows: (A) 9:1, (B) 8:2, (C)7:3, (D)6:4, (E) 5:5, (F) 4:6, (G)3:7, (H)2:8, and (I)1:9.

2.8. UV-vis titration experiments details between guest (G1-G15) and F[3]A1-[P(O)Ph]$_3$.

To determine the association constant for the complexation between **F[3]A1-[P(O)Ph]$_3$** and the guests (**G1**-**G15**), UV-vis titration experiments were carried out in **$CHCl_3$**, which had a constant concentration of **F[3]A1-[P(O)Ph]$_3$** (0.01 mM) and varying concentrations of the guests (**G1**-**G15**). By a non-linear curve-fitting method, the association constant (***K_a***) of **F[3]A1-[P(O)Ph]$_3$** @ the guests (**G1**-**G15**) were estimated. The non-linear curve-fittings were based on the following equation:

$$\Delta A = (\Delta A_\infty/[H]_0)*(0.5*[G]_0+0.5*([H]_0+1/K_a)-(0.5*([G]_0\text{^}2+(2*[G]_0*(1/K_a-[H]_0))+(1/K_a+[H]_0)\text{^}2)\text{^}0.5))$$

Where the ΔA is the UV-vis absorption change of **F[3]A1-[P(O)Ph]$_3$** upon addition of the guests (**G1**-**G15**), ΔA_∞ is the UV-vis absorption changes at 265 nm when the **F[3]A1-[P(O)Ph]$_3$** is completely complexed, $[H]_0$ is the initial concentration of **F[3]A1-[P(O)Ph]$_3$**, and $[G]_0$ is the varying concentration of the guests (**G1**-**G15**).

Supplementary Table 1. Details of binding constants for all host-guest complexes formed

Host-guest complexes	K_a	R^2
**F[3]A1-[P(O)Ph]3 @ G1** (benzonitrile)	$(4.648 \pm 0.372) \times 10^3\ M^{-1}$	> 0.998
**F[3]A1-[P(O)Ph]$_3$** @ **G2** (3,4-difluorobenzonitrile)	$(1.087 \pm 0.068) \times 10^4\ M^{-1}$	> 0.991
**F[3]A1-[P(O)Ph]$_3$** @ **G3** (4-chlorobenzonitrile)	$(1.865 \pm 0.087) \times 10^4\ M^{-1}$	> 0.993
**F[3]A1-[P(O)Ph]$_3$** @ **G4** (4-bromobenzonitrile)	$(1.972 \pm 0.046) \times 10^4\ M^{-1}$	> 0.998
**F[3]A1-[P(O)Ph]$_3$** @ **G5** (4-fluoro-3-hydroxybenzonitrile)	$(6.754 \pm 0.356) \times 10^3\ M^{-1}$	> 0.993
**F[3]A1-[P(O)Ph]$_3$** @ **G6** (4-trifluoromethylbenzonitrile)	$(6.253 \pm 0.377) \times 10^3\ M^{-1}$	> 0.994
**F[3]A1-[P(O)Ph]$_3$** @ **G7** (4-ethylbenzonitrile)	$(6.446 \pm 0.339) \times 10^3\ M^{-1}$	> 0.993
**F[3]A1-[P(O)Ph]$_3$** @ **G8** (4-butylbenzonitrile)	$(8.065 \pm 0.491) \times 10^3\ M^{-1}$	> 0.991
**F[3]A1-[P(O)Ph]$_3$** @ **G9** (3,4-dihydroxybenzonitrile)	$(1.808 \pm 0.041) \times 10^4\ M^{-1}$	> 0.998
**F[3]A1-[P(O)Ph]$_3$** @ **G10** (3-fluoro-4-hydroxybenzonitrile)	$(1.455 \pm 0.051) \times 10^4\ M^{-1}$	> 0.996
**F[3]A1-[P(O)Ph]$_3$** @ **G11** (4-cyanobenzenesulfonamide)	$(1.344 \pm 0.037) \times 10^4\ M^{-1}$	> 0.998
**F[3]A1-[P(O)Ph]$_3$** @ **G12** (4'-Hydroxy-4-biphenylcarbonitrile)	$(1.932 \pm 0.055) \times 10^4\ M^{-1}$	> 0.997
**F[3]A1-[P(O)Ph]$_3$** @ **G13** (1,4-bis(4-cyanostyryl)benzene)	$(2.158 \pm 0.076) \times 10^4\ M^{-1}$	> 0.996
**F[3]A1-[P(O)Ph]$_3$** @ **G14** (crisaborole)	$(1.150 \pm 0.063) \times 10^4\ M^{-1}$	> 0.991
**F[3]A1-[P(O)Ph]$_3$** @ **G15** (alectinib)	$(1.669 \pm 0.046) \times 10^4\ M^{-1}$	> 0.997

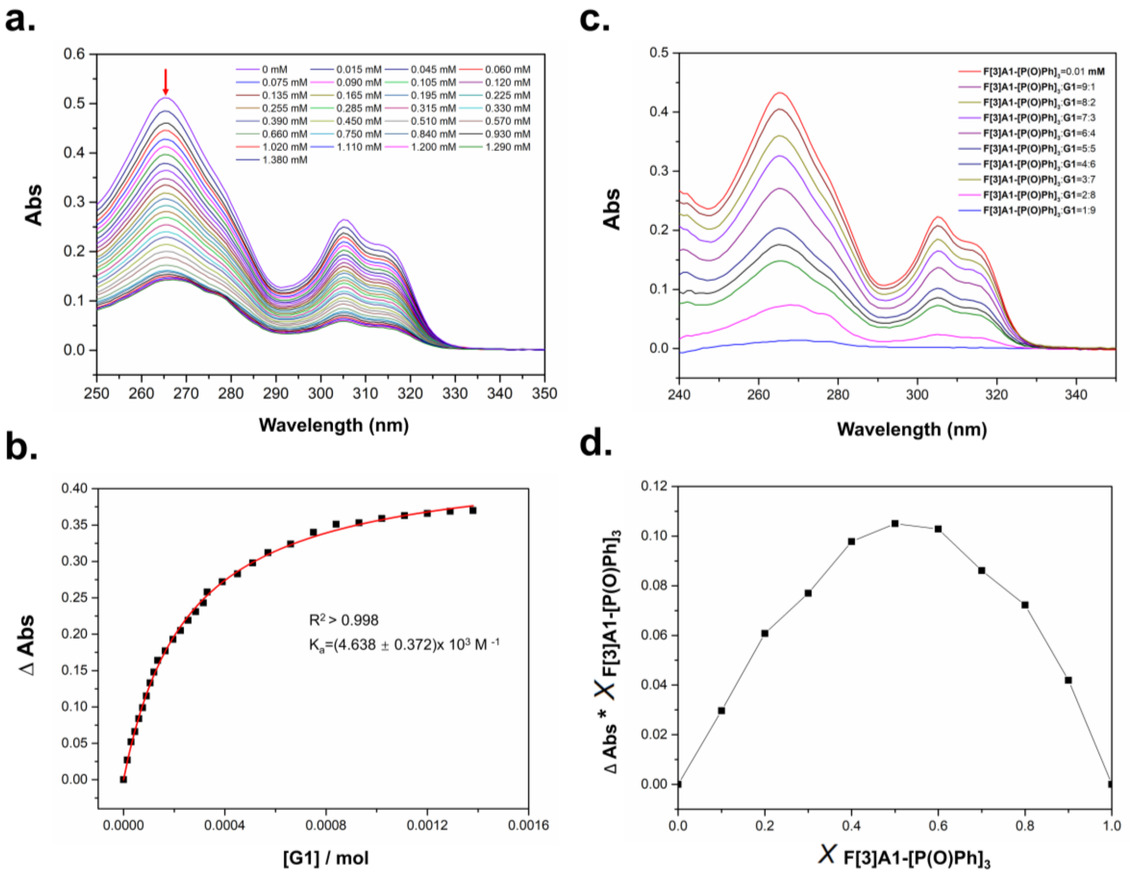

Supplementary Figure 44. UV–Vis titration experiments of F[3]A1-[P(O)Ph]$_3$ with G1 (benzonitrile) (a). **UV–Vis** absorption spectra of **F[3]A1-[P(O)Ph]$_3$** at a constant concentration of 0.01 mM with different concentrations of **G1** (benzonitrile), ranging from 0 mM to 1.38 mM. (b). The absorbance changes of **F[3]A1-[P(O)Ph]$_3$** upon the addition of **G1**. The red solid line was obtained from the non-linear curve fitting. The association constant (***K$_a$***) between **F[3]A1-[P(O)Ph]$_3$** and **G1** was estimated to be (4.648 ± 0.372) × 10^3 M^{-1}. (c). **UV-Vis** absorption spectra of complex **F[3]A1-[P(O)Ph]$_3$ @ G1** with different molar ratios in water while [**F[3]A1-[P(O)Ph]$_3$**] + [**G1**] = 0.10 mM. (d). Job plots of the complex **F[3]A1-[P(O)Ph]$_3$ @ G1** showing a 1:1 stoichiometry between **F[3]A1-[P(O)Ph]$_3$** and **G1** by plotting the absorbance differences at 265 nm (a characteristic absorption peak of **F[3]A1-[P(O)Ph]$_3$**) against the mole fraction of **G1**.

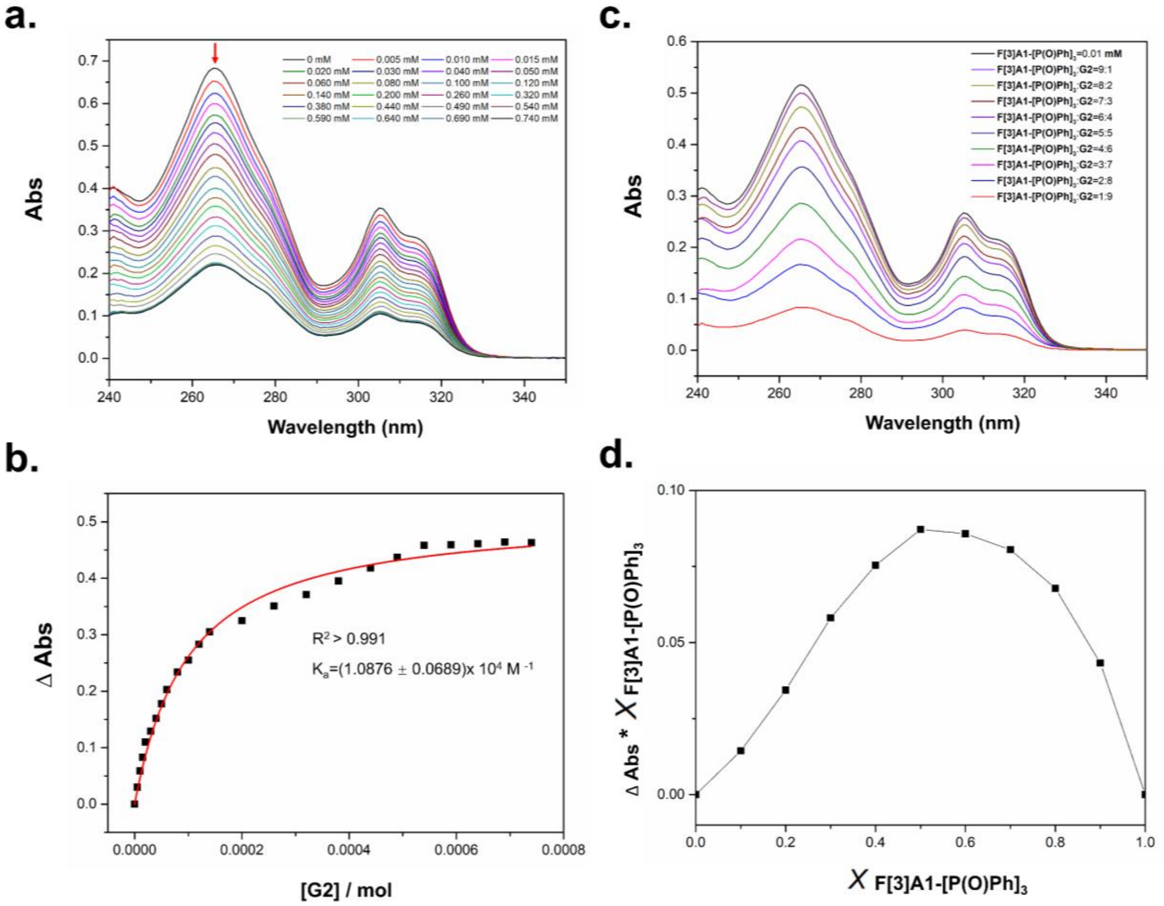

Supplementary Figure 45. UV–Vis titration experiments of F[3]A1-[P(O)Ph]$_3$ with G2 (3,4-difluorobenzonitrile). (a). **UV–Vis** absorption spectra of **F[3]A1-[P(O)Ph]$_3$** at a constant concentration of 0.01 mM with different concentrations of **G2** (3,4-difluorobenzonitrile), ranging from 0 mM to 0.74 mM. (b). The absorbance changes of **F[3]A1-[P(O)Ph]$_3$** upon the addition of **G2**. The red solid line was obtained from the non-linear curve fitting. The association constant (K_a) between **F[3]A1-[P(O)Ph]$_3$** and **G2** was estimated to be (1.0876 ± 0.0689) × 10^4 M^{-1}. (c). **UV-Vis** absorption spectra of complex **F[3]A1-[P(O)Ph]$_3$ @ G2** with different molar ratios in water while [**F[3]A1-[P(O)Ph]$_3$**] + [**G2**] = 0.10 mM. (d). Job plots of the complex **F[3]A1-[P(O)Ph]$_3$ @ G2** showing a 1:1 stoichiometry between **F[3]A1-[P(O)Ph]$_3$** and **G2** by plotting the absorbance differences at 265 nm (a characteristic absorption peak of **F[3]A1-[P(O)Ph]$_3$**) against the mole fraction of **G2**.

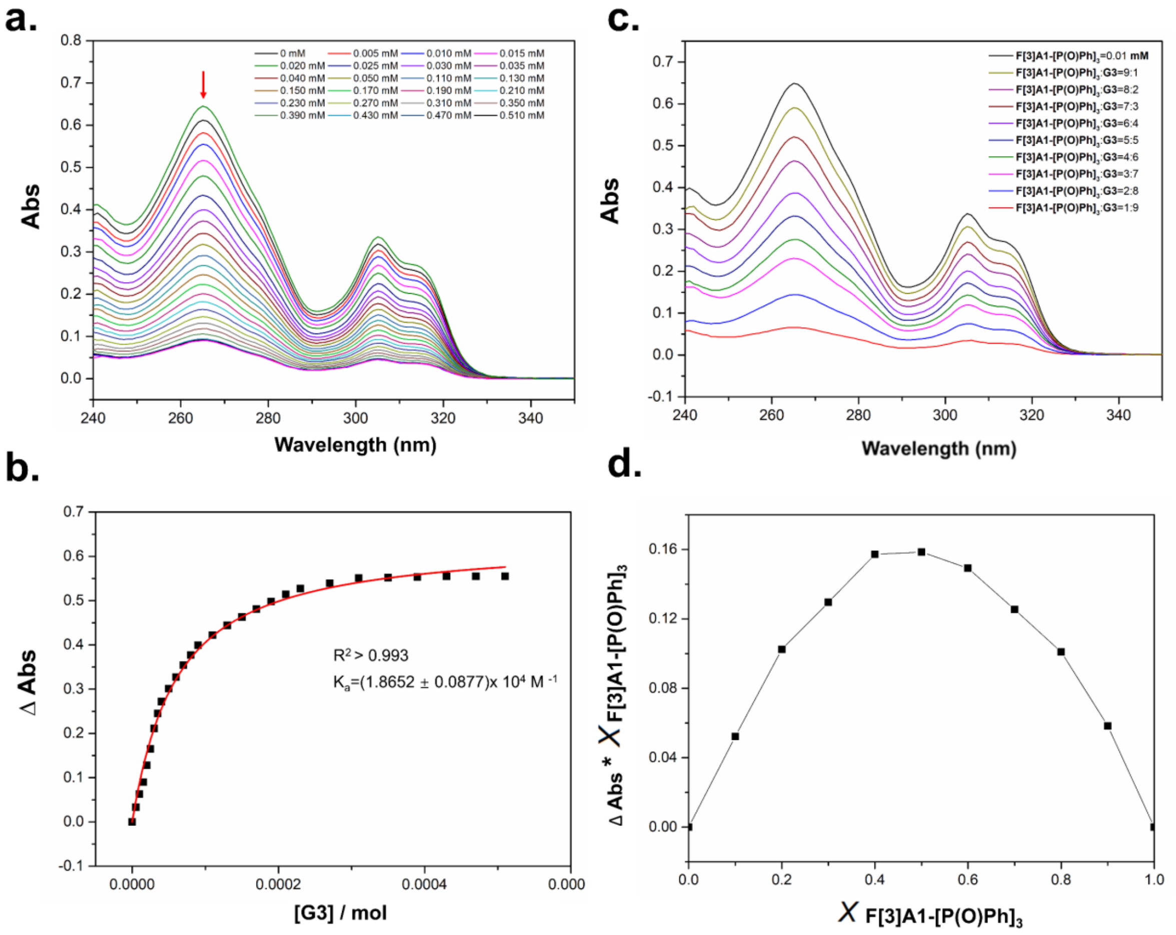

Supplementary Figure 46. UV–Vis titration experiments of F[3]A1-[P(O)Ph]$_3$ with G3 (4-chlorobenzonitrile). (a). **UV–Vis** absorption spectra of **F[3]A1-[P(O)Ph]$_3$** at a constant concentration of 0.01 mM with different concentrations of **G3** (4-chlorobenzonitrile), ranging from 0 mM to 0.51 mM. (b). The absorbance changes of **F[3]A1-[P(O)Ph]$_3$** upon the addition of **G3**. The red solid line was obtained from the non-linear curve fitting. The association constant (***K$_a$***) between **F[3]A1-[P(O)Ph]$_3$** and **G3** was estimated to be (1.8652 ± 0.0877) × 10^4 M^{-1}. (c). **UV-Vis** absorption spectra of complex **F[3]A1-[P(O)Ph]$_3$ @ G3** with different molar ratios in water while [**F[3]A1-[P(O)Ph]$_3$**] + [**G3**] = 0.10 mM. (d). Job plots of the complex **F[3]A1-[P(O)Ph]$_3$ @ G3** showing a 1:1 stoichiometry between **F[3]A1-[P(O)Ph]$_3$** and **G3** by plotting the absorbance differences at 265 nm (a characteristic absorption peak of **F[3]A1-[P(O)Ph]$_3$**) against the mole fraction of **G3**.

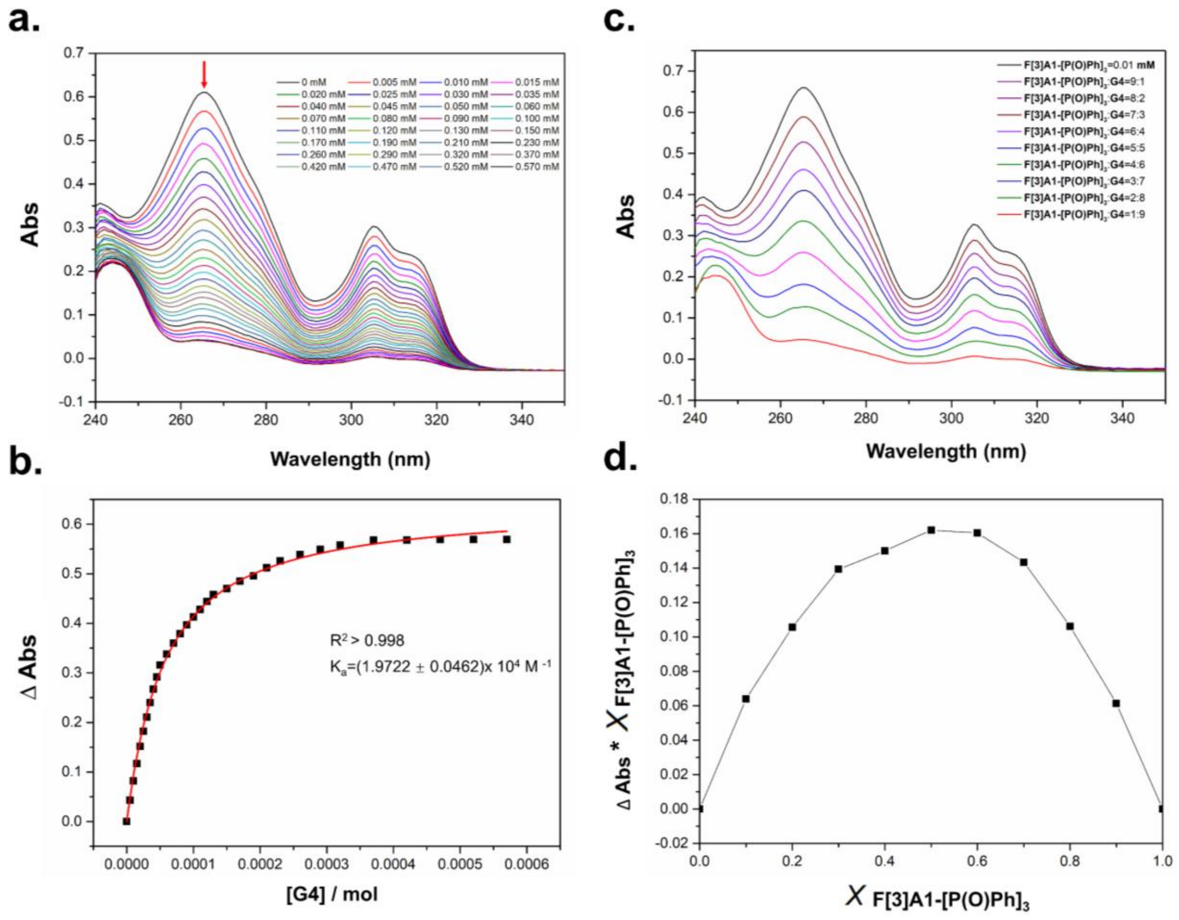

Supplementary Figure 47. UV–Vis titration experiments of F[3]A1-[P(O)Ph]$_3$ with G4 (4-bromobenzonitrile) . (a). **UV–Vis** absorption spectra of **F[3]A1-[P(O)Ph]$_3$** at a constant concentration of 0.01 mM with different concentrations of **G4** (4-bromobenzonitrile), ranging from 0 mM to 0.57 mM. (b). The absorbance changes of **F[3]A1-[P(O)Ph]$_3$** upon the addition of **G4**. The red solid line was obtained from the non-linear curve fitting. The association constant (K_a) between **F[3]A1-[P(O)Ph]$_3$** and **G4** was estimated to be (1.9722 ± 0.0462) × 10^4 M^{-1}. (c). **UV-Vis** absorption spectra of complex **F[3]A1-[P(O)Ph]$_3$ @ G4** with different molar ratios in water while [**F[3]A1-[P(O)Ph]$_3$**] + [**G4**] = 0.10 mM. (d). Job plots of the complex **F[3]A1-[P(O)Ph]$_3$ @ G4** showing a 1:1 stoichiometry between **F[3]A1-[P(O)Ph]$_3$** and **G4** by plotting the absorbance differences at 265 nm (a characteristic absorption peak of **F[3]A1-[P(O)Ph]$_3$**) against the mole fraction of **G4**.

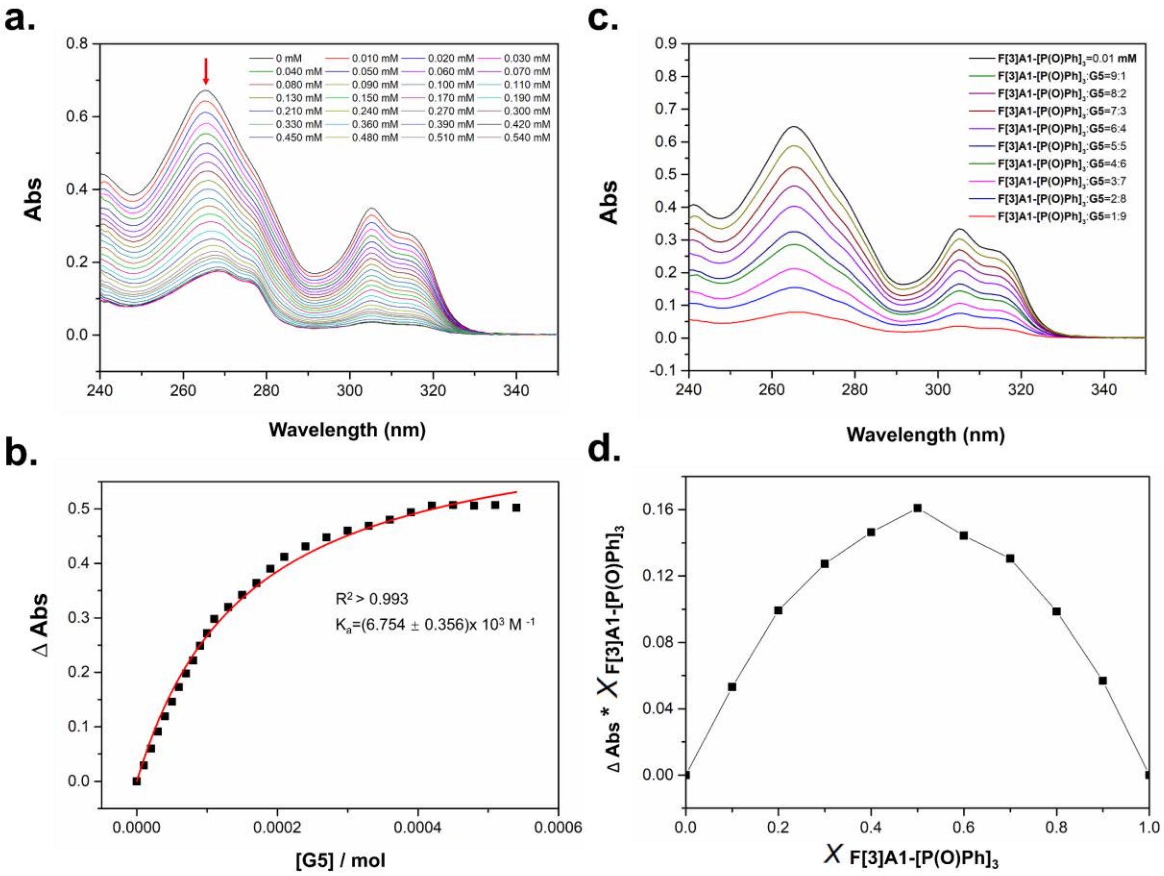

Supplementary Figure 48. UV–Vis titration experiments of F[3]A1-[P(O)Ph]$_3$ with G5 (4-fluoro-3-hydroxybenzonitrile). (a). **UV–Vis** absorption spectra of **F[3]A1-[P(O)Ph]$_3$** at a constant concentration of 0.01 mM with different concentrations of **G5** (4-fluoro-3-hydroxybenzonitrile), ranging from 0 mM to 0.54 mM. (b). The absorbance changes of **F[3]A1-[P(O)Ph]$_3$** upon the addition of **G5**. The red solid line was obtained from the non-linear curve fitting. The association constant (K_a) between **F[3]A1-[P(O)Ph]$_3$** and **G5** was estimated to be (6.754 ± 0.356) × 10^3 M^{-1}. (c). **UV-Vis** absorption spectra of complex **F[3]A1-[P(O)Ph]$_3$ @ G5** with different molar ratios in water while [**F[3]A1-[P(O)Ph]$_3$**] + [**G5**] = 0.10 mM. (d). Job plots of the complex **F[3]A1-[P(O)Ph]$_3$ @ G5** showing a 1:1 stoichiometry between **F[3]A1-[P(O)Ph]$_3$** and **G5** by plotting the absorbance differences at 265 nm (a characteristic absorption peak of **F[3]A1-[P(O)Ph]$_3$**) against the mole fraction of **G5**.

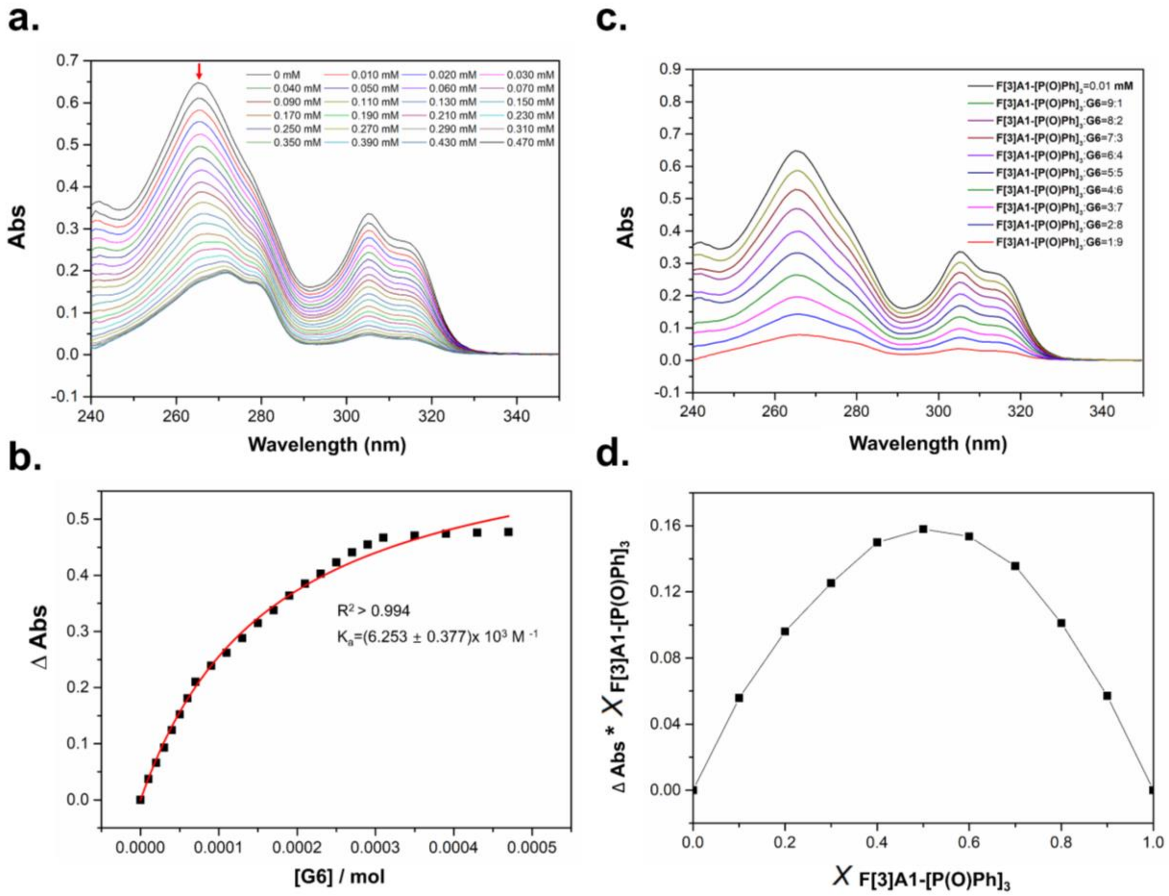

Supplementary Figure 49. UV–Vis titration experiments of F[3]A1-[P(O)Ph]$_3$ with G6 (4-trifluoromethylbenzonitrile). (a). **UV–Vis** absorption spectra of **F[3]A1-[P(O)Ph]$_3$** at a constant concentration of 0.01 mM with different concentrations of **G6** (4-trifluoromethylbenzonitrile), ranging from 0 mM to 0.47 mM. (b). The absorbance changes of **F[3]A1-[P(O)Ph]$_3$** upon the addition of **G6**. The red solid line was obtained from the non-linear curve fitting. The association constant (K_a) between **F[3]A1-[P(O)Ph]$_3$** and **G6** was estimated to be (6.253 ± 0.377) × 10^3 M^{-1}. (c). **UV-Vis** absorption spectra of complex **F[3]A1-[P(O)Ph]$_3$ @ G6** with different molar ratios in water while [**F[3]A1-[P(O)Ph]$_3$**] + [**G6**] = 0.10 mM. (d). Job plots of the complex **F[3]A1-[P(O)Ph]$_3$ @ G6** showing a 1:1 stoichiometry between **F[3]A1-[P(O)Ph]$_3$** and **G6** by plotting the absorbance differences at 265 nm (a characteristic absorption peak of **F[3]A1-[P(O)Ph]$_3$**) against the mole fraction of **G6**.

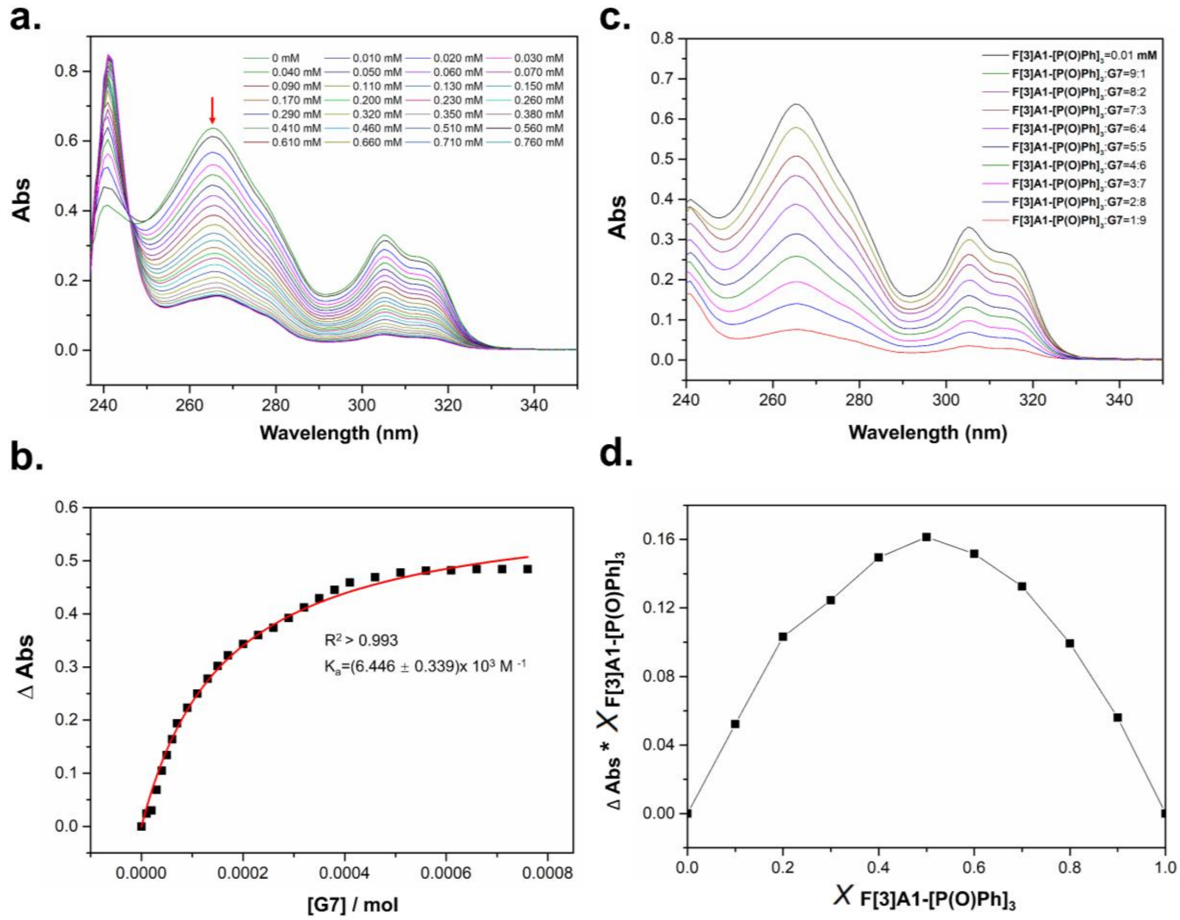

Supplementary Figure 50. UV–Vis titration experiments of F[3]A1-[P(O)Ph]$_3$ with G7 (4-ethylbenzonitrile). (a). **UV–Vis** absorption spectra of **F[3]A1-[P(O)Ph]$_3$** at a constant concentration of 0.01 mM with different concentrations of **G7** (4-ethylbenzonitrile), ranging from 0 mM to 0.76 mM. (b). The absorbance changes of **F[3]A1-[P(O)Ph]$_3$** upon the addition of **G7**. The red solid line was obtained from the non-linear curve fitting. The association constant ($\boldsymbol{K_a}$) between **F[3]A1-[P(O)Ph]$_3$** and **G7** was estimated to be (6.446 ± 0.339) × 10^3 M^{-1}. (c). **UV-Vis** absorption spectra of complex **F[3]A1-[P(O)Ph]$_3$ @ G7** with different molar ratios in water while [**F[3]A1-[P(O)Ph]$_3$**] + [**G7**] = 0.10 mM. (d). Job plots of the complex **F[3]A1-[P(O)Ph]$_3$ @ G7** showing a 1:1 stoichiometry between **F[3]A1-[P(O)Ph]$_3$** and **G7** by plotting the absorbance differences at 265 nm (a characteristic absorption peak of **F[3]A1-[P(O)Ph]$_3$**) against the mole fraction of **G7**.

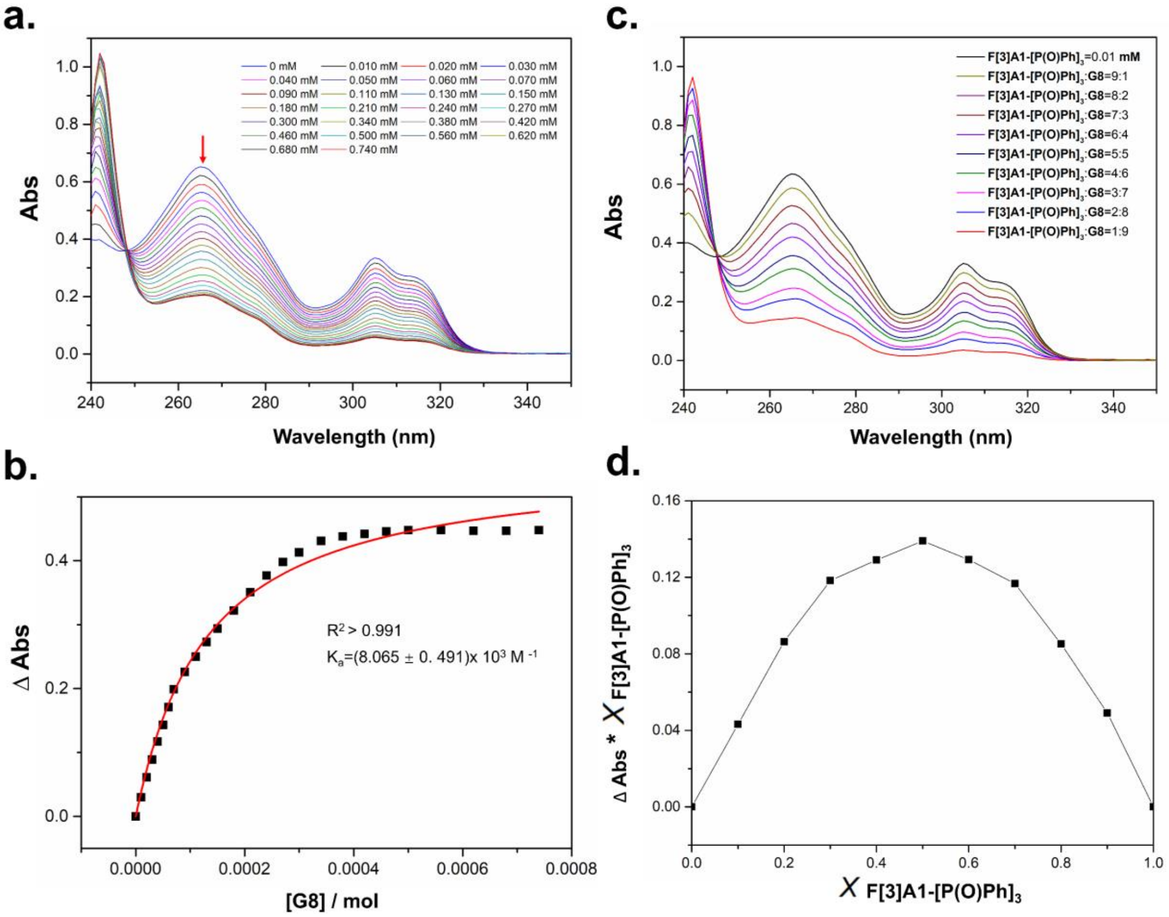

Supplementary Figure 51. UV–Vis titration experiments of F[3]A1-[P(O)Ph]$_3$ with G8 (4-butylbenzonitrile). (a). **UV–Vis** absorption spectra of **F[3]A1-[P(O)Ph]$_3$** at a constant concentration of 0.01 mM with different concentrations of **G8** (4-butylbenzonitrile), ranging from 0 mM to 0.74 mM. (b). The absorbance changes of **F[3]A1-[P(O)Ph]$_3$** upon the addition of **G8**. The red solid line was obtained from the non-linear curve fitting. The association constant (***K_a***) between **F[3]A1-[P(O)Ph]$_3$** and **G8** was estimated to be (8.065 ± 0.491) × 10^3 M^{-1}. (c). **UV-Vis** absorption spectra of complex **F[3]A1-[P(O)Ph]$_3$ @ G8** with different molar ratios in water while [**F[3]A1-[P(O)Ph]$_3$**] + [**G8**] = 0.10 mM. (d). Job plots of the complex **F[3]A1-[P(O)Ph]$_3$ @ G8** showing a 1:1 stoichiometry between **F[3]A1-[P(O)Ph]$_3$** and **G8** by plotting the absorbance differences at 265 nm (a characteristic absorption peak of **F[3]A1-[P(O)Ph]$_3$**) against the mole fraction of **G8**.

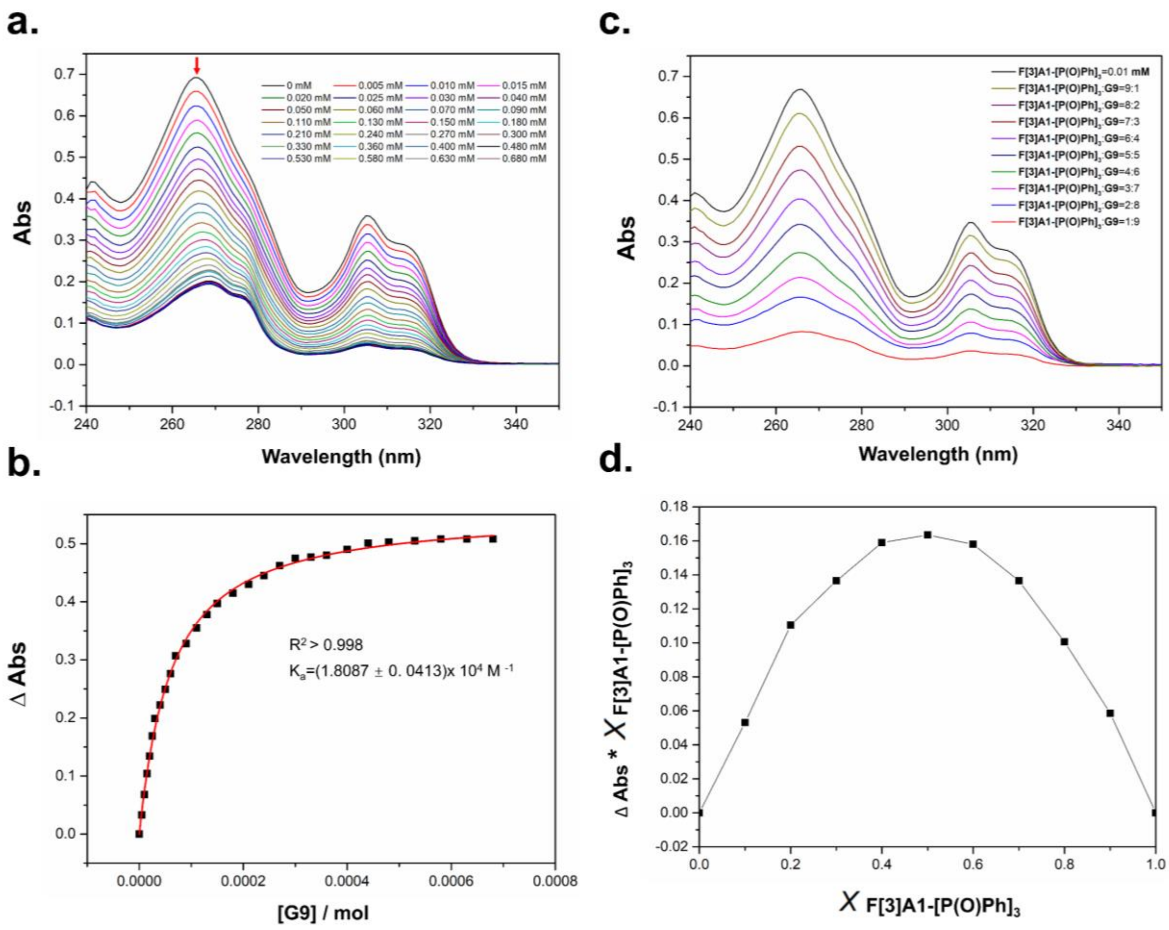

Supplementary Figure 52. UV–Vis titration experiments of F[3]A1-[P(O)Ph]3 with G9 (3,4-dihydroxybenzonitrile). **(**a). **UV–Vis** absorption spectra of **F[3]A1-[P(O)Ph]3** at a constant concentration of 0.01 mM with different concentrations of **G9** (3,4-dihydroxybenzonitrile), ranging from 0 mM to 0.68 mM. (b). The absorbance changes of **F[3]A1-[P(O)Ph]3** upon the addition of **G9**. The red solid line was obtained from the non-linear curve fitting. The association constant ($\boldsymbol{K_a}$) between **F[3]A1-[P(O)Ph]3** and **G9** was estimated to be (1.8087 ± 0.0413) × 10^4 M^{-1}. (c). **UV-Vis** absorption spectra of complex **F[3]A1-[P(O)Ph]3 @ G9** with different molar ratios in water while [**F[3]A1-[P(O)Ph]3**] + [**G9**] = 0.10 mM. (d). Job plots of the complex **F[3]A1-[P(O)Ph]3 @ G9** showing a 1:1 stoichiometry between **F[3]A1-[P(O)Ph]3** and **G9** by plotting the absorbance differences at 265 nm (a characteristic absorption peak of **F[3]A1-[P(O)Ph]3**) against the mole fraction of **G9**.

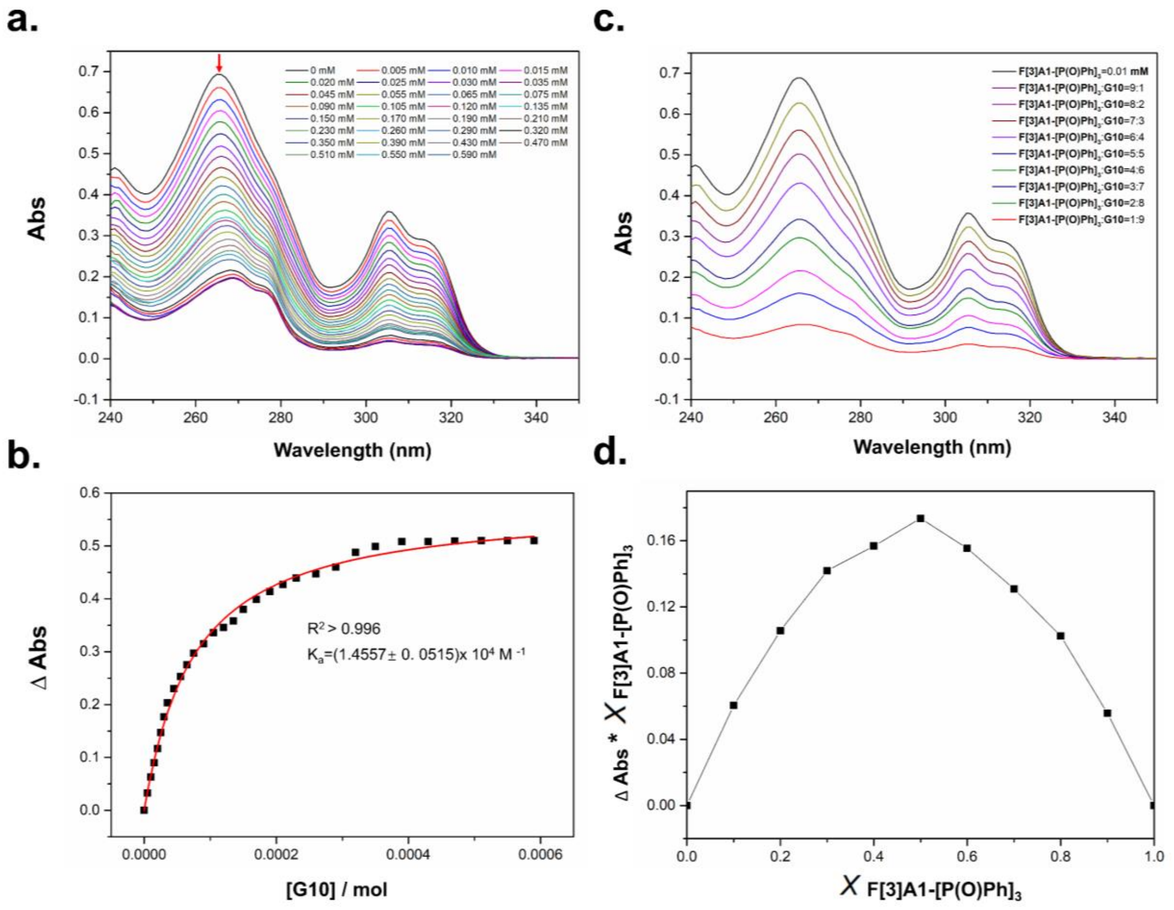

Supplementary Figure 53. UV–Vis titration experiments of F[3]A1-[P(O)Ph]$_3$ with G10 (3-fluoro-4-hydroxybenzonitrile). (a). **UV–Vis** absorption spectra of **F[3]A1-[P(O)Ph]$_3$** at a constant concentration of 0.01 mM with different concentrations of **G10** (3-fluoro-4-hydroxybenzonitrile), ranging from 0 mM to 0.59 mM. (b). The absorbance changes of **F[3]A1-[P(O)Ph]$_3$** upon the addition of **G10**. The red solid line was obtained from the non-linear curve fitting. The association constant (***K_a***) between **F[3]A1-[P(O)Ph]$_3$** and **G10** was estimated to be (1.4557 ± 0.0515) × 10^4 M^{-1}. (c). **UV-Vis** absorption spectra of complex **F[3]A1-[P(O)Ph]$_3$ @ G10** with different molar ratios in water while [**F[3]A1-[P(O)Ph]$_3$**] + [**G10**] = 0.10 mM. (d). Job plots of the complex **F[3]A1-[P(O)Ph]$_3$ @ G10** showing a 1:1 stoichiometry between **F[3]A1-[P(O)Ph]$_3$** and **G10** by plotting the absorbance differences at 265 nm (a characteristic absorption peak of **F[3]A1-[P(O)Ph]$_3$**) against the mole fraction of **G10**.

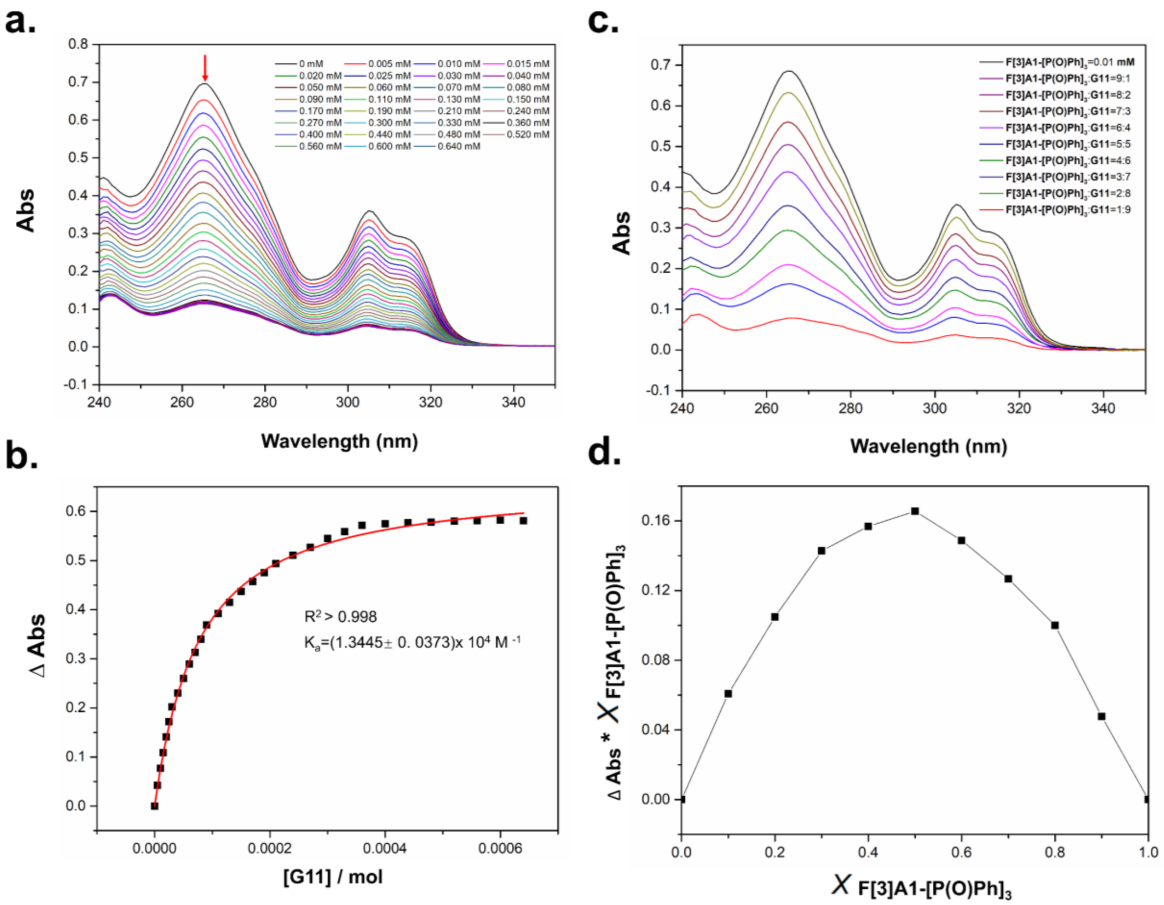

Supplementary Figure 54. UV–Vis titration experiments of F[3]A1-[P(O)Ph]$_3$ with G11 (4-cyanobenzenesulfonamide). (a). **UV–Vis** absorption spectra of **F[3]A1-[P(O)Ph]$_3$** at a constant concentration of 0.01 mM with different concentrations of **G11** (4-cyanobenzenesulfonamide), ranging from 0 mM to 0.64 mM. (b). The absorbance changes of **F[3]A1-[P(O)Ph]$_3$** upon the addition of **G11**. The red solid line was obtained from the non-linear curve fitting. The association constant (K_a) between **F[3]A1-[P(O)Ph]$_3$** and **G11** was estimated to be (1.3445 ± 0.0373) × 10^4 M^{-1}. (c). **UV-Vis** absorption spectra of complex **F[3]A1-[P(O)Ph]$_3$ @ G11** with different molar ratios in water while [**F[3]A1-[P(O)Ph]$_3$**] + [**G11**] = 0.10 mM. (d). Job plots of the complex **F[3]A1-[P(O)Ph]$_3$ @ G11** showing a 1:1 stoichiometry between **F[3]A1-[P(O)Ph]$_3$** and **G11** by plotting the absorbance differences at 265 nm (a characteristic absorption peak of **F[3]A1-[P(O)Ph]$_3$**) against the mole fraction of **G11**.

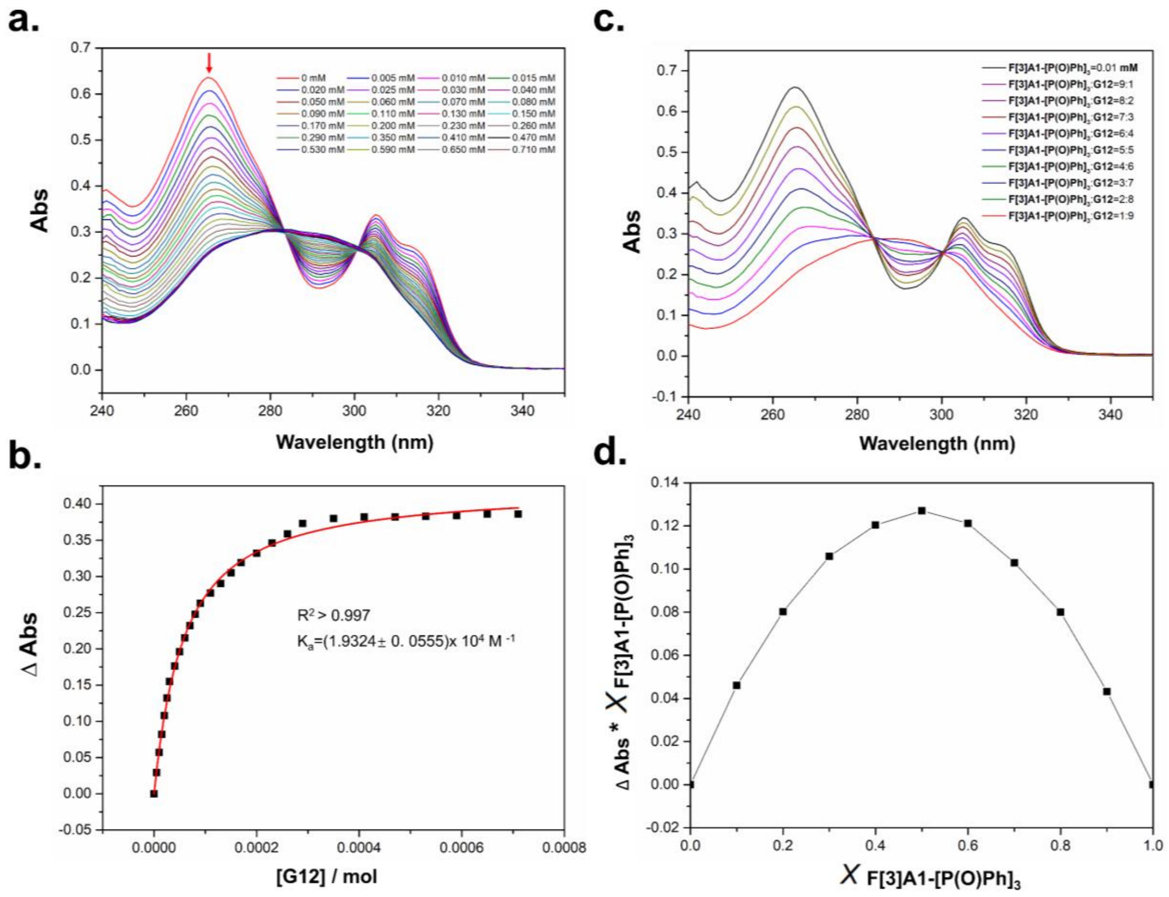

Supplementary Figure 55. UV–Vis titration experiments of F[3]A1-[P(O)Ph]$_3$ with G12 (4'-Hydroxy-4-biphenylcarbonitrile). (a). **UV–Vis** absorption spectra of **F[3]A1-[P(O)Ph]$_3$** at a constant concentration of 0.01 mM with different concentrations of **G12** (4'-Hydroxy-4-biphenylcarbonitrile), ranging from 0 mM to 0.71 mM. (b). The absorbance changes of **F[3]A1-[P(O)Ph]$_3$** upon the addition of **G12**. The red solid line was obtained from the non-linear curve fitting. The association constant (K_a) between **F[3]A1-[P(O)Ph]$_3$** and **G12** was estimated to be (1.9324 ± 0.0555) × 10^4 M^{-1}. (c). **UV-Vis** absorption spectra of complex **F[3]A1-[P(O)Ph]$_3$ @ G12** with different molar ratios in water while [**F[3]A1-[P(O)Ph]$_3$**] + [**G12**] = 0.10 mM. (d). Job plots of the complex **F[3]A1-[P(O)Ph]$_3$ @ G12** showing a 1:1 stoichiometry between **F[3]A1-[P(O)Ph]$_3$** and **G12** by plotting the absorbance differences at 265 nm (a characteristic absorption peak of **F[3]A1-[P(O)Ph]$_3$**) against the mole fraction of **G12**.

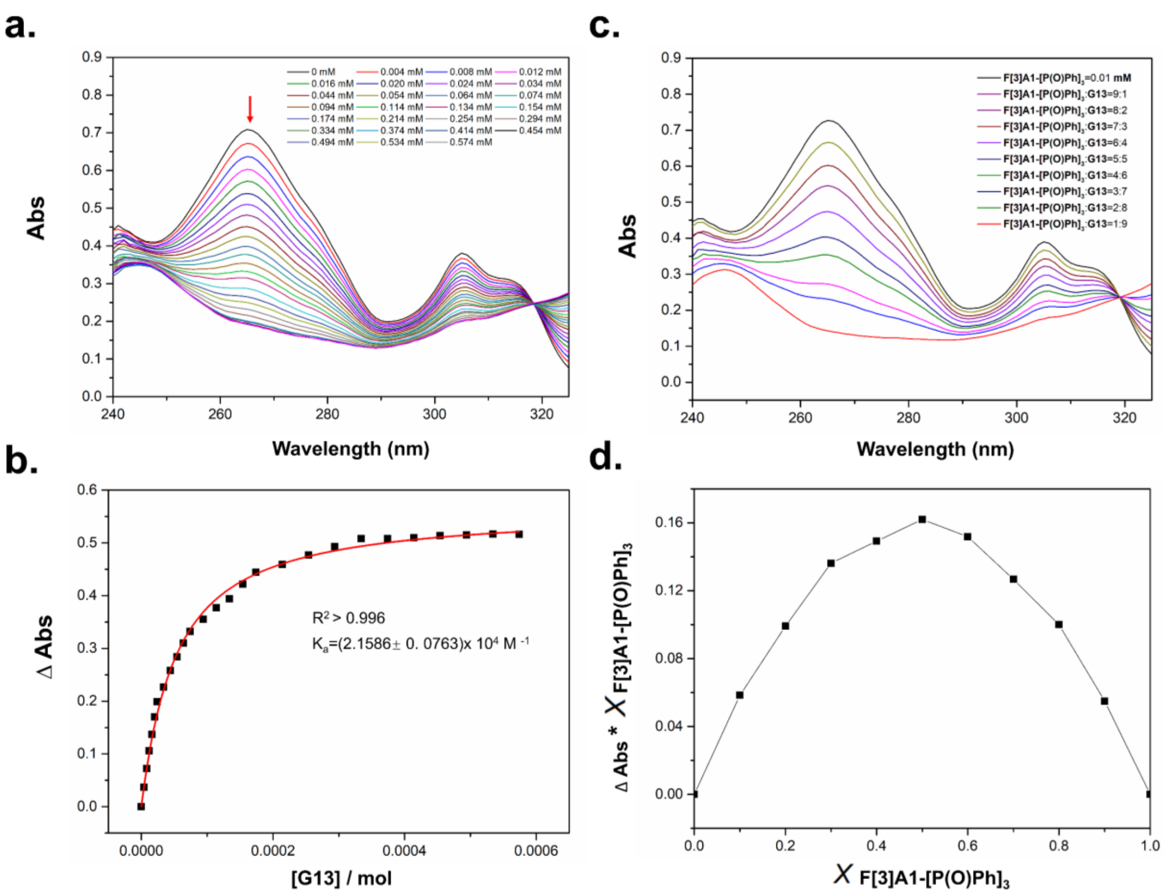

Supplementary Figure 56. UV–Vis titration experiments of F[3]A1-[P(O)Ph]$_3$ with G13 (1,4-bis(4-cyanostyryl)benzene). (a). **UV–Vis** absorption spectra of **F[3]A1-[P(O)Ph]$_3$** at a constant concentration of 0.01 mM with different concentrations of **G13** (1,4-bis(4-cyanostyryl)benzene), ranging from 0 mM to 0.574 mM. (b). The absorbance changes of **F[3]A1-[P(O)Ph]$_3$** upon the addition of **G13**. The red solid line was obtained from the non-linear curve fitting. The association constant (K_a) between **F[3]A1-[P(O)Ph]$_3$** and **G13** was estimated to be (2.1586 ± 0.0763) × 10^4 M^{-1}. (c). **UV-Vis** absorption spectra of complex **F[3]A1-[P(O)Ph]$_3$ @ G13** with different molar ratios in water while [**F[3]A1-[P(O)Ph]$_3$**] + [**G13**] = 0.10 mM. (d). Job plots of the complex **F[3]A1-[P(O)Ph]$_3$ @ G13** showing a 1:1 stoichiometry between **F[3]A1-[P(O)Ph]$_3$** and **G13** by plotting the absorbance differences at 265 nm (a characteristic absorption peak of **F[3]A1-[P(O)Ph]$_3$**) against the mole fraction of **G13**.

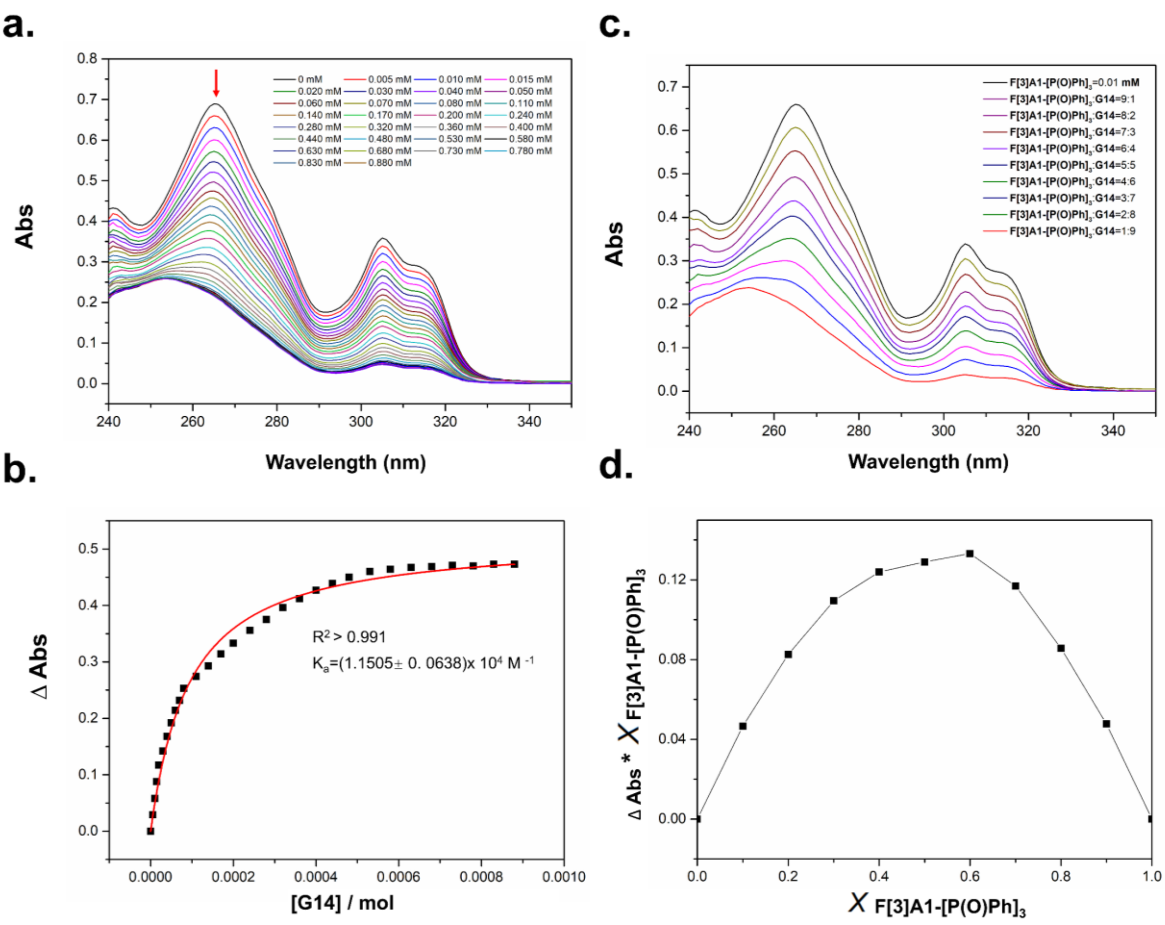

Supplementary Figure 57. UV–Vis titration experiments of F[3]A1-[P(O)Ph]3 with G14 (crisaborole). (a). **UV–Vis** absorption spectra of **F[3]A1-[P(O)Ph]3** at a constant concentration of 0.01 mM with different concentrations of **G14** (crisaborole), ranging from 0 mM to 0.88 mM. (b). The absorbance changes of **F[3]A1-[P(O)Ph]3** upon the addition of **G14**. The red solid line was obtained from the non-linear curve fitting. The association constant (***K**a*) between **F[3]A1-[P(O)Ph]3** and **G14** was estimated to be (1.1505 ± 0.0638) × 10^4 M^{-1}. (c). **UV-Vis** absorption spectra of complex **F[3]A1-[P(O)Ph]3** @ **G14** with different molar ratios in water while [**F[3]A1-[P(O)Ph]3**] + [**G14**] = 0.10 mM. (d). Job plots of the complex **F[3]A1-[P(O)Ph]3** @ **G14** showing a 1:1 stoichiometry between **F[3]A1-[P(O)Ph]3** and **G14** by plotting the absorbance differences at 265 nm (a characteristic absorption peak of **F[3]A1-[P(O)Ph]3**) against the mole fraction of **G14**.

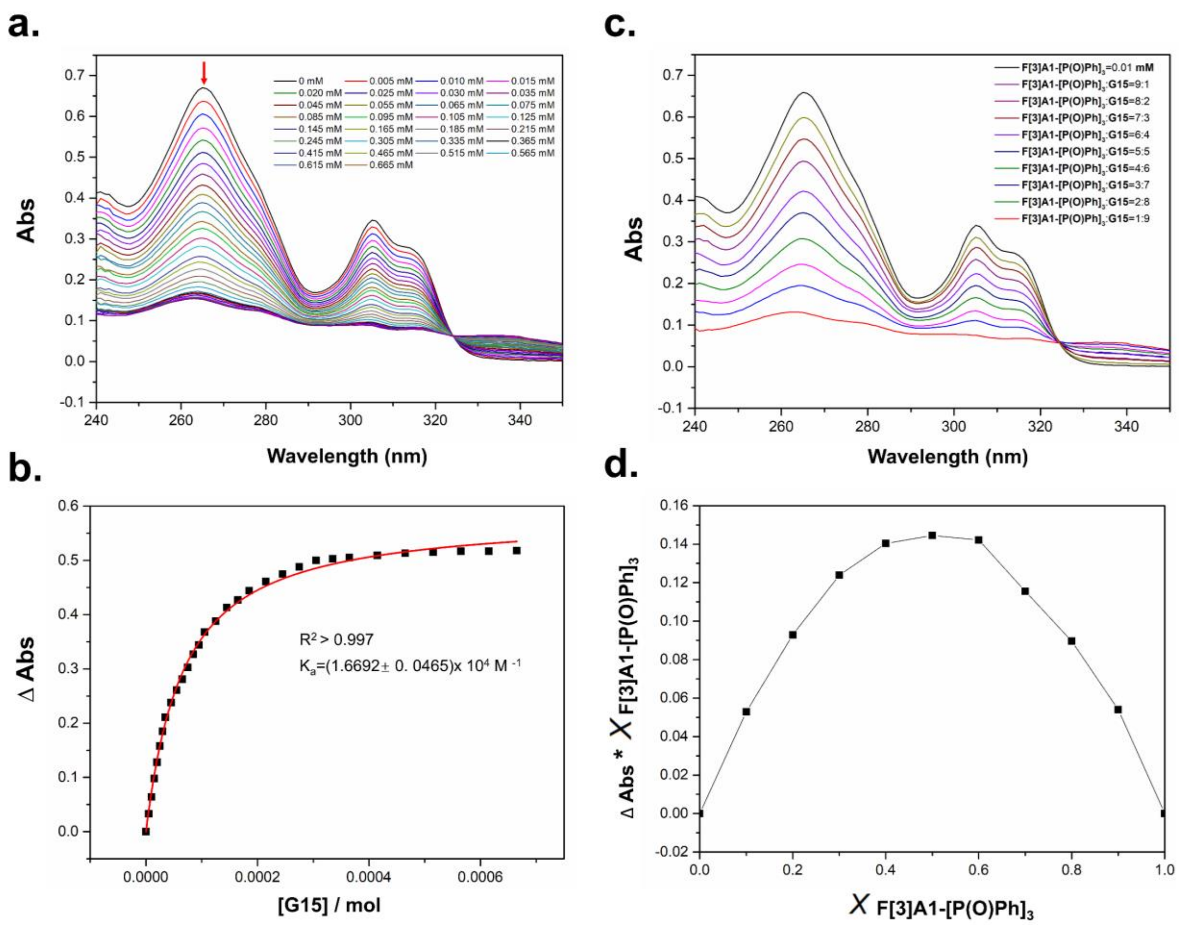

Supplementary Figure 58. UV–Vis titration experiments of F[3]A1-[P(O)Ph]$_3$ with G15 (alectinib). (a). **UV–Vis** absorption spectra of **F[3]A1-[P(O)Ph]$_3$** at a constant concentration of 0.01 mM with different concentrations of **G15** (alectinib), ranging from 0 mM to 0.665 mM. (b). The absorbance changes of **F[3]A1-[P(O)Ph]$_3$** upon the addition of **G15**. The red solid line was obtained from the non-linear curve fitting. The association constant (K_a) between **F[3]A1-[P(O)Ph]$_3$** and **G15** was estimated to be (1.6692 ± 0.0465) × 10^4 M^{-1}. (c). **UV-Vis** absorption spectra of complex **F[3]A1-[P(O)Ph]$_3$ @ G15** with different molar ratios in water while [**F[3]A1-[P(O)Ph]$_3$**] + [**G15**] = 0.10 mM. (d). Job plots of the complex **F[3]A1-[P(O)Ph]$_3$ @ G15** showing a 1:1 stoichiometry between **F[3]A1-[P(O)Ph]$_3$** and **G15** by plotting the absorbance differences at 265 nm (a characteristic absorption peak of **F[3]A1-[P(O)Ph]$_3$**) against the mole fraction of **G15**.

2.9. 2D Hirschfeld fingerprinting figure

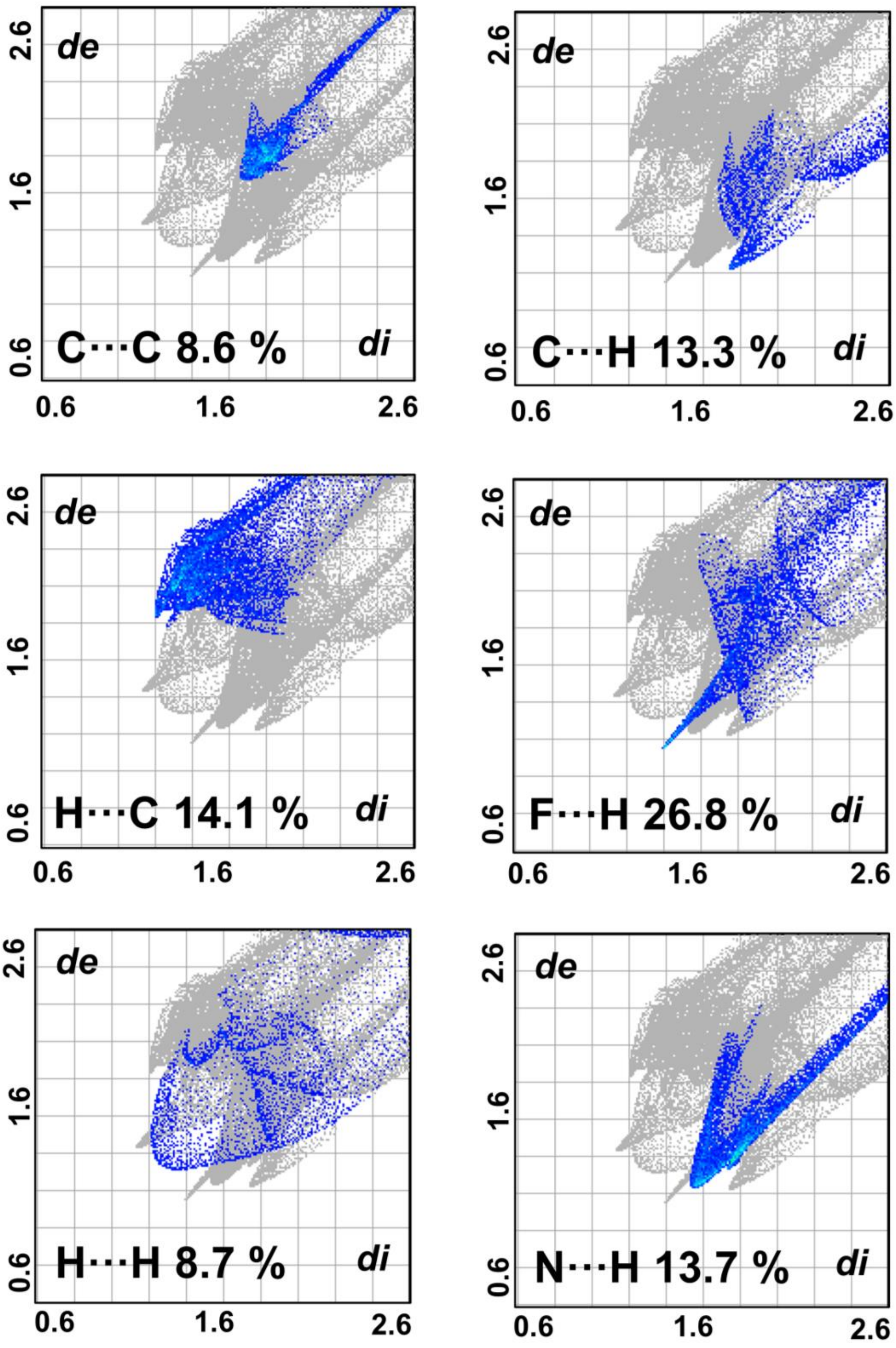

Supplementary Figure 59. The **2D Hirschfeld** fingerprinting of 3,4-difluorobenzonitrile (**G2**)

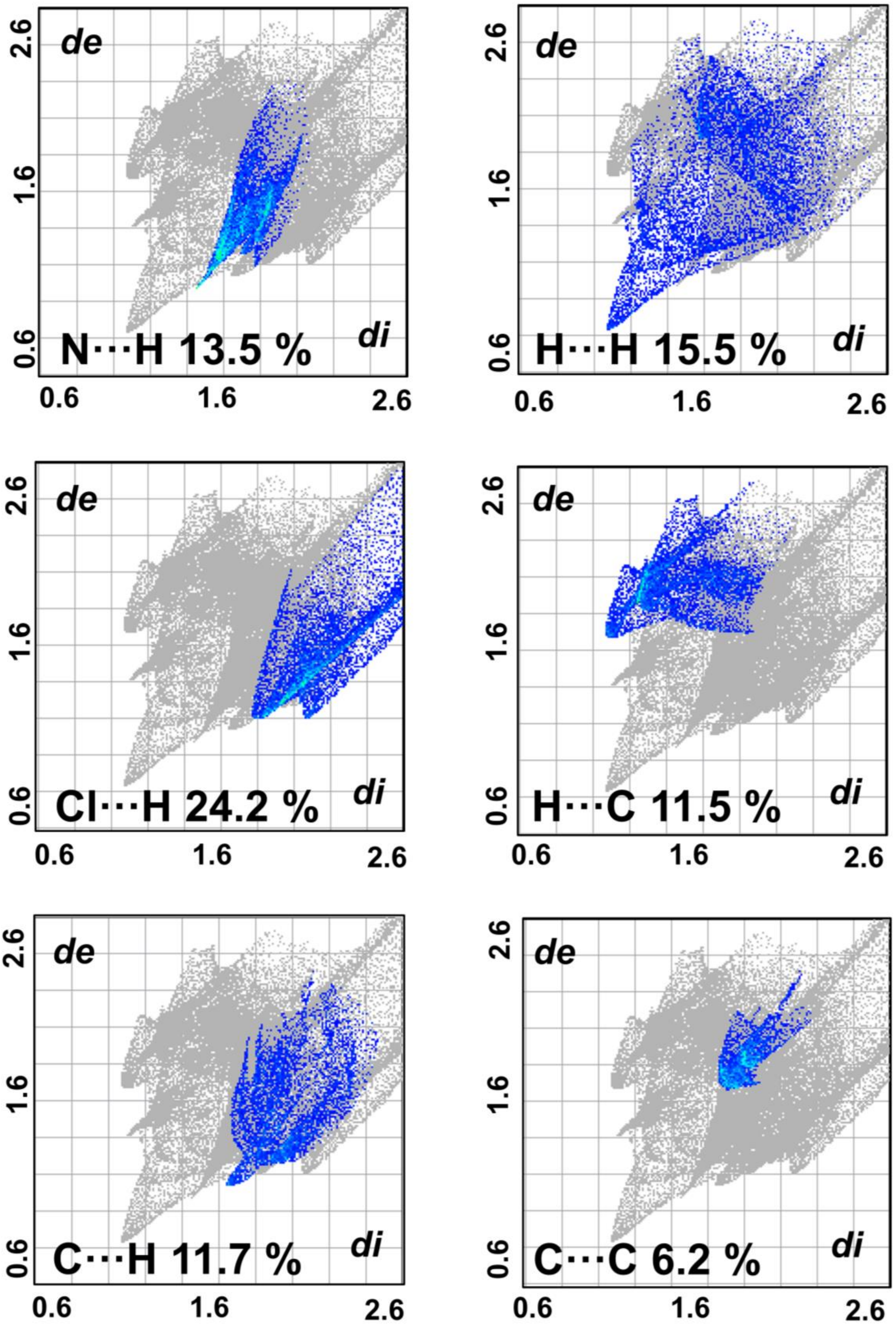

Supplementary Figure 60. The **2D Hirschfeld** fingerprinting of 4-chlorobenzonitrile (**G3**)

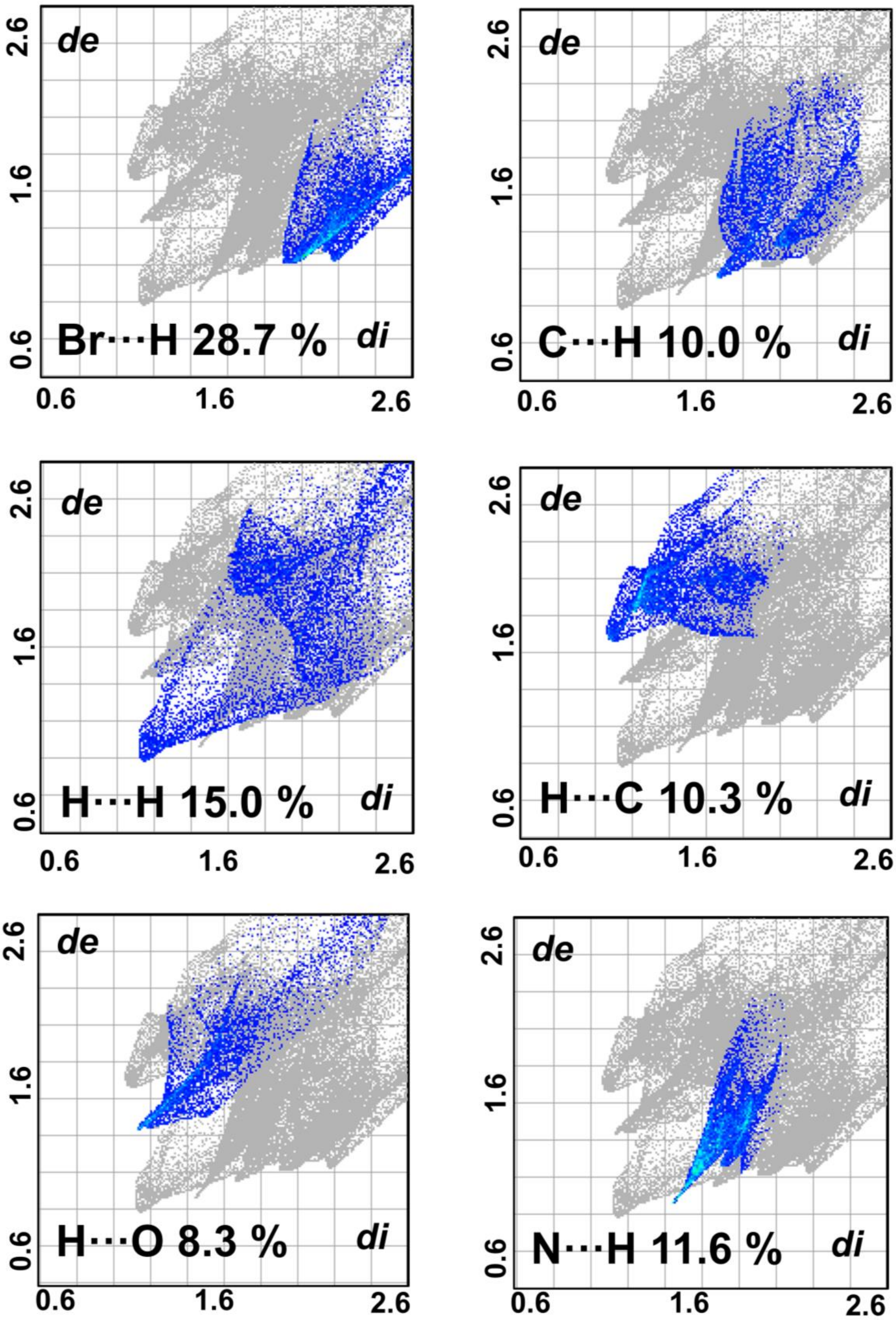

Supplementary Figure 61. The **2D Hirschfeld** fingerprinting of 4-bromobenzonitrile (**G4**)

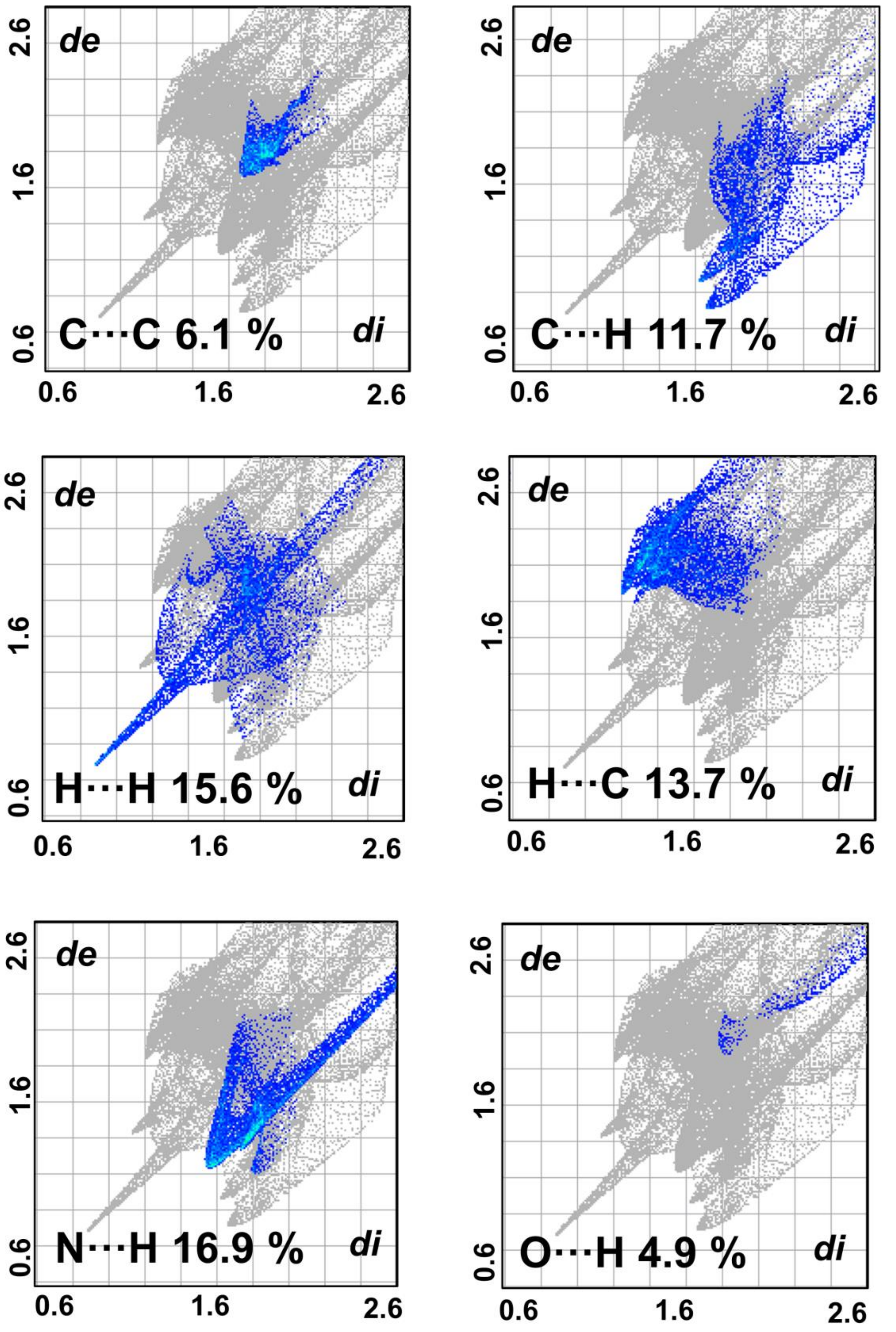

Supplementary Figure 62. The **2D Hirschfeld** fingerprinting of 4-fluoro-3-hydroxybenzonitrile (**G5**)

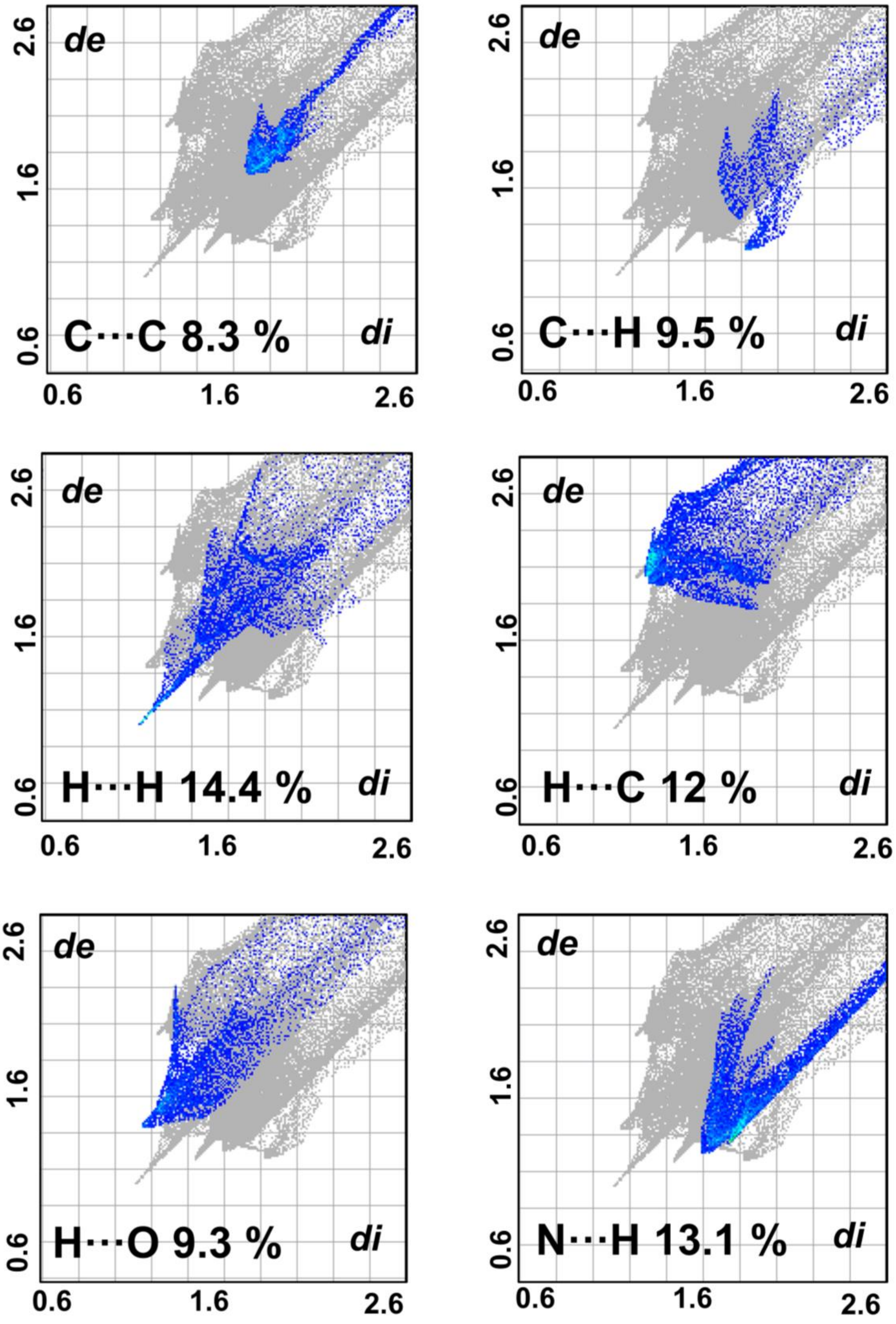

Supplementary Figure 63. The **2D Hirschfeld** fingerprinting of 4-trifluoromethylbenzonitrile (**G6**)

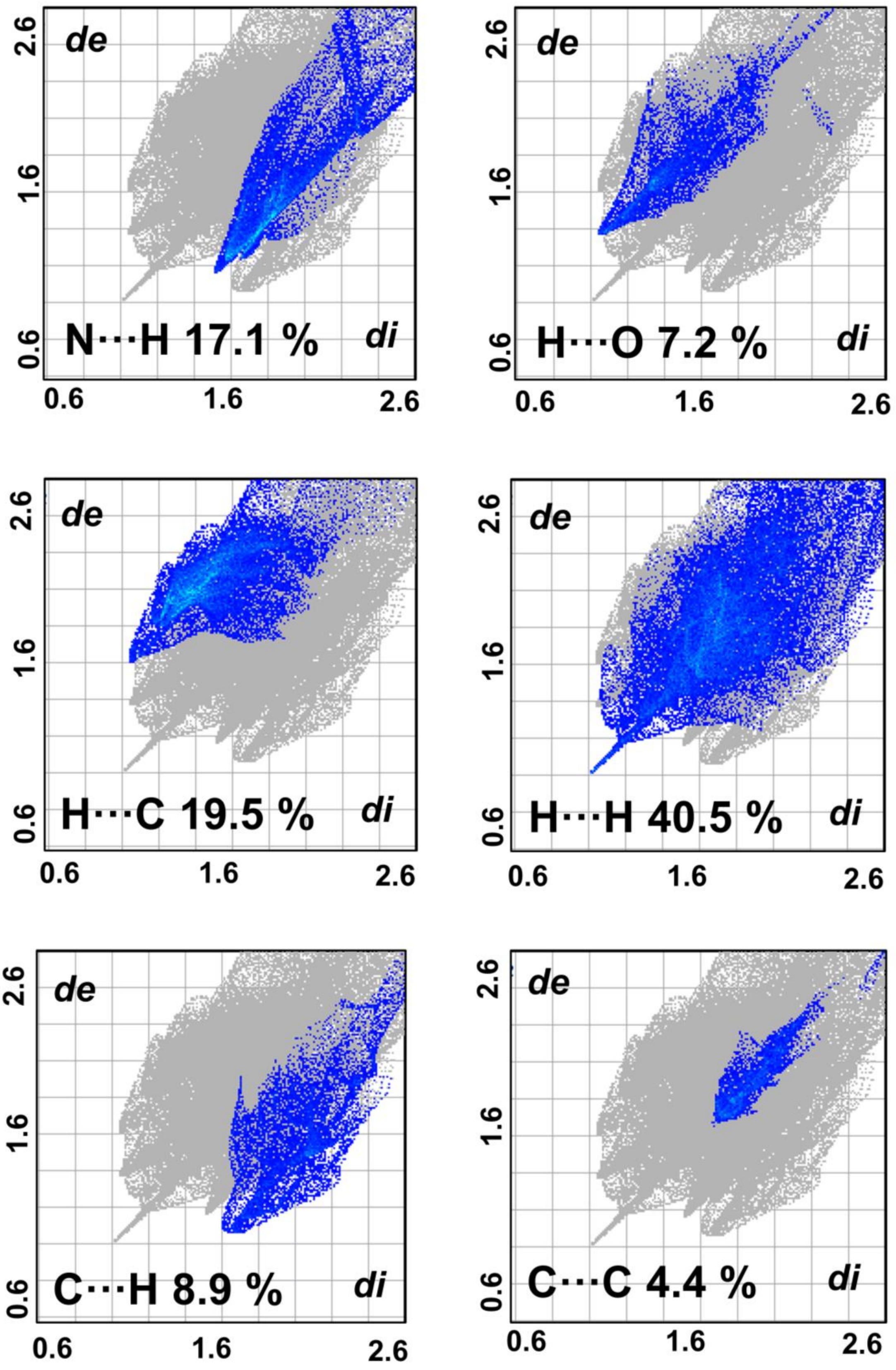

Supplementary Figure 64. The **2D Hirschfeld** fingerprinting of 4-ethylbenzonitrile (**G7**)

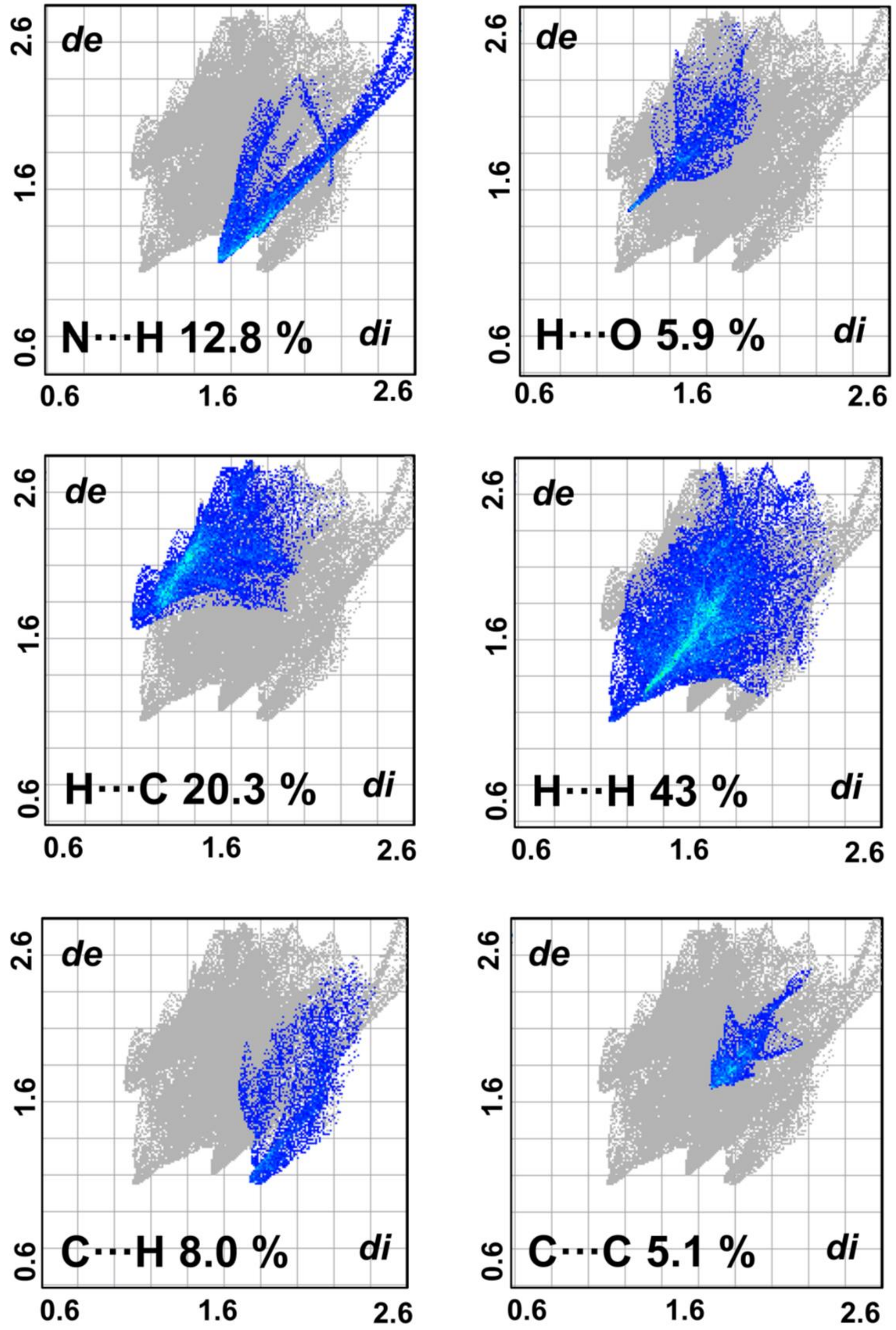

Supplementary Figure 65. The **2D Hirschfeld** fingerprinting of 4-butylbenzonitrile (**G8**)

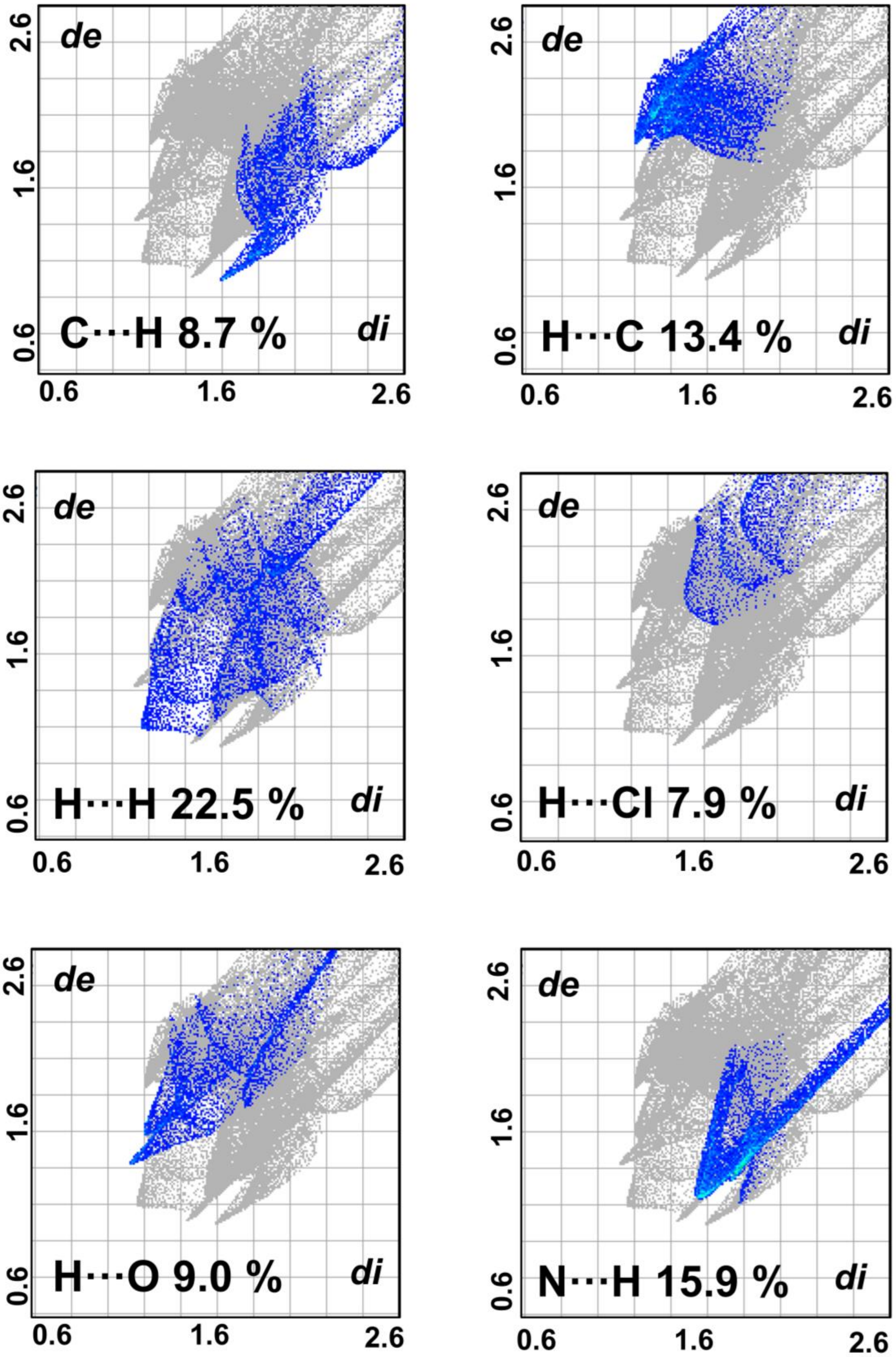

Supplementary Figure 66. The **2D Hirschfeld** fingerprinting of 3,4-dihydroxybenzonitrile (**G9**)

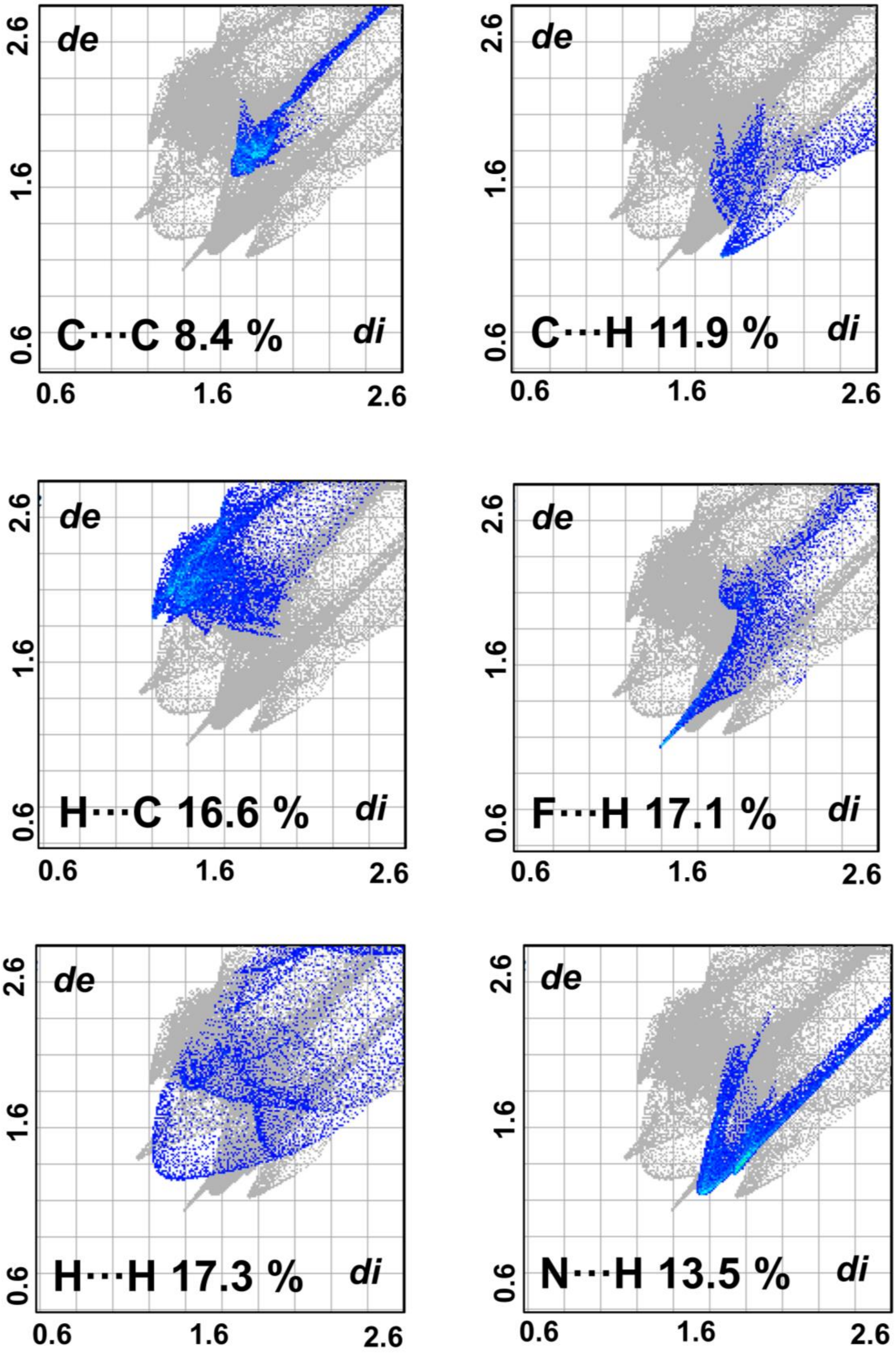

Supplementary Figure 67. The **2D Hirschfeld** fingerprinting of 3-fluoro-4-hydroxybenzonitrile (**G10**)

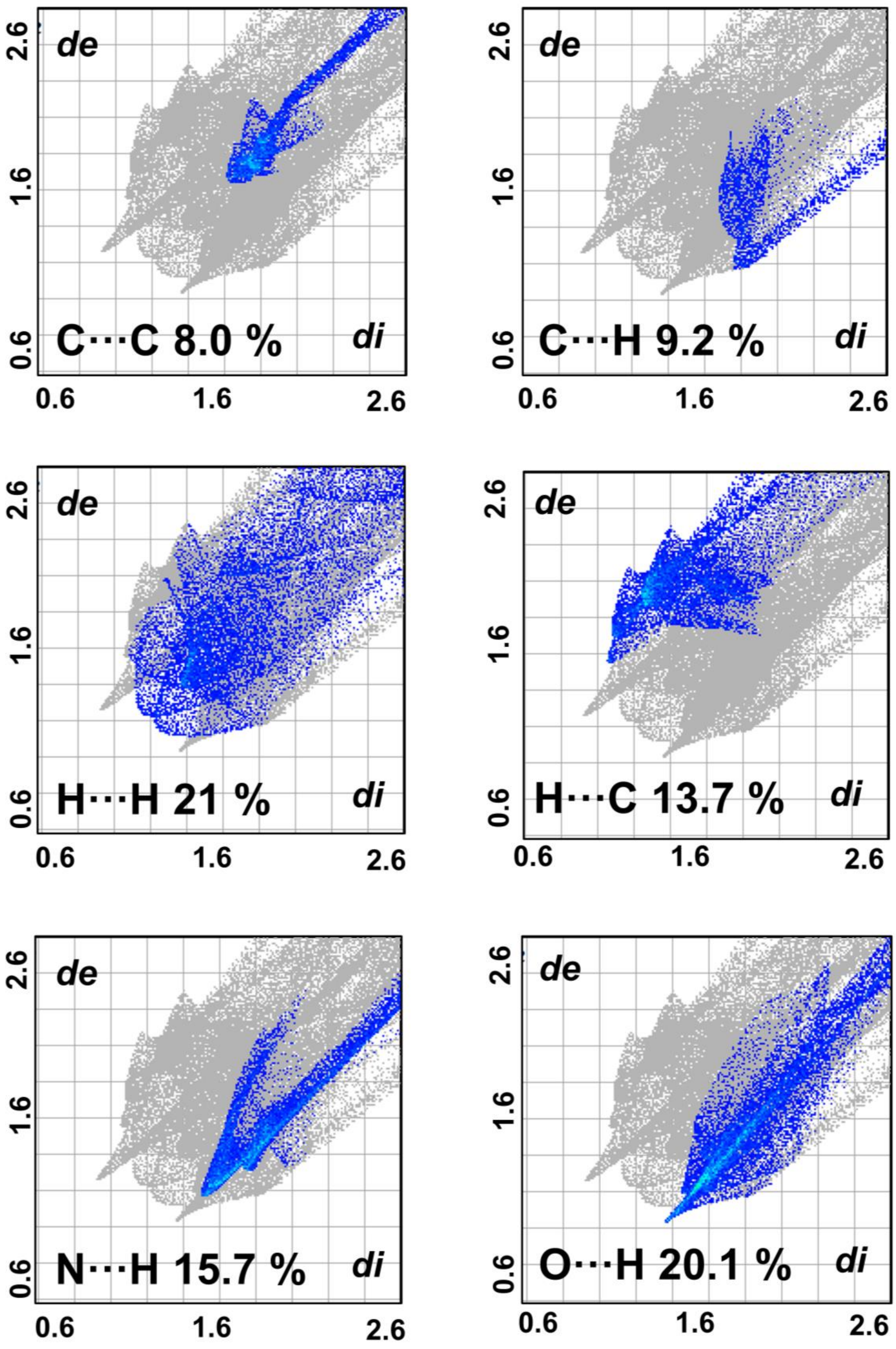

Supplementary Figure 68. The **2D Hirschfeld** fingerprinting of 4-cyanobenzenesulfonamide (**G11**)

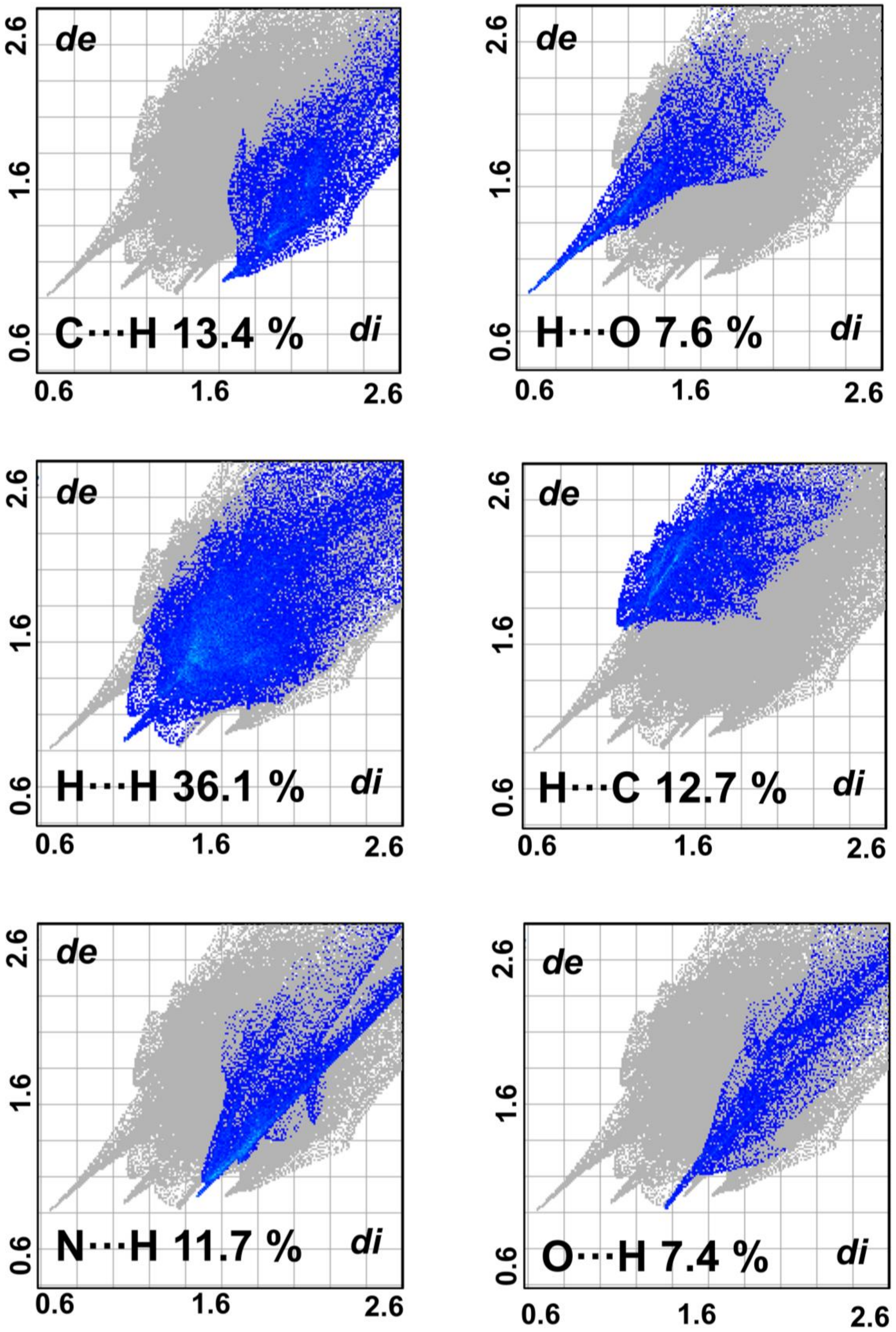

Supplementary Figure 69. The **2D Hirschfeld** fingerprinting of 4'-Hydroxy-4-biphenylcarbonitrile (**G12**)

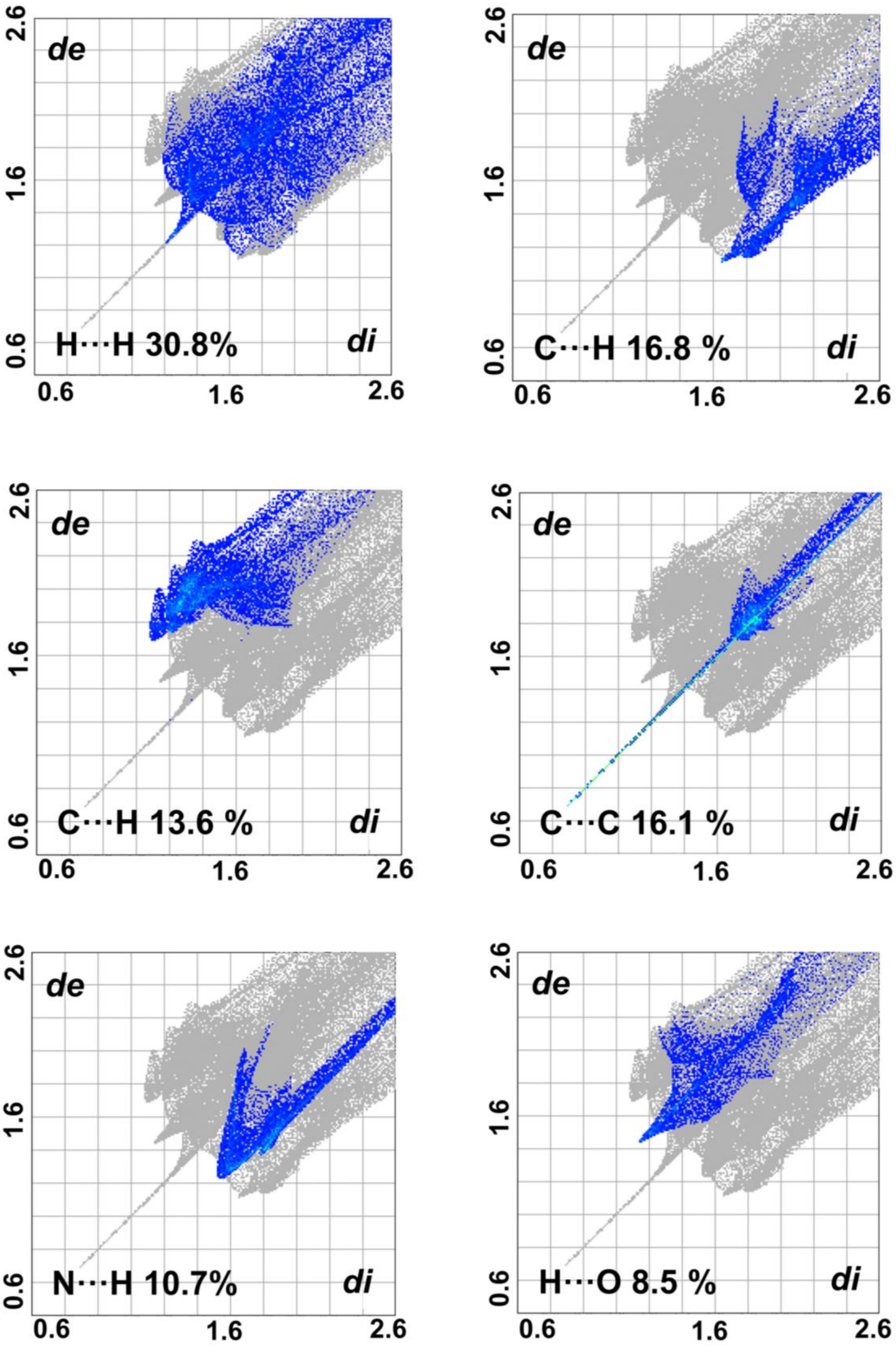

Supplementary Figure 70. The **2D Hirschfeld** fingerprinting of 1,4-bis(4-cyanostyryl)benzene (**G13**)

2.10. Electron cloud density map

Guest molecule	OPTEP (50% probability)	F_o map	Data quality *
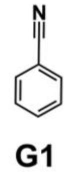 G1	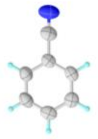	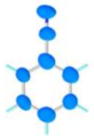	(1) 0.0523 (2) 0.1288<br>(3) 1.052 (4) 100%<br>(5) 5.0
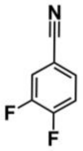 G2	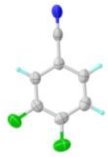	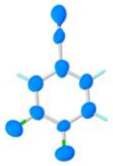	(1) 0.0758 (2) 0.2158<br>(3) 1.033 (4) 100%<br>(5) 5.0
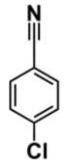 G3	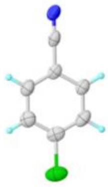	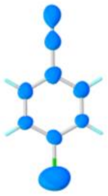	(1) 0.0689 (2) 0.1923<br>(3) 1.069 (4) 100%<br>(5) 4.9
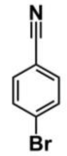 G4	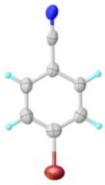	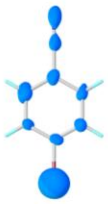	(1) 0.0724 (2) 0.1976<br>(3) 1.082 (4) 100%<br>(5) 4.9
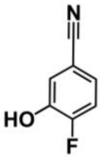 G5	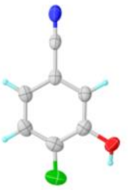	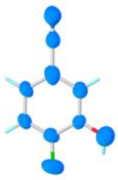	(1) 0.0807 (2) 0.2302<br>(3) 1.019 (4) 100%<br>(5) 1 (6) 4.9
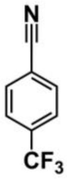 G6	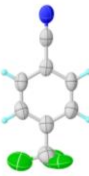	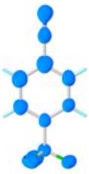	(1) 0.0835 (2) 0.2490<br>(3) 1.056 (4) 100%<br>(5) 4.9
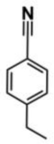 G7	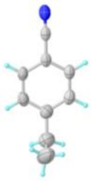	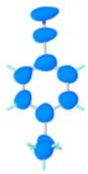	(1) 0.0567 (2) 0.1618<br>(3) 1.076 (4) 100%<br>(5) 5.0

Guest molecule	OPTEP (50% probability)	F_o map	Data quality *
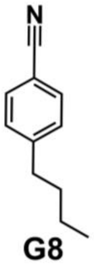 G8	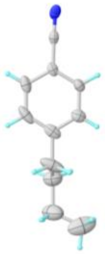	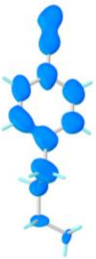	(1) 0.0631 (2) 0.1672 (3) 1.027 (4) 100% (5) 5.0
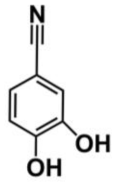 G9	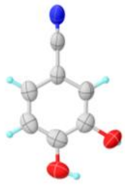	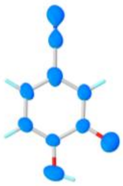	(1) 0.0894 (2) 0.2553 (3) 1.033 (4) 100% (5) 4.8
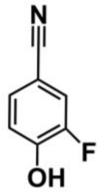 G10	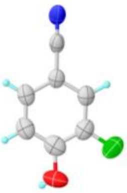	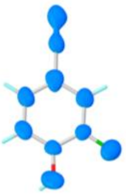	(1) 0.0892 (2) 0.2679 (3) 1.037 (4) 100% (5) 5.0
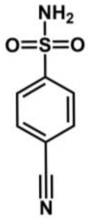 G11	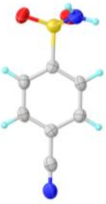	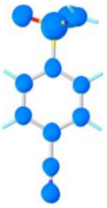	(1) 0.0664 (2) 0.1827 (3) 1.017 (4) 100% (5) 5.0
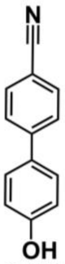 G12	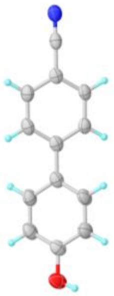	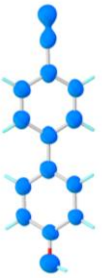	(1) 0.0458 (2) 0.1286 (3) 1.062 (4) 100% (5) 5.1

Guest molecule	OPTEP (50% probability)	F_o map	Data quality *
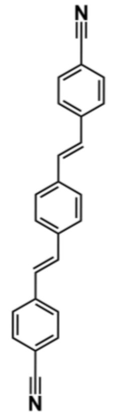 G13	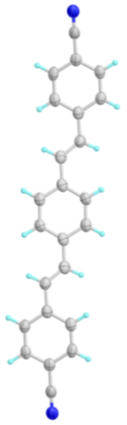	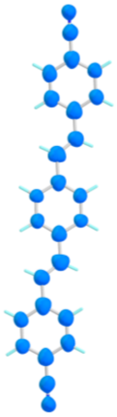	(1) 0.0834 (2) 0.2493 (3) 1.052 (4) 100% (5) 4.9
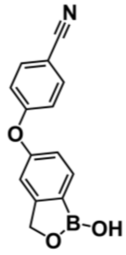 G14	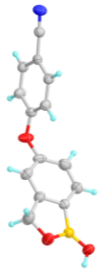	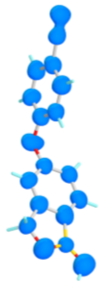	(1) 0.0479 (2) 0.1197 (3) 1.065 (4) 100% (5) 5.0
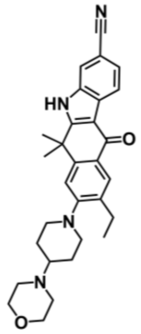 G15	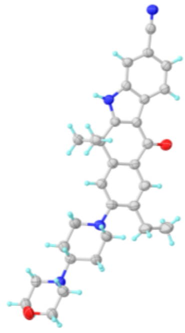	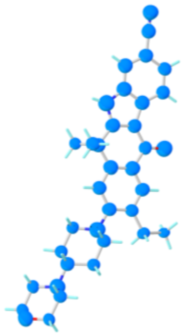	(1)0.0832 (2) 0.2576 (3) 1.048 (4) 100% (5) 5.0

Supplementary Figure 71. Structures and electron densities of 15 guest molecules by **F[3]A1-[P(O)Ph]$_3$** for molecular docking by co-crystallization. Data quality* (1) $R_1[F_2 > 2\sigma(F_2)]$; (2) wR_2;(3) Goodness-of-fit; (4) Occupany of guest molecules; (5) The level of plotted isosurface for **Fo** electron density map (**ORTEP** plots at 50% probability).

2.11. Details of DFT calculations

Supplementary Table 2. Coordinates of the fully optimized structure of **F[3]A1-[P(O)Ph]$_3$**. The **B3LYP** [3] density functional method with the **D3**(**BJ**) [4] dispersion correction was employed in this work to carry out all the computations. The **6-31G**(**d**) [3] basis set was used for the atoms in geometry optimizations using the **PCM** model with dichloroethane as the solvent. Vibrational frequency analyses at the same level of theory were performed to characterize stationary points as local minima without any imaginary frequencies.

P -1.56312623 1.63326651 0.00000000
P 3.59987377 11.53626651 2.81800000
P -0.11812623 4.56126651 10.69700000
O 3.95787377 10.55926651 1.61400000
O -0.31212623 2.29726651 -0.73000000
O 4.54387377 11.14026651 4.03000000
O -0.94012623 0.61826651 1.05400000
O 0.19687377 3.07326651 10.23700000
O 1.31187377 5.25326651 10.87500000
O 2.17387377 11.56126651 3.14700000
O -2.52712623 2.56626651 0.56700000
O -1.02112623 5.30626651 9.82700000
C 2.24287377 4.10126651 1.20000000
H 2.94887377 3.92426651 1.81000000
C 2.63587377 6.65926651 1.14000000
C 1.99187377 5.39826651 0.78300000
C 4.40387377 7.76226651 5.47000000
H 4.69787377 6.87926651 5.28200000
C 1.45287377 3.05426651 0.72000000
C 1.68487377 2.57426651 6.91000000
H 2.51187377 2.68526651 6.45600000
C 3.69387377 6.91326651 2.00500000
H 4.10987377 6.19226651 2.46400000
C 3.23587377 7.12226651 7.70900000
C 0.43387377 3.35126651 -0.16500000
C 3.47987377 9.23926651 1.50300000
C 4.14687377 8.21026651 2.20400000
C 3.72287377 8.03326651 6.65400000
C 1.99887377 7.70426651 0.46700000
C 1.56687377 2.94326651 8.24800000
C -2.14012623 0.50526651 -1.24400000
C 0.94687377 5.67026651 -0.12700000
C 3.32587377 5.76026651 7.82700000
H 3.82487377 5.26026651 7.19100000
C 2.41787377 9.01126651 0.64600000
H 1.98887377 9.73026651 0.19400000
C -0.91512623 1.22526651 4.65700000
C 0.40287377 1.66626651 4.83500000
C 1.27987377 1.74026651 3.75400000
H 2.18287377 2.00526651 3.88600000
C 1.69687377 1.65126651 1.25600000
H 1.48287377 0.98326651 0.55800000
H 2.65087377 1.54626651 1.50400000
C 0.58687377 2.04326651 6.23900000
C 3.56287377 10.37226651 6.08700000
H 3.30087377 11.26226651 6.29100000
C 0.33287377 2.74126651 8.87300000
C 4.65287377 8.79626651 4.55600000
C 1.85287377 7.25826651 9.68200000
H 1.35487377 7.75726651 10.32000000
C -0.49312623 0.99526651 2.33700000
C 2.67987377 5.09526651 8.89000000
C 2.71787377 3.58826651 8.97700000
H 3.56987377 3.26326651 8.59000000
H 2.69287377 3.31726651 9.92800000
C 3.29287377 9.33526651 6.94900000
C -1.36512623 0.86426651 3.40200000
H -2.24712623 0.53526651 3.27500000
C 4.23487377 10.07426651 4.90600000
C 0.81887377 1.42326651 2.47700000
C 5.24687377 8.49726651 3.19500000
H 5.77887377 9.27226651 2.88600000
H 5.84887377 7.71526651 3.25800000
C 0.16287377 4.64626651 -0.61200000
H -0.53912623 4.81526651 -1.22900000
C 1.95787377 5.87926651 9.78100000
C 2.49087377 7.88626651 8.63300000
C -0.76212623 2.18026651 8.24000000
H -1.57612623 2.03326651 8.70800000
C 0.88487377 7.15726651 -0.41700000
H 0.00487377 7.53726651 -0.16700000
H 1.05887377 7.34526651 -1.37400000
C -1.67212623 1.28726651 5.95700000
H -1.97312623 0.38526651 6.24200000
H -2.45812623 1.88326651 5.88800000
C -0.65912623 4.31126651 12.36700000
C 4.34687377 13.04326651 2.25300000
C 2.50087377 9.34126651 8.23200000
H 1.58287377 9.67826651 8.08200000
H 2.94287377 9.89926651 8.92100000
C -0.63912623 1.84526651 6.91100000
C -1.21512623 -0.26173349 -1.99600000
H -0.28112623 -0.18073349 -1.83700000
C -3.48912623 0.34526651 -1.46000000
H -4.11012623 0.83526651 -0.93500000
C -3.94912623 -0.52173349 -2.43700000
H -4.88312623 -0.61273349 -2.58700000
C -0.02212623 3.43126651 13.20400000
H 0.74787377 2.96726651 12.90000000
C -3.06012623 -1.24973349 -3.18700000
H -3.37812623 -1.83573349 -3.86400000
C -1.69012623 -1.13373349 -2.95900000
H -1.08112623 -1.65473349 -3.46800000
C -1.80612623 4.96726651 12.80400000
H -2.25512623 5.57126651 12.22700000
C -0.48812623 3.21026651 14.49300000
H -0.03112623 2.62126651 15.08100000
C -1.64412623 3.87426651 14.90100000
H -1.98812623 3.71926651 15.77300000
C 4.98487377 13.90826651 3.10400000
H 5.04887377 13.70026651 4.02800000
C 4.25987377 13.37626651 0.90400000
H 3.84387377 12.77726651 0.29600000
C -2.28812623 4.74126651 14.06800000
H -3.07012623 5.18826651 14.36400000
C 4.77687377 14.57726651 0.43800000
H 4.66787377 14.82126651 -0.47300000
C 5.44087377 15.40126651 1.29300000
H 5.83787377 16.20026651 0.96800000
C 5.53787377 15.08126651 2.61900000
H 5.98687377 15.67126651 3.21400000

Supplementary Table 3. Coordinates of the fully optimized structure of benzonitrile @ **F[3]A1-[P(O)Ph]$_3$**.

P 3.21700000 -0.43100000 16.46200000
P -1.73500000 2.16600000 7.12400000
P 6.37000000 9.06600000 10.81400000
O -2.67000000 1.72600000 8.34000000
O 3.69000000 0.92800000 17.14000000
O -1.91100000 3.74100000 6.99600000
O 3.70800000 -0.62800000 15.09700000
O 6.20100000 8.88800000 12.38500000
O 1.62100000 -0.44500000 16.58800000
O -0.34800000 1.71500000 7.25200000
O 4.97100000 9.55200000 10.26100000
O 6.93300000 7.88900000 10.15000000
C 0.49800000 6.41100000 9.07000000
C 3.98200000 4.52500000 15.09400000
C -0.02700000 5.21700000 7.03800000
H 0.13700000 5.01400000 6.12400000
C 3.68900000 -1.64200000 17.66900000
C 0.77900000 6.10600000 7.72500000
C -0.58600000 5.83600000 9.72300000
H -0.77900000 6.07100000 10.62200000
C -1.39000000 4.90900000 9.05200000
C -0.09500000 2.07700000 14.53300000
H -0.26700000 3.01200000 14.51700000
C 4.36100000 5.72300000 14.33700000
C -1.61700000 2.82700000 11.69500000
H -1.54700000 3.61800000 12.21600000
C 3.58700000 6.76500000 13.84900000
H 2.64800000 6.76500000 13.99200000
C 4.18300000 7.81400000 13.14900000
C 2.61800000 2.89400000 16.22800000
C -2.17100000 1.70300000 9.66100000
C -0.35000000 -0.12100000 13.53200000
C 5.56700000 7.77500000 12.98000000
C -0.57200000 1.26700000 13.50500000
C 0.63500000 1.51800000 15.58600000
C 5.75000000 5.69300000 14.11900000
C -2.53500000 4.21500000 9.77500000
H -2.87600000 4.81000000 10.49100000
H -3.27400000 4.05500000 9.13600000
C -1.22300000 1.60500000 12.23200000
C -1.80300000 0.46800000 10.17200000
H -1.85500000 -0.31900000 9.64100000
C 5.13200000 3.76600000 15.33500000
C 5.03800000 2.55700000 16.01500000
H 5.80900000 2.03000000 16.19000000
C -2.11500000 2.89100000 10.38800000
C 2.73600000 4.10700000 15.55100000
H 1.96800000 4.64700000 15.40100000
C -1.08000000 4.63100000 7.71900000
C 2.96200000 8.51700000 11.11000000
C -0.91300000 -0.77100000 12.29500000
H -1.67500000 -1.36200000 12.51400000
H -0.22100000 -1.29500000 11.81700000
C 1.79800000 7.79700000 10.84700000
H 1.17100000 7.64200000 11.54400000
C -1.35800000 0.41900000 11.47900000
C 1.55000000 7.30500000 9.56800000
C 3.78000000 2.14700000 16.43000000
C 6.36300000 6.73800000 13.44000000
H 7.30200000 6.74100000 13.29500000
C 6.34200000 4.44300000 14.73600000
H 6.77000000 3.87200000 14.04900000
H 7.01000000 4.66700000 15.43300000
C 0.36300000 -0.69400000 14.56700000
H 0.51100000 -1.63300000 14.59900000
C 0.85800000 0.14300000 15.55900000
C 2.02600000 6.82200000 7.26900000
H 1.82900000 7.45900000 6.53800000
H 2.71800000 6.18200000 6.96600000
C 5.05400000 -1.92500000 17.78900000
H 5.68800000 -1.44500000 17.27000000
C -2.66800000 1.55900000 5.73700000
C 7.29200000 10.58500000 10.69700000
C 2.46100000 7.54500000 8.52400000
C 3.33400000 8.90700000 12.52900000
H 3.83900000 9.76000000 12.52000000
H 2.51300000 9.03800000 13.06500000
C 3.83700000 8.74200000 10.04200000
C 1.25200000 2.39500000 16.65300000
H 1.33800000 1.87900000 17.49400000
H 0.66000000 3.16800000 16.82800000
C 3.61400000 8.27800000 8.75300000
H 4.23100000 8.45700000 8.05300000
C -2.78500000 0.17600000 5.58600000
H -2.41200000 -0.40800000 6.23700000
C 2.76100000 -2.33500000 18.44400000
H 1.83600000 -2.13500000 18.37400000
C 8.68300000 10.53400000 10.61100000
H 9.13600000 9.70000000 10.65600000
C 5.47400000 -2.90900000 18.66900000
H 6.40000000 -3.10800000 18.75200000
C 4.54800000 -3.60000000 19.42300000
H 4.84000000 -4.27600000 20.02300000
C -3.98400000 0.49400000 3.53200000
H -4.43000000 0.13300000 2.77600000
C -3.44900000 -0.34400000 4.48000000
H -3.53300000 -1.28500000 4.37900000
C 3.19700000 -3.31700000 19.31400000
H 2.56900000 -3.79800000 19.84000000
C -3.21800000 2.40300000 4.78100000
H -3.14800000 3.34500000 4.87800000
C 6.64600000 11.81300000 10.66500000
H 5.70000000 11.85600000 10.74400000
C -3.87300000 1.85900000 3.68100000
H -4.24700000 2.43500000 3.02400000
C 7.38100000 12.97600000 10.51900000
H 6.93700000 13.81600000 10.48700000
C 9.39500000 11.71400000 10.46000000
H 10.34100000 11.68800000 10.38400000
C 8.74500000 12.91900000 10.41900000
H 9.24500000 13.72200000 10.32100000
N 0.89400000 4.86500000 12.86700000
C 1.53100000 4.55100000 11.98000000
C 3.53000000 4.83000000 10.58200000
H 3.90300000 5.41200000 11.23300000
C 2.30400000 4.20500000 10.80700000
C 1.75600000 3.31800000 9.86500000
H 0.92200000 2.89500000 10.03200000
C 3.64100000 3.70000000 8.46000000
H 4.10300000 3.53200000 7.64700000
C 4.19100000 4.58600000 9.39400000
H 5.01700000 5.01800000 9.21000000
C 2.44200000 3.06900000 8.69900000
H 2.08900000 2.46200000 8.05900000

Supplementary Table 4. Coordinates of the fully optimized structure of *p*-fluorotoluene @ **F[3]A1-[P(O)Ph]$_3$**.

P 0.75187970 1.33458645 0.00000000
P 3.17487970 2.31258645 -10.75400000
P 1.09987970 -7.91741355 -6.43100000
O 0.02087970 2.46558645 -0.84400000
O 2.13787970 3.29858645 -10.06900000
O -0.03012030 -7.71041355 -5.34600000
O -0.25712030 0.12158645 0.10000000
O 2.33287970 1.16258645 -11.42900000
O 0.45587970 -7.48941355 -7.81100000
O 2.36387970 -7.25441355 -6.13300000
O 2.07687970 0.97358645 -0.49700000
O 4.20387970 1.83558645 -9.83700000
C -0.61312030 -4.03441355 -3.56000000
C -0.84112030 1.69258645 -2.94000000
C -0.22212030 -6.44741355 -4.73900000
C 0.18787970 -1.00641355 -9.41500000
H -0.60712030 -0.96341355 -8.89700000
C -0.04912030 2.00558645 -7.44500000
H -0.79712030 1.43058645 -7.33800000
C 0.67887970 0.13858645 -10.03000000
C -0.49012030 -3.96641355 -8.22200000
H -1.17312030 -3.36041355 -7.95800000
C 0.16387970 -5.00941355 -2.92000000
C -1.20412030 -4.28241355 -4.77100000
H -1.74512030 -3.61741355 -5.18100000
C 0.48487970 -6.13341355 -8.21400000
C 0.54187970 -3.53441355 -9.03800000
C -0.52712030 -5.28741355 -7.78800000
C -0.62812030 -2.83541355 -2.73600000
C 0.06087970 2.48358645 -2.24000000
C 0.49287970 2.64358645 -6.35200000
C -0.35312030 -0.86741355 -0.88700000
C 0.12787970 -3.07841355 -1.59100000
C 0.85587970 -2.20941355 -9.55800000
C 0.36187970 -6.23841355 -3.51200000
H 0.87987970 -6.91241355 -3.08900000
C 0.50087970 2.20758645 -8.70000000
C 0.15187970 2.52658645 -4.94900000
C -0.79012030 1.73158645 -4.31700000
H -1.39812030 1.21458645 -4.83100000
C 1.86287970 0.02558645 -10.74400000
C -0.02812030 1.46058645 -9.88400000
H 0.10487970 1.99958645 -10.70300000
H -1.00012030 1.30658645 -9.77200000
C 1.54087970 -4.42341355 -9.45300000
C -1.24612030 -1.61441355 -2.94800000
H -1.75512030 -1.47141355 -3.73700000
C -1.01312030 -5.50641355 -5.40000000
C 2.14687970 3.72158645 -7.72500000
H 2.88987970 4.30358645 -7.83300000
C 1.03287970 3.32558645 -4.21900000
C -1.12412030 -0.60341355 -2.01200000
C 0.26987970 -2.08041355 -0.64700000
H 0.77987970 -2.22141355 0.14200000
C 0.98387970 3.31758645 -2.84700000
H 1.56387970 3.86458645 -2.33100000
C -1.78212030 0.74258645 -2.23600000
H -2.59912030 0.62258645 -2.78200000
H -2.04912030 1.12858645 -1.36500000
C 1.59787970 3.05558645 -8.80400000
C -1.55512030 -5.73441355 -6.78100000
H -2.39212030 -5.22041355 -6.90400000
H -1.75812030 -6.69441355 -6.91000000
C 2.03687970 -2.29141355 -10.29800000
C 0.65987970 2.00858645 1.62400000
C 1.58087970 3.51258645 -6.48900000
C 1.81687970 2.12158645 2.36700000
H 2.64987970 1.85358645 1.99700000
C 1.51387970 -5.73341355 -9.03500000
H 2.18787970 -6.34541355 -9.30500000
C 1.97587970 4.04758645 -5.14200000
H 1.84487970 5.02858645 -5.09400000
H 2.91887970 3.83358645 -4.93300000
C 0.68587970 -4.48041355 -1.61300000
H 1.67587970 -4.47441355 -1.59700000
H 0.35087970 -5.01441355 -0.84900000
C 2.54487970 -3.70941355 -10.31100000
H 2.55787970 -4.07741355 -11.23000000
H 3.45387970 -3.76841355 -9.92400000
C 2.56187970 -1.15641355 -10.89700000
H 3.37087970 -1.18941355 -11.39300000
C 1.18587970 -9.68441355 -6.56300000
C 3.66187970 3.25658645 -12.17000000
C -0.60212030 2.88558645 3.44000000
H -1.43312030 3.14858645 3.81900000
C -0.56012030 2.39558645 2.16100000
H -1.35612030 2.32258645 1.64800000
C 0.55587970 2.99758645 4.18000000
H 0.51687970 3.33558645 5.06600000
C 3.73787970 4.63358645 -12.10700000
H 3.48287970 5.08258645 -11.31000000
C 1.75987970 2.62558645 3.64500000
H 2.55487970 2.71458645 4.15700000
C 2.14787970 -10.33741355 -5.80700000
H 2.73887970 -9.83741355 -5.25800000
C 0.33687970 -10.42041355 -7.36800000
H -0.31012030 -9.98041355 -7.90600000
C 4.02987970 2.62058645 -13.33300000
H 3.98187970 1.67358645 -13.39500000
C 4.17787970 5.36258645 -13.18800000
H 4.23687970 6.30958645 -13.13200000
C 2.24887970 -11.71341355 -5.85100000
H 2.91587970 -12.15741355 -5.34200000
C 1.38987970 -12.43241355 -6.62400000
H 1.44787970 -13.38041355 -6.63900000
C 0.43287970 -11.79241355 -7.38900000
H -0.16112030 -12.30041355 -7.93000000
C 4.52987970 4.72358645 -14.34200000
H 4.81187970 5.22458645 -15.09800000
C 4.47187970 3.36358645 -14.40600000
H 4.73887970 2.92158645 -15.20300000
C 3.70187970 -5.12041355 -4.61000000
H 4.59887970 -5.05641355 -4.99900000
H 3.15387970 -5.72641355 -5.15000000
H 3.76687970 -5.46641355 -3.69500000
F 1.57587970 -0.07441355 -4.64800000
C 3.11987970 -3.87041355 -4.58800000
C 3.32387970 -3.01341355 -3.52600000
H 3.83787970 -3.30341355 -2.78100000
C 2.79287970 -1.74241355 -3.53100000
H 2.93087970 -1.15341355 -2.79800000
C 2.35787970 -3.42641355 -5.66000000
H 2.19687970 -4.00841355 -6.39300000
C 2.06387970 -1.34841355 -4.61600000
C 1.83287970 -2.16341355 -5.67600000
H 1.31787970 -1.86341355 -6.41500000

Supplementary Table 5. Coordinates of the fully optimized structure of 1,4-bis(4-cyanostyryl)benzene @ **F[3]A1-[P(O)Ph]$_3$**

P	0.21621620	0.71171170	0.00000000	C	2.48321620	-6.43228830	-1.76400000	H	-2.14078380	2.24571170	0.19500000
P	0.44721620	-10.12928830	-3.86800000	H	3.34721620	-6.04128830	-1.82200000	C	0.27221620	-11.88128830	-3.62100000
P	4.54421620	-1.21928830	-9.79500000	C	4.82721620	-3.63128830	-8.81500000	C	-0.93078380	-12.49928830	-3.93100000
O	0.48721620	-0.60928830	0.83400000	C	5.64121620	-1.25828830	-6.62700000	H	-1.66078380	-11.98428830	-4.25500000
O	1.65221620	1.35871170	-0.25800000	C	2.39521620	1.70871170	-2.50700000	C	-0.00178380	-14.60228830	-3.27100000
O	1.79121620	-10.06928830	-4.70500000	H	1.86021620	2.49271170	-2.55100000	H	-0.09778380	-15.53928830	-3.14500000
O	-0.57878380	0.51071170	-1.21000000	C	5.17521620	-0.25128830	-7.47200000	C	0.15921620	1.90771170	2.50000000
O	5.20121620	-2.67128830	-9.79000000	C	3.90821620	-7.07728830	-4.92100000	H	1.00621620	1.50971170	2.66100000
O	5.47021620	-0.32928830	-8.85100000	H	4.52021620	-6.58128830	-4.39000000	C	4.93421620	-0.59428830	-11.40700000
O	0.79721620	-9.63628830	-2.40200000	C	2.38521620	-8.88328830	-5.21400000	C	-1.69178380	3.22771170	3.26100000
O	3.12621620	-1.20628830	-9.41600000	C	2.76821620	-7.41828830	-7.04200000	H	-2.11178380	3.73071170	3.94800000
O	-0.67878380	-9.41528830	-4.46400000	C	0.99021620	-8.27328830	-2.06000000	C	-2.31378380	3.08671170	2.03300000
C	4.01621620	-0.57128830	-2.32400000	C	-0.83378380	-5.21828830	-0.63200000	H	-3.15678380	3.49571170	1.87900000
H	4.58321620	-1.33028830	-2.26500000	H	-1.24578380	-5.52628830	0.21500000	C	1.18021620	-13.99928830	-2.95800000
C	2.11021620	-2.11828830	-0.10800000	H	-1.54878380	-5.02328830	-1.28800000	H	1.90021620	-14.51728830	-2.61600000
C	2.40021620	-3.38628830	-0.62000000	C	3.60021620	-5.58928830	-8.27900000	C	-1.06278380	-13.87128830	-3.77100000
H	3.29021620	-3.61028830	-0.86600000	C	3.92321620	-4.60828830	-9.20200000	H	-1.87678380	-14.30328830	-4.00200000
C	4.55721620	-0.09128830	-4.81300000	H	3.53721620	-4.60528830	-10.07100000	C	-0.46878380	2.64271170	3.49200000
C	2.43021620	0.93571170	-1.35600000	C	3.38221620	-8.49228830	-2.90600000	H	-0.05078380	2.74471170	4.34000000
C	3.23421620	-0.20028830	-1.22900000	H	3.28821620	-9.46528830	-2.75400000	C	6.19821620	-0.24428830	-11.75900000
C	0.77921620	-1.84428830	0.23200000	H	4.26821620	-8.20528830	-2.57100000	H	6.91221620	-0.38628830	-11.14800000
C	1.42421620	-5.69328830	-1.25800000	C	4.11221620	0.90471170	-5.70000000	C	5.47721620	0.49071170	-13.88100000
C	3.96621620	0.16171170	-3.49700000	C	2.13421620	-8.54128830	-6.52700000	H	5.67721620	0.78671170	-14.76200000
C	-0.24778380	-2.76328830	0.09800000	H	1.54421620	-9.05728830	-7.06400000	C	6.45821620	0.31871170	-13.01300000
H	-1.13678380	-2.53928830	0.34700000	C	6.37221620	-2.46228830	-7.16400000	H	7.34221620	0.57971170	-13.24100000
C	0.05021620	-4.01128830	-0.40600000	H	6.99821620	-2.79728830	-6.47300000	C	3.89321620	-0.30428830	-12.26900000
C	3.18721620	-1.07428830	0.01200000	H	6.90821620	-2.19228830	-7.95200000	H	2.99721620	-0.48028830	-12.00700000
H	4.06421620	-1.51728830	0.13700000	C	-0.07878380	-7.55328830	-1.57400000	C	4.15521620	0.24271170	-13.52300000
H	3.01221620	-0.50928830	0.80600000	H	-0.94278380	-7.94428830	-1.51700000	H	3.44621620	0.44171170	-14.12200000
C	1.38121620	-4.31728830	-0.76500000	C	3.25621620	-8.18028830	-4.38400000	C	0.17321620	-0.56028830	-4.38800000
C	3.66521620	-6.70028830	-6.23600000	C	4.43021620	0.83571170	-7.04000000	H	-0.44978380	-0.12728830	-3.81700000
C	5.32421620	-1.15228830	-5.27100000	H	4.14721620	1.50871170	-7.64800000	C	-0.84678380	2.99871170	-7.69300000
H	5.63621620	-1.80928830	-4.65900000	C	3.23521620	1.89271170	-4.97900000	C	2.72421620	-3.24028830	-4.20200000
C	5.08521620	-4.57528830	-6.64800000	H	2.33921620	1.94671170	-5.39500000	C	1.93321620	-1.87728830	-6.08200000
H	5.47021620	-4.57428830	-5.78000000	H	3.64421620	2.79471170	-4.96900000	H	2.52021620	-2.34528830	-6.66400000
C	-0.46478380	1.75971170	1.26300000	C	2.67221620	-6.77928830	-8.40700000	C	1.84421620	-2.24228830	-4.73400000
C	4.19021620	-5.56628830	-7.00400000	H	1.74521620	-6.49128830	-8.60400000	N	3.46421620	-4.00128830	-3.76100000
C	0.13621620	-6.23528830	-1.16700000	H	2.97821620	-7.39728830	-9.11600000	C	-0.17378380	1.76571170	-7.25500000
C	3.15721620	1.31271170	-3.58700000	C	1.33621620	-12.63028830	-3.13400000	H	0.53021620	1.44571170	-7.80700000
C	2.28621620	-7.74528830	-2.18600000	H	2.16221620	-12.21028830	-2.92300000	C	0.94421620	-1.58128830	-3.89200000
C	5.42821620	-3.57328830	-7.56100000	C	-1.70978380	2.34971170	1.03600000	H	0.86621620	-1.83828830	-2.98000000

C	1.17221620	-0.84828830	-6.55700000
H	1.24421620	-0.60228830	-7.47100000
C	0.28821620	-0.14428830	-5.72500000
C	-0.44678380	1.05871170	-6.17500000
H	1.17778380	1.34471170	-5.64100000
C	-1.78178380	3.68171170	-6.91300000
H	-2.01278380	3.33971170	-6.05700000
C	0.53378380	3.53771170	-8.93900000
H	0.10121620	3.09871170	-9.49100000
C	-3.08778380	8.94171170	-11.91100000
H	-2.46578380	8.50871170	-12.48300000
C	-2.06778380	5.38271170	-8.60700000
C	-5.63878380	11.62271170	-12.09700000
C	-4.84778380	10.25871170	-10.21700000
H	-5.43478380	10.72771170	-9.63500000
C	-4.75978380	10.62371170	-11.56600000
N	-6.37878380	12.38271170	-12.53900000
C	-2.74078380	6.61571170	-9.04500000
H	-3.44478380	6.93571170	-8.49300000
C	-3.85878380	9.96271170	-12.40800000
H	-3.78078380	10.21971170	-13.31900000
C	4.08778380	9.23071170	-9.74300000
H	-4.15878380	8.98371170	-8.82900000
C	-3.20278380	8.52571170	-10.57500000
C	-2.46778380	7.32271170	-10.12400000
H	-1.73678380	7.03671170	-10.65900000
C	-1.13278380	4.69971170	-9.38700000
H	-0.90178380	5.04171170	-10.24300000
C	-2.38078380	4.84371170	-7.36000000
H	-3.01578380	5.28371170	-6.80900000

Supplementary Table 6. Coordinates of the fully optimized structure of crisaborole @ **F[3]A1-[P(O)Ph]$_3$**.

P -0.97297292 -0.99999999 0.00000000
P -2.45997292 6.87100001 7.96700000
P 1.92802708 9.28800001 -2.24500000
O -1.21897292 7.86000001 7.80500000
O -1.84397292 5.45500001 8.35600000
O -0.75597292 -1.23599999 1.55400000
O 0.48402708 -0.83499999 -0.61600000
O -3.34097292 6.85800001 6.80000000
O 3.03002708 8.17200001 -2.53700000
O -1.88197292 0.10500001 -0.32600000
O 2.37002708 9.94700001 -0.86200000
O 0.55302708 8.79700001 -2.28800000
C -1.40097292 4.55300001 7.35400000
C 2.22502708 2.92600001 -0.76300000
C -0.56497292 2.80600001 5.41700000
C 0.69502708 1.47800001 3.57700000
H 1.51102708 1.95300001 3.68800000
C -0.10697292 4.68700001 6.86000000
C 2.88402708 6.86600001 -2.01500000
C -1.86397292 2.70700001 5.93100000
C 3.23502708 5.27700001 -0.26700000
H 3.57002708 5.04200001 0.59000000
C -0.40397292 1.80000001 4.36700000
C 2.36402708 2.17100001 0.39300000
H 2.85502708 2.51200001 1.13200000
C 1.77602708 0.90200001 0.46000000
C 1.80102708 0.11800001 1.75100000
H 2.63702708 0.31900001 2.24200000
H 1.79002708 -0.85099999 1.54900000
C 1.46802708 3.44700001 -2.97000000
H 1.95702708 3.14400001 -3.77500000
H 0.53102708 3.65200001 -3.21500000
C 3.37302708 6.59600001 -0.73700000
C 1.53402708 2.42000001 -1.87000000
C 0.31802708 3.77500001 5.89100000
H 1.20602708 3.81500001 5.55700000
C -0.53197292 7.94700001 6.57500000
C 0.60802708 0.46600001 2.62300000
C 2.14002708 4.63300001 -2.33500000
C 2.42702708 7.87700001 2.18200000
H 2.90802708 7.15000001 2.56000000
C 1.38302708 8.45800001 2.88800000
C 2.28302708 5.92500001 -2.82900000
H 1.97702708 6.15400001 -3.69900000
C 0.48202708 7.02600001 6.30400000
C 1.08902708 0.44400001 -0.65500000
C -2.28497292 3.57600001 6.93200000
H -3.15197292 3.50100001 7.31300000
C -3.16797292 7.40300001 9.51100000
C -0.87597292 8.96900001 5.70200000
H -1.56697292 9.58500001 5.91700000
C 2.61002708 4.32100001 -1.05200000
C 1.16802708 7.13900001 5.10700000
H 1.86402708 6.52700001 4.90000000
C 0.67102708 9.54900001 2.35500000
C -0.18097292 9.06200001 4.50400000
C 0.77002708 5.86100001 7.24400000
H 1.72402708 5.60400001 7.17800000
H 0.58402708 6.12900001 8.17900000
C 0.98602708 10.02200001 1.09600000
H 0.50902708 10.75000001 0.71500000
C 0.83902708 8.14700001 4.20900000
C -0.34397292 10.04700001 3.36200000
H -1.26197292 10.01800001 2.99100000
H -0.13897292 10.97100001 3.65200000
C -2.62997292 1.61800001 5.21900000
H -3.42697292 1.98200001 4.75800000
H -2.91397292 0.90800001 5.84800000
C -1.71997292 0.09400001 3.27700000
H -2.53097292 -0.38899999 3.17200000
C -1.61097292 1.09700001 4.22600000
C 0.95602708 1.16300001 -1.83200000
H 0.48802708 0.80900001 -2.57900000
C -0.62597292 -0.18599999 2.48900000
C 2.01902708 9.40400001 0.40200000
C 3.90402708 7.69600001 0.14700000
H 4.36502708 8.37300001 -0.40900000
H 4.56202708 7.32000001 0.78300000
C 2.77402708 8.35600001 0.91700000
C -1.43797292 -2.60699999 -0.57500000
C -0.52397292 -3.65599999 -0.57000000
H 0.36102708 -3.51499999 -0.25600000
C 2.36202708 10.56500001 -3.40600000
C -0.90897292 -4.90799999 -1.02500000
H -0.28397292 -5.62299999 -1.03500000
C -2.75597292 6.92500001 10.73400000
H -2.06897292 6.27100001 10.78400000
C -2.19397292 -5.11399999 -1.46100000
H -2.45797292 -5.97599999 -1.75400000
C -4.19197292 8.33300001 9.45300000
H -4.49697292 8.64900001 8.61100000
C -4.34997292 8.34500001 11.82800000
H -4.74397292 8.67800001 12.62500000
C 1.35502708 11.15800001 -4.14200000
H 0.45702708 10.86000001 -4.05400000
C 3.65302708 10.99500001 -3.56100000
H 4.34902708 10.58200001 -3.06500000
C -2.72497292 -2.80999999 -1.04300000
H -3.34297292 -2.08799999 -1.06900000

C 2.94902708 12.62200001 -5.15000000
H 3.15002708 13.33800001 -5.74200000
C -4.77297292 8.80400001 10.62000000
H -5.46897292 9.44800001 10.57500000
C 1.66602708 12.19500001 -5.01300000
H 0.97502708 12.60900001 -5.51800000
C -3.35397292 7.40200001 11.89800000
H -3.06997292 7.07600001 12.74400000
C -3.10697292 -4.07199999 -1.47600000
H -3.99397292 -4.22099999 -1.78200000
C 3.95302708 12.02400001 -4.43900000
H 4.85102708 12.31500001 -4.54400000
O -5.39397292 9.55100001 -6.22400000
H -5.45097292 9.75300001 -7.03700000
O -3.40897292 8.50700001 -7.02700000
O -2.54897292 4.75300001 -2.47900000
N 1.46802708 4.71500001 2.86300000
C -0.15797292 4.84400001 0.87100000
C -0.87297292 3.69300001 0.50900000
H -0.80297292 2.90500001 1.03500000
C 0.73402708 4.79300001 1.99600000
C -0.26997292 6.00100001 0.10500000
H 0.20802708 6.78400001 0.35500000
C -1.67197292 3.70500001 -0.59900000
H -2.15597292 2.92700001 -0.85000000
C -1.07497292 6.01200001 -1.01800000
H -1.14797292 6.79600001 -1.54900000
C -3.96697292 7.88900001 -4.78700000
C -1.76997292 4.87200001 -1.36000000
C -2.86097292 7.14400001 -5.19500000
C -4.51497292 7.64600001 -3.53900000
H -5.24897292 8.16500001 -3.23300000
C -2.93597292 5.88900001 -3.19300000
C -2.32397292 6.12800001 -4.40200000
H -1.56997292 5.62500001 -4.68500000
C -3.99297292 6.64700001 -2.73600000
H -4.36197292 6.48400001 -1.87500000
B -4.33797292 8.74600001 -6.03600000
C -2.45397292 7.51800001 -6.58400000
H -1.53797292 7.89300001 -6.58800000
H -2.47497292 6.72500001 -7.17600000

Supplementary Table 7. Coordinates of the fully optimized structure of alectinib @ **F[3]A1-[P(O)Ph]$_3$**.

P -0.46992481 2.72556387 0.00000000

P 1.57707519 6.06456387 10.80200000

P 2.00207519 -4.63743613 8.14900000

O -1.26092481 3.87356387 0.76000000

O 1.19007519 -4.22143613 9.44900000

O 0.52707519 6.68056387 9.77200000

O 0.91107519 -4.89043613 7.01900000

O -1.33892481 1.39956387 0.12300000

O 0.75107519 5.05556387 11.70500000

O 3.04907519 -3.68743613 7.77400000

O 0.90807519 2.57256387 0.46900000

O 2.76907519 5.50556387 10.18500000

C 0.05607519 1.14556387 10.55400000

C -1.94492481 3.29256387 2.99300000

C -0.98192481 4.47956387 4.85000000

C 0.12707519 -0.28643613 10.20200000

C -1.28592481 4.50756387 7.42000000

H -1.97092481 3.84856387 7.41600000

C -0.22192481 -1.27343613 2.36900000

C 0.51707519 3.72256387 11.30600000

C -1.01892481 -0.99943613 3.49600000

C -0.59392481 3.45256387 10.50500000

C -0.65392481 -1.94143613 4.55900000

C -0.71492481 4.90856387 6.23500000

C 0.36507519 -2.78043613 4.08100000

C 1.27907519 -0.82143613 10.79000000

C -1.86792481 3.52656387 4.36800000

H -2.41792481 3.03656387 4.96900000

C 0.40507519 -3.86043613 6.19700000

C -1.89492481 0.05856387 3.50800000

H -2.42892481 0.22356387 4.27700000

C -1.13092481 -3.20143613 8.11500000

H -2.07892481 -2.91743613 8.15500000

H -1.08592481 -4.15343613 8.38400000

C -2.82092481 2.17156387 2.46000000

H -3.59892481 2.03856387 3.05800000

H -3.15692481 2.40656387 1.55900000

C -1.15392481 -2.08243613 5.85200000

H -1.84992481 -1.51443613 6.15700000

C -1.99992481 0.89756387 2.39000000

C 0.90307519 -3.74743613 4.91200000

H 1.59707519 -4.31943613 4.60800000

C 1.39907519 2.75656387 11.74600000

H 2.13607519 2.97856387 12.30100000

C -0.16292481 5.21456387 3.99200000

C -0.82192481 2.12356387 10.14800000

H -1.58192481 1.89456387 9.62500000

C 0.14107519 6.03456387 8.57100000

C -0.62692481 -3.06043613 6.69500000

C 0.29607519 5.88856387 6.20700000

C 1.16807519 1.45056387 11.34900000

C -1.22692481 0.58156387 1.27800000

C 1.63207519 -2.13243613 10.54000000

H 2.40207519 -2.51643613 10.94300000

C 0.82707519 -2.87343613 9.67800000

C -1.43792481 4.57856387 9.95100000

H -1.46092481 5.32656387 10.60000000

H -2.36592481 4.26156387 9.81400000

C -0.32292481 -2.37243613 9.08200000

C 0.71107519 -2.42443613 2.65700000

H 0.54207519 -3.18443613 2.04600000

H 1.65907519 -2.14743613 2.57600000

C -0.67292481 -1.05343613 9.36900000

H -1.46192481 -0.68043613 8.99400000

C -0.73692481 3.18456387 -1.69800000

C 0.72107519 6.16256387 4.78200000

H 1.68107519 5.95656387 4.64600000

H 0.55107519 7.10356387 4.53100000

C -0.86592481 5.06556387 8.63400000

C 0.72507519 6.45656387 7.39900000

H 1.40407519 7.12056387 7.40500000

C -1.13592481 4.04856387 2.16000000

C 1.78607519 7.43356387 11.91900000

C -1.11392481 2.26256387 -2.65600000

H -1.26492481 1.35956387 -2.40600000

C 2.01707519 0.22856387 11.59000000

H 2.04907519 -0.00643613 12.55100000

H 2.94007519 0.35856387 11.25300000

C 2.53707519 -6.27443613 8.56500000

C -0.35992481 -0.49443613 1.23300000

H 0.12807519 -0.69443613 0.44300000

C -0.51292481 4.50256387 -2.07900000

H -0.23392481 5.14456387 -1.43600000

C -0.23892481 5.01056387 2.62100000

H 0.30107519 5.50956387 2.01900000

C 0.69207519 8.06256387 12.47200000

H -0.18392481 7.75656387 12.27000000

C 1.64707519 -7.21543613 9.06800000

H 0.73607519 -6.98443613 9.19800000

C -1.08092481 3.95556387 -4.33700000

H -1.21692481 4.22456387 -5.23900000

C -0.69492481 4.87856387 -3.39800000

H -0.55292481 5.78256387 -3.65600000

C 3.07007519 7.87356387 12.21100000

H 3.82707519 7.43756387 11.83700000

C 3.23107519 8.96256387 13.06200000

H 4.10307519 9.27356387 13.27500000

C -1.27392481 2.64556387 -3.97600000

H -1.51392481 2.00256387 -4.63300000

C 0.86607519 9.14056387 13.31900000
H 0.11507519 9.56956387 13.71100000
C 2.13907519 9.58556387 13.58900000
H 2.26007519 10.34156387 14.15300000
C 2.08607519 -8.48043613 9.37800000
H 1.47107519 -9.12443613 9.71000000
C 3.87007519 -6.60843613 8.42000000
H 4.48607519 -5.96043613 8.09400000
C 3.39307519 -8.81643613 9.21200000
H 3.68307519 -9.69843613 9.41600000
C 4.31407519 -7.87443613 8.74700000
H 5.23407519 -8.10143613 8.65500000
O 6.16607519 2.67156387 9.68200000
N 3.97007519 -1.07043613 8.18300000
H 3.79107519 -1.90343613 7.96100000
O 15.00307519 -6.49443613 14.31700000
N 13.43007519 -4.29343613 13.32200000
N 10.50907519 -1.60143613 11.67200000
N -1.21992481 1.26156387 6.57500000
C 5.09107519 -0.65343613 8.83300000
C 6.15807519 -1.57943613 9.29500000
C 3.79907519 1.17256387 8.42700000
C 5.04707519 0.71856387 8.98400000
C 3.16007519 0.01156387 7.92900000
C 7.27107519 -0.78043613 9.97900000
C 7.23907519 0.60356387 10.14400000
C 5.53207519 -2.56143613 10.30800000
H 5.14307519 -2.05643613 11.05300000
H 6.22507519 -3.16543613 10.64900000
H 4.83207519 -3.08543613 9.86600000
C 1.87807519 2.44156387 7.80100000
H 1.42207519 3.27256387 7.73300000
C 12.13507519 -3.73843613 12.86400000
H 11.41907519 -4.40943613 13.05600000
C 3.13907519 2.39856387 8.35700000
H 3.55007519 3.18856387 8.68500000
C 8.35907519 -1.47943613 10.50000000
H 8.39207519 -2.42343613 10.38600000
C 6.14007519 1.44756387 9.61000000
C -0.10892481 1.29256387 6.88200000
C 1.25807519 1.27156387 7.33300000
C 1.90207519 0.03456387 7.37000000
H 1.49407519 -0.75243613 7.02800000
C 9.40207519 -0.85043613 11.18400000
C 8.29207519 1.23156387 10.83300000
H 8.26607519 2.17556387 10.93600000
C 11.78707519 -2.44143613 13.59800000
H 11.72707519 -2.62043613 14.56900000
H 12.50807519 -1.77743613 13.45100000
C 15.15307519 -5.99543613 12.98800000
H 15.43207519 -6.73743613 12.39300000
H 15.87007519 -5.31243613 12.97700000
C 10.47007519 -1.86543613 13.11100000
H 9.74007519 -2.50343613 13.30900000
H 10.28107519 -1.02443613 13.59500000
C 13.88607519 -5.38543613 12.45400000
H 14.04407519 -5.03743613 11.54200000
H 13.18107519 -6.08043613 12.40200000
C 6.72207519 -2.35043613 8.07800000
H 6.01707519 -2.90543613 7.68600000
H 7.46407519 -2.92143613 8.36800000
H 7.04507519 -1.71143613 7.40900000
C 9.36007519 0.54056387 11.36700000
C 10.77907519 -2.84543613 10.95000000
H 10.78907519 -2.66643613 9.97600000
H 10.05807519 -3.49643613 11.13600000
C 13.32007519 -4.79643613 14.68600000
H 12.59307519 -5.46843613 14.72800000
H 13.07807519 -4.04943613 15.28900000
C 12.12307519 -3.43843613 11.37300000
H 12.29107519 -4.27243613 10.86700000
H 12.84907519 -2.79943613 11.15900000
C 10.49407519 1.27356387 12.08300000
H 10.43907519 1.06256387 13.04700000
H 11.35407519 0.91356387 11.74900000
C 14.60807519 -5.42843613 15.17100000
H 15.32307519 -4.74343613 15.19700000
H 14.48007519 -5.77243613 16.09100000
C 10.52107519 2.75656387 11.93100000
H 10.58507519 2.98756387 10.98100000
H 11.29607519 3.11956387 12.40900000
H 9.70007519 3.13856387 12.30600000

3. Supplementary References

(1) Dolomanov, O. V.; Bourhis, L. J.; Gildea, R. J.; Howard, J. A. K.; Puschmann, H. **OLEX2**: a complete structure solution, refinement and analysis program. *J. Appl. Crystallogr.,* **2009**, *42*, 339-341.

(2) Du, X.-S.; Zhang, D.-W.; Guo, Y.; Li, J.; Han, Y.; Chen, C.-F. Towards the Highly Efficient Synthesis and Selective Methylation of C(sp^3)-Bridged [6] Cycloparaphenylenes from Fluoren[3]arenes. *Angew. Chem. Int. Edit.,* **2021**, *60*, 13021-13028.

(3) Yan Z., Donald G. T., Density Functional for Spectroscopy: No Long-Range Self-Interaction Error, Good Performance for Rydberg and Charge-Transfer States, and Better Performance on Average than **B3LYP** for Ground States, *J. Phys. Chem. A*, **2006**, 110,13126–13130.

(4) Stefan G., Stephan E., Lars G., Effect of the damping function in dispersion corrected density functional theory. *J. Comp. Chem.,* 32, **2011**, 1456-1465.
